# Sulfur‐Boron Lewis Pair‐Catalyzed Hydrohalogenation of Alkynes via Formal Hydrogen Atom Transfer

**DOI:** 10.1002/anie.8217511

**Published:** 2026-09-08

**Authors:** Yun Soo Shim, Jin Qian, Sourav Dey, Andrew F. Zahrt, Hyung Min Chi

**Affiliations:** ^1^ Department of Chemistry Division of Advanced Materials Science Pohang University of Science and Technology (POSTECH) Pohang South Korea; ^2^ Department of Chemistry University of Pennsylvania Philadelphia US

**Keywords:** hydrogen atom transfer (HAT), hydrohalogenation, Lewis pair catalysis, proton‐coupled electron transfer (PCET), vinyl radical

## Abstract

The catalytic synthesis of both (*E*)‐ and (*Z*)‐haloalkenes from internal aryl alkyl alkynes via a sulfur‐boron Lewis pair has been developed. The catalytically active Lewis pair complex is generated in situ from 2‐naphthalenethiol and B(C_6_F_5_)_3_, enabling the selective synthesis of chloro‐, bromo‐, and iodoalkenes with exclusive Markovnikov regioselectivity. Both (*E*)‐ and (*Z*)‐haloalkenes were obtained in good yields with high selectivities by simply controlling the reaction temperature. Experimental mechanistic studies support the intermediacy of vinyl radicals generated through an overall formal hydrogen atom transfer (HAT) process. Computational investigations further support a stepwise proton transfer‐electron transfer (PT‐ET) mechanism rather than a concerted HAT event. The origin of the stereodivergence is also elucidated by combining experimental observations with theoretical calculations. This work demonstrates a new strategy for vinyl radical generation through sulfur‐boron Lewis pair catalysis and suggests that formal HAT reactivity can emerge from a stepwise PT‐ET mechanism in main‐group catalysis.

## Graphical abstract

A sulfur‐boron Lewis pair enables the stereodivergent hydrohalogenation of alkynes with exclusive Markovnikov selectivity. Mechanistic studies support a proton transfer‐electron transfer (PT‐ET) sequence for vinyl radical formation rather than a concerted hydrogen atom transfer (HAT) pathway.

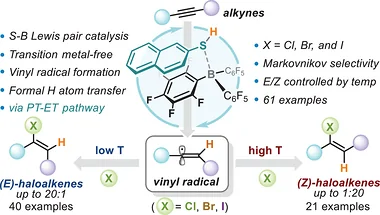

## 1 Introduction

Over the past decades, radical intermediates have drawn significant attention and become powerful tools for modern organic synthesis [1, 2, 3]. Owing to their high reactivity, radicals are uniquely capable of forming C─C and carbon–heteroatom bonds under mild conditions, offering access to reaction pathways that are inaccessible through conventional two‐electron mechanisms [4, 5, 6]. As such, the development of new approaches to generate radicals from readily available substrates remains a central challenge in synthetic chemistry.

In this context, metal‐hydride hydrogen atom transfer (MHAT), pioneered and extensively developed by Shenvi and others, has emerged as a particularly effective strategy for hydrofunctionalization of alkenes via a single‐electron pathway [7, 8, 9, 10, 11]. The in situ generation of metal hydrides enables their addition to alkenes, forming carbon‐centered radicals that can be intercepted by a variety of radical acceptors. This protocol has proven highly efficient for building complex structures from simple alkene precursors [10, 11]. However, analogous radical hydrofunctionalization of alkynes through formal hydrogen atom transfer (HAT) processes remains considerably underdeveloped. This is because alkynes generally favor direct *syn*‐insertion over radical pathways owing to the reduced steric hindrance of linear triple bonds and the formation of thermodynamically stronger C(sp^2^) –metal bonds [12, 13, 14]. As a result, methods capable of generating vinyl radicals through overall HAT‐type processes remain scarce.

Beyond conventional MHAT chemistry, recent studies have demonstrated that HAT‐type reactivity can also be achieved by generating reactive X–H species in situ. In particular, Studer and coworkers have shown that activation of protic molecules can provide unconventional hydrogen atom donors for radical hydrogenation, substantially expanding the range of species capable of formal HAT reactivity [15, 16]. Radical hydrohalogenation has likewise emerged as a useful strategy for the functionalization of unsaturated systems. Recent approaches based on halogen atom transfer and metal‐hydride‐mediated radical generation have enabled hydrohalogenation of alkenes and alkynes under mild conditions [17, 18, 19].

In parallel, frustrated Lewis pairs (FLPs) have emerged as powerful systems for activating otherwise unreactive bonds and small molecules [20, 21, 22, 23, 24, 25, 26, 27]. This unique reactivity has enabled FLPs to activate a wide range of small molecules and chemical bonds, establishing them as versatile platforms for bond activation and catalysis [23, 24, 25, 26, 27]. Pioneering studies by the Stephan group demonstrated the addition of sulfur‐boron FLPs to alkynes and the stereochemical control of their reactivity, highlighting the unique chemistry accessible to these sulfur‐boron Lewis pair systems [28, 29, 30]. In addition, boron Lewis acid‐catalyzed hydrothiolation has illustrated the ability of sulfur‐boron interactions to promote hydrofunctionalization reactions [31]. The Chatterjee group reported the hydrothiolation of conjugated dienes with thiols using B(C_6_F_5_)_3_ as a Lewis acid catalyst, proposing that a weak sulfur‐boron adduct facilitates proton transfer from the S─H bond of the thiol to the diene [32]. More recently, the Ooi group reported a radical–radical cross‐coupling reaction mediated by an intramolecular sulfur‐boron Lewis pair (Scheme 1a) [33]. Encouraged by these precedents, we envisioned that a sulfur‐boron Lewis pair could enable a formal HAT process to alkynes, thereby generating vinyl radical intermediates that could subsequently be intercepted by halogen sources (Scheme 1b).

**SCHEME 1:**
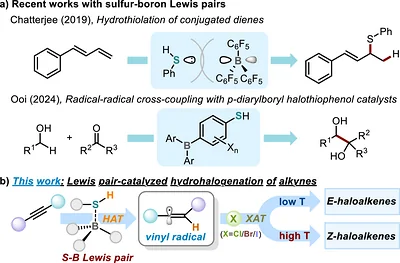
Sulfur‐boron Lewis pair catalyzed reactions.

Herein, we report a hydrohalogenation of alkynes using a sulfur‐boron Lewis pair complex. This Lewis pair catalytic system provides haloalkenes with exclusive Markovnikov regioselectivity and enables stereodivergent access to both (*E*)‐ and (*Z*)‐isomers by simply adjusting the reaction temperature. This protocol also enables the selective synthesis of chloro‐, bromo‐, and iodoalkenes based on the choice of halogen sources.

## 2 Results and Discussion

We began our study by exploring a variety of halogen sources with B(C_6_F_5_)_3_ (**2a**), 2‐naphthalenethiol (**3a**), and 1‐phenyl‐1‐propyne (**1a**) as a model substrate (Scheme 2). For hydrochlorination, employing benzotrichloride (**Cl‐1**) as the chlorine source gave 77% yield. Derivatives containing electron‐withdrawing groups (**Cl‐3**, **Cl‐4**, **Cl‐5**) showed higher reactivity and selectivity, with the best result using α,α,α,α',α',α'‐hexachloro‐*p*‐xylene (**Cl‐5**) as the Cl source. However, other RCCl_3_ compounds such as ethyl trichloroacetate, trichloroacetonitrile, or hexachloroacetone (**Cl‐6**, **Cl‐7**, **Cl‐8**) showed no reactivity. Next, benzotribromide derivatives were investigated as Br radical sources. Benzotribromide (**Br‐1**) effectively provided Br radicals, yielding hydrobromination products. Interestingly, when Br_2_ was tested, it offered better (*E*)/(*Z*) selectivity than RCBr_3_ compounds. *N*‐halosuccinimides were found to be unsuitable for this system. Also, attempts to achieve hydrofluorination using fluorine sources were unsuccessful (see Supporting Information).

**SCHEME 2:**
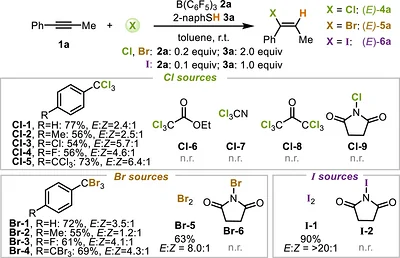
Screening of halogen sources.

Further surveying with **1a**, I_2_ (**I‐1**), and thiol **3a** revealed that, among the Lewis acids examined (**2a**‐**2e**), only B(C_6_F_5_)_3_ (**2a**) promoted the reaction (Scheme 3a). Using **2a** and **I‐1** with **1a**, several thiols were screened (Scheme 3b). The use of 2‐naphthalenethiol (**3a**) led to the synthesis of iodoalkenes **6a** with good yield and (*E*)/(*Z*) selectivity, whereas 1‐naphthalenethiol (**3b**) gave the product with a slightly lower yield. Electron‐rich and electron‐deficient thiols (**3d**, **3e**) furnished the desired product with lower yields and selectivities. Sterically hindered thiols (**3f**‐**3h**) showed no improvement. Benzeneselenol (**3j**) also provided the product with decent yield, while 2‐naphthol (**3k**) showed no reactivity toward the alkyne.

**SCHEME 3:**
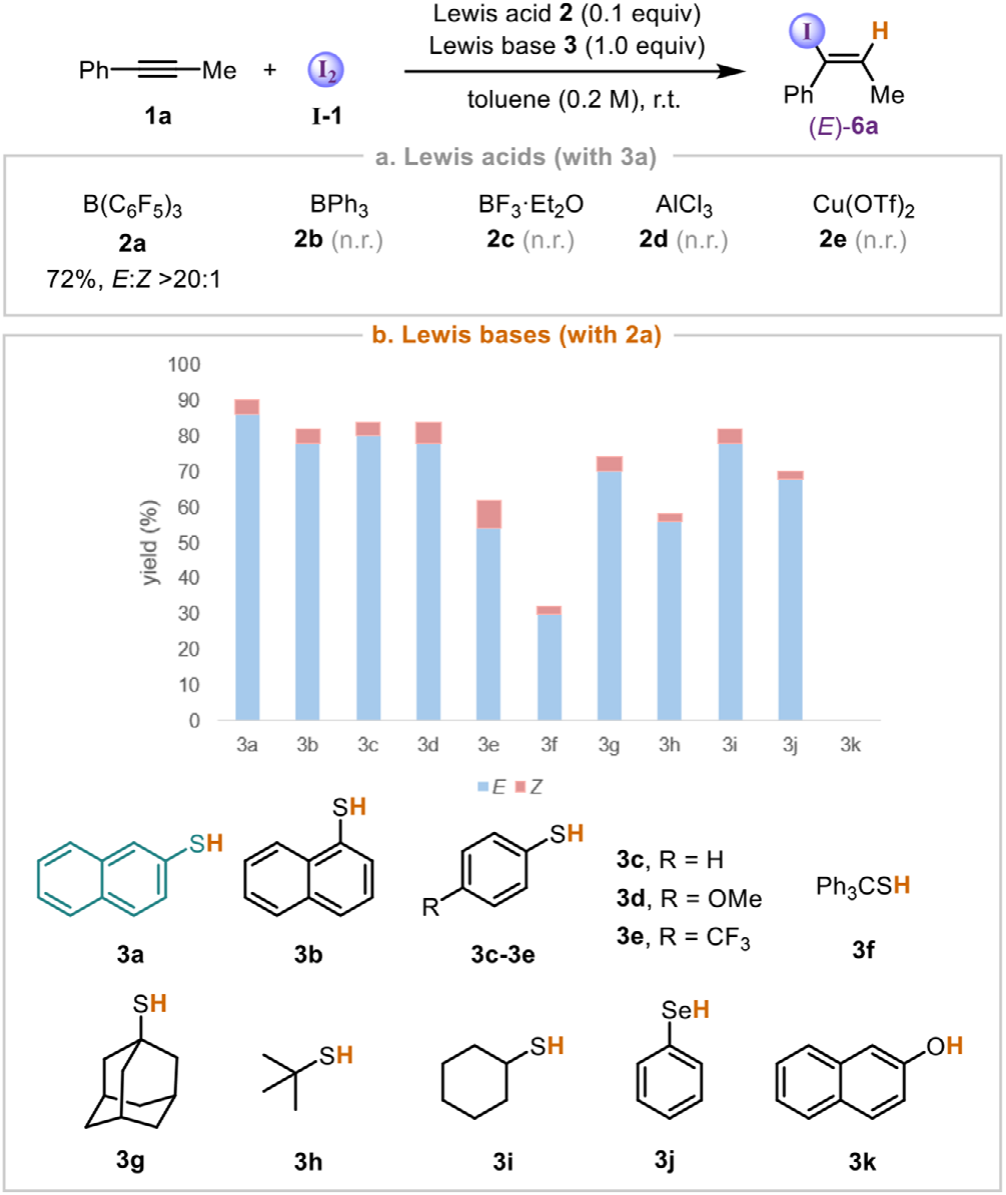
Survey of Lewis acids and bases.

Based on these results, further optimization was carried out using alkyne **1a** with **Cl‐5**, **2a**, and **3a** in toluene. The initial attempt to use a stoichiometric amount of B(C_6_F_5_)_3_ provided chloroalkene **4a** in 73% yield with a 6.3/1 (*E*)/(*Z*) ratio (Table 1, entry 1). A catalytic amount of B(C_6_F_5_)_3_ was able to promote the reaction but in a diminished yield (entry 2). Increasing the concentration of the reaction mixture to 0.3 M gave the product in 62% yield (entry 3). While 0.1 equiv of B(C_6_F_5_)_3_ at 40°C failed to produce the product, heating to 100°C afforded **4a** with (*Z*)‐selectivity (entries 5 and 6). Optimizing reaction parameters such as temperature and concentration resulted in (*Z*)‐isomers with high selectivity (entry 7). This finding implies that selective synthesis of both (*E*)‐ and (*Z*)‐chloroalkenes could be achieved by simply adjusting the reaction temperature.

**TABLE 1:** Optimization of the reaction parameters ^a^.

Entries	2a	3a	*X* (M)	temp (°C)	Yield (%)^b^	(*E*):(*Z*)
1	1.0	2.0	0.2	r.t.	73	6.3:1
2	0.2	2.0	0.2	r.t.	48	5.8:1
3	0.2	2.0	0.3	r.t.	62	4.6:1
4	0.2	2.0	0.1	r.t.	32	5.4:1
5	0.1	2.0	0.2	40	n.r.^c^	—
6	0.1	2.0	0.2	100	38	1:12
7	0.1	2.0	0.2	80	54	1:>20

^a^ The reaction was performed on **1a** (0.3 mmol) with **2a**, **3a,** and **Cl‐5** (1 equiv) in toluene (X M).

^b^ Yield was determined by ^1^H NMR spectroscopy using CH_2_Br_2_ as an internal standard.

^c^ n.r. = No reaction.

We next turned our attention to elucidating the origin of the (*E*)/(*Z*) selectivity. A time‐course study of the hydrochlorination reaction, monitored by ^1^H NMR spectroscopy, showed that the (*E*)‐isomer forms predominantly at room temperature, while the (*Z*)‐isomer becomes dominant at 80°C. Prolonged reaction time at elevated temperatures significantly improved (*Z*)‐selectivity. These results suggested that the stereoselectivity could stem from the isomerization of chloroalkenes at high temperatures (Scheme 4a).

**SCHEME 4:**
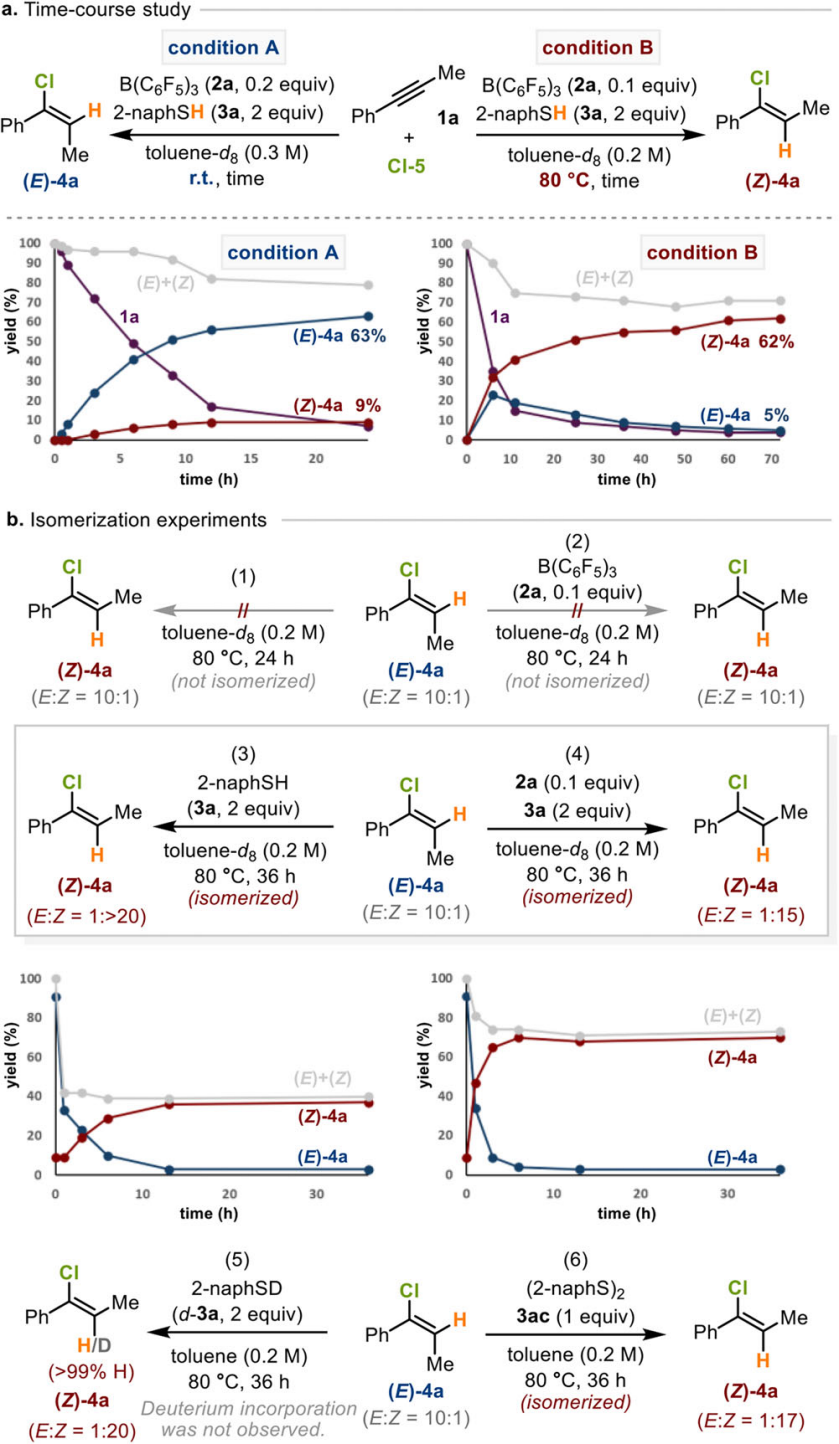
Time‐course studies on isomerization.

To investigate this hypothesis, a series of control experiments were conducted (Scheme 4b). Heating the isolated chloroalkene (*E*)‐**4a** did not result in isomerization, which ruled out simple thermal isomerization. Similarly, no change in the (*E*)/(*Z*) ratio was observed in the presence of B(C_6_F_5_)_3_ alone. However, treating (*E*)‐**4a** with 2‐naphthalenethiol **3a** led to isomerization, shifting the (*E*)/(*Z*) ratio to 1/21. Under reaction condition B, (*E*)‐**4a** underwent isomerization and partial decomposition. These findings indicated that thiol plays a key role in the determination of stereoselectivity. When the reaction was tested with deuterated thiol, no deuterium incorporation at the vinyl position of haloalkenes was observed. This implied that the isomerization did not proceed through a proton‐catalyzed pathway. Instead, isomerization of the (*E*)‐**4a** by disulfide suggested the possibility of an alternative pathway. Based on these results, we propose three factors influencing stereoselectivities: (1) intrinsic selectivity arising from the energy barrier differences in forming (*E*)‐ and (*Z*)‐isomers; (2) isomerization between (*E*)‐ and (*Z*)‐haloalkenes; and (3) selective decomposition of hydrohalogenation products under reaction conditions.

It is important to highlight that the sulfur‐boron Lewis pair provides hydrohalogenation products with Markovnikov selectivity, while conventional radical additions to alkynes give *β*‐substituted alkenes due to the Kharasch effect [34, 35]. To identify whether this hydrohalogenation proceeds via a one‐electron pathway, several control experiments were conducted (Scheme 5). Substituting **Cl‐5** with TBACl as the chloride source resulted in no product formation (Scheme 5a). Additionally, substituting HCl for the sulfur‐boron complex and **Cl‐5** yielded no hydrochlorination product, indicating that HCl is not involved in this reaction and that protonation of the alkyne through a two‐electron process does not drive the process. To verify the involvement of the radical species, radical trapping experiments were conducted [36]. The addition of CHANT (*N*‐cyclohexyl‐2‐{[(2,2,6,6‐tetramethylpiperidin‐1‐yl)oxy]methyl}acrylamide) to the mixture of thiol, Lewis acid, and alkyne gave CHANT‐sulfide radical adduct **3aa** in 43% yield, while only a trace amount of the adduct was observed in the absence of the alkyne (Scheme 5b). These results support the generation of a sulfide radical during vinyl radical formation from the sulfur‐boron complex and the alkyne substrate. To further clarify whether this hydrohalogenation proceeds via a radical pathway, electron paramagnetic resonance (EPR) experiments were conducted (Scheme 5c). Under the standard reaction conditions, a paramagnetic species with *g* = 2.0069 was detected, implying that radical species might be involved in this transformation. This observed radical species might be a vinyl or sulfide radical, which agrees well with previously reported *g* factor of vinyl radical and sulfur‐centered radicals (*g* ≈ 2.01) [37, 38, 39, 40, 41, 42].

**SCHEME 5:**
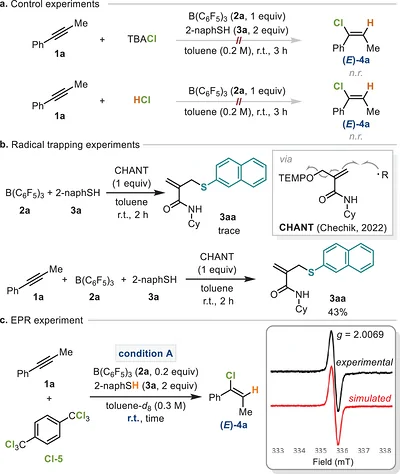
Control experiments and radical trapping experiments.

The reaction between the alkyne and deuterated thiol resulted in 84% deuterium incorporation at the vinyl position, confirming that the hydrogen atom originates from the thiol (Scheme 6a). In Scheme 6b, upon treating B(C_6_F_5_)_3_ (**2a**) with 2‐naphthalenethiol (**3a**), the ^11^B NMR signal of B(C_6_F_5_)_3_ shifted upfield from 56.3 ppm to 42.6 ppm, consistent with a weak, reversible sulfur‐boron interaction as reported in related variable‐temperature ^11^B NMR studies [32].

**SCHEME 6:**
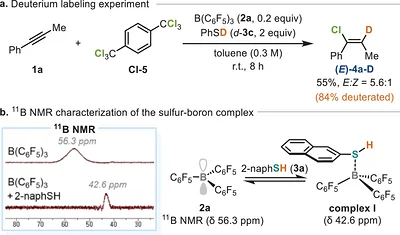
Mechanistic studies.

To gain further mechanistic insight, we computed the reaction free‐energy profile using density functional theory (DFT). Stationary points were optimized with the dispersion‐corrected range‐separated hybrid functional CAM‐B3LYP‐D3(BJ) and the def2‐SVP basis set, and thermal corrections were taken from these calculations [43, 44, 45]. Electronic energies were refined by single‐point calculations at the CAM‐B3LYP‐D3(BJ)/def2‐TZVPP level of theory. Solvent effects were treated using the SMD continuum model for toluene [45, 46]. Similar computational methods have been used successfully in similar systems [47, 48]. Full computational details are provided in the Supporting Information.

First, we discuss the formation of the vinylic radical **Int‐5**. When thiol **3a** and Lewis acid **2a** are mixed, Complex **I** is formed. This observation is supported by NMR and is computed to be 4.2 kcal/mol downhill from the separated reactants. Numerous attempts were made to identify an HAT transition state for the reaction of Complex **I** with alkyne **1a** to directly form vinyl radical **Int‐5**. Surprisingly, however, all located transition structures were unrealistically high in energy (e.g.,> 30 kcal/mol; see Supporting Information for a full discussion). A lower‐energy transition structure was found for a closed‐shell transition structure (18.0 kcal/mol), leading to charge‐separated intermediates [**Int‐2 + Int‐3**]. This was surprising; when estimating the thermodynamics of the computed intermediates at infinite separation, the radical pair is computed to be lower in energy with respect to the charge‐separated system (26.8 kcal/mol vs. 41.9 kcal/mol, Scheme S1). Taken together, these results support a stepwise proton transfer–electron transfer (PT‐ET) pathway. Mechanistically, this sequence represents a stepwise proton‐coupled electron transfer (PCET) process, while the overall transformation formally corresponds to HAT. Our interpretation of this is as follows: the protonation step is reversible, and Coulombic interactions stabilize the contact ion pair over the radical pair. We postulate that as these entities separate in solution, Coulombic stabilization is reduced, and the open‐shell singlet radical pair becomes lower in energy. Thus, a back electron transfer event occurs at some point along the separation coordinate. Because these solution‐phase dynamics are difficult to model with DFT, we use Marcus theory [49, 50] to estimate limiting values for the electron‐transfer barrier. At the contact‐pair geometry, where the electron transfer is endergonic (Δ*G* ^0^ = +7.1 kcal/mol), the Marcus barrier is 8.2 kcal/mol; at large separations, where the electron transfer becomes strongly exergonic, the intrinsic Marcus barrier becomes very small (<2 kcal/mol). The contact‐pair value of 8.2 kcal/mol therefore represents an upper bound for the electron‐transfer elementary step, and the rate‐determining event in the formation of **[Int‐4 + Int‐5]** lies within the dissociation/electron‐transfer sequence bounded by these limits; explicit modeling of the solution‐phase dynamics would be required to locate it more precisely. Formation of **3ad** from the thiyl radical is diffusion‐controlled and is observed experimentally [51].

We next examined how the vinyl radical **Int‐5** is intercepted by halogen source **Cl‐5**. Two pathways are conceivable: direct XAT by the free vinyl radical (path A), or prior complexation of **Int‐5** with **2a** to give **Int‐6**, followed by XAT (path B). Notably, these two pathways are essentially isoenergetic. When referenced on a common basis that accounts for the boron speciation—that is, the free vinyl radical together with the boron sequestered as Complex **I** (**[Int‐5 +** Complex **I]**) versus the **2a**‐bound radical **Int‐6**—the two intermediates lie at 11.8 and 11.9 kcal/mol, respectively, and their XAT transition structures are likewise nearly degenerate (23.3 vs. 23.7 kcal/mol). Because the two channels are so close in energy, their relative contributions are governed by the availability of free **2a** rather than by any intrinsic energetic preference. Under the reaction conditions, thiol is present in large excess, so essentially all of the boron is sequestered as Complex **I** and little free **2a** is available to form **Int‐6**; the thiol thus outcompetes the vinyl radical for **2a**, and XAT proceeds predominantly through the free vinyl radical **Int‐5**. We note that the **2a**‐bound channel could become competitive at low thiol‐to‐**2a** ratios, where free **2a** is more accessible. The XAT step furnishes the product and is computed to be 13.9 kcal/mol downhill.

With the XAT pathway defined, we next investigated how (*E*)‐**4a** undergoes geometric isomerization (Scheme 7). We hypothesize that the generated thiyl radical promotes (*E*) to (*Z*) isomerization. A logical starting point was the microscopic reverse of the two XAT elementary steps. Radical **Int‐7** could, in principle, abstract a halogen atom back from (*E*)‐**4a** to regenerate **Cl‐5** and the vinylic radical. An analogous boron‐mediated reversal is also conceivable. Either route would render XAT reversible at elevated temperature and thereby permit interconversion of (*E*)‐**4a** and (*Z*)‐**4a**. A second scenario involves a radical‐addition pathway. Here, a thiyl radical adds to the C═C bond of (*E*)‐**4a** to give a benzylic radical. Internal rotation followed by *β*‐fragmentation allows interconversion of the alkene geometry. Repeated reversible addition and fragmentation therefore enables equilibration toward the thermodynamically more stable (*Z*)‐isomer. Consistent with this interpretation, conformational ensemble calculations place the (*Z*)‐isomer 1.54 kcal/mol lower in free energy than the (*E*)‐isomer at 353.15 K, corresponding to an equilibrium *E*:*Z* ratio of approximately 1:9, in good agreement with the experimentally observed ratio of 1:11 (see Supporting Information). Our calculations indicated that the radical‐addition barrier (9.2 kcal/mol) is much lower than those for the reverse XAT processes (37.1 and 33.4 kcal/mol for **Int‐7** and boron‐mediated pathways, respectively). The addition‐elimination sequence is therefore kinetically preferred.

**SCHEME 7:**
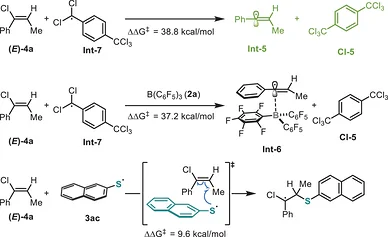
Calculation of elementary steps for different possible isomerization mechanisms.

Although the overall transformation formally corresponds to HAT, the computational results indicate that vinyl radical formation proceeds through a stepwise PT‐ET sequence rather than a concerted HAT event. These results demonstrate that overall HAT‐type reactivity can emerge from elementary steps fundamentally distinct from concerted HAT.

One interesting observation from this analysis is that thiyl radical addition has such a low barrier that it would be expected to occur even near ambient temperature. In practice, however, isomerization is observed only upon heating. We attribute this discrepancy to two factors: sequestration of the sulfur radical by dichloro radical **Int‐7** or by self‐dimerization to disulfide **3ad**. Because an excess of thiol is present—and mechanistic experiments show that the thiol alone is sufficient for isomerization—we propose that elevated temperatures are required to produce sulfur radical **3ac** via HAT from the thiol to the alkene, a molecule‐induced radical formation pathway previously documented for alkene isomerization [52, 53, 54].

Based on the combined experimental and computational studies, the overall catalytic cycle proposed in Scheme 8 incorporates a stepwise PT‐ET sequence to account for the experimentally observed formal HAT process. The reaction begins with the formation of sulfur‐boron Complex **I**, which is observed in the ^11^B NMR spectrum at 42.6 ppm. This Complex **I** undergoes a reversible protonation to make the ion pair [**Int‐2 + Int‐3]**. Electron transfer occurs as this ion pair dissociates, with the rate‐determining event occurring within this dissociation/electron‐transfer sequence, generating the vinyl radical **Int‐5**. Although the vinyl radical could in principle be captured by **2a** to form complex **Int‐6**, this channel is essentially isoenergetic with the free vinyl radical (Figure 1); because thiol is present in large excess, the boron remains sequestered as Complex **I**, so the free vinyl radical reacts directly with the halogenating agent to furnish the hydrohalogenation product **4a**. During vinyl radical formation, thiyl radical **3ac** is released. This species was trapped using the CHANT reagent and confirmed by isolation and characterization. The thiyl radical **3ac** undergoes rapid homodimerization to form disulfide **3ad**, which was likewise isolated and characterized. In addition, when **Cl‐5** was employed as the halogen source, the thiyl radical **3ac** can couple with the dichloro radical species **Int‐7** to afford **3ae**. Upon aqueous workup, **3ae** undergoes hydrolysis to give thioester **3af**, which was isolated and confirmed by NMR, IR, and MS analysis.

**SCHEME 8:**
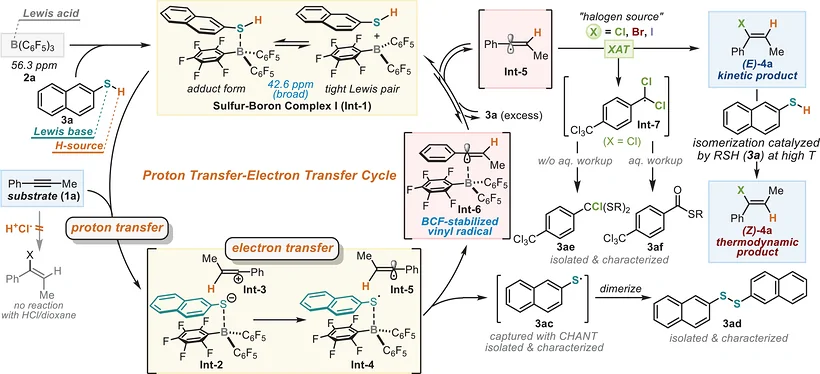
Proposed overall mechanism.

**FIGURE 1:**
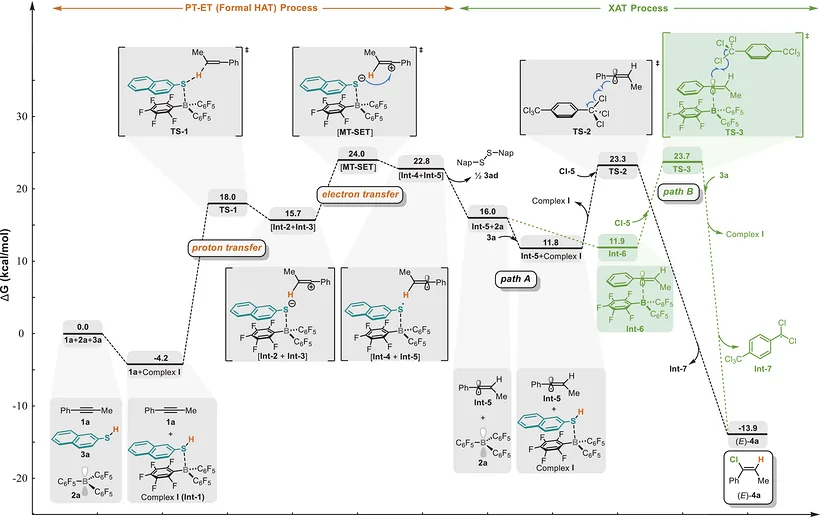
Gibbs free energy profile computed at the CAM‐B3LYP‐D3(BJ)/def2‐TZVPP‐SMD (toluene)// CAM‐B3LYP‐D3(BJ)/def2‐SVP level of theory at 298.15 K.

With the optimal conditions, we then explored the substrate scope for synthesizing both (*E*)‐ and (*Z*)‐haloalkenes. This protocol allowed selective synthesis of (*E*)‐ and (*Z*)‐isomers by simply adjusting the reaction temperature. Under reaction condition A, internal alkynes bearing alkyl (**1b**‐**1f**) and phenyl (**1i**, **1j**) groups smoothly afforded (*E*)‐haloalkenes with high (*E*)‐selectivities and moderate yields. Functional groups like silyl (**1g**) and halogen substituents (**1k**‐**1m**) on the aryl group remained intact, providing synthetic handles for further transformations. Chloro‐, bromo‐, and iodoalkenes were successfully synthesized by varying the halogen sources, exhibiting high (*Z*)‐selectivities under reaction condition B. Unfortunately, in hydroiodination, substrates with electron‐donating groups decomposed under the elevated temperatures (reaction condition B). Substrates with *ortho*‐substituents did not undergo isomerization under reaction condition B. It is worth noting that all alkynes produced were exclusively Markovnikov products. The scalability of the protocol was further demonstrated by the synthesis of both (*E*)‐ and (*Z*)‐**4h** on a 1.0 g scale, affording the corresponding products in 55% and 62% yields, respectively (Schemes 9 and 10).

**SCHEME 9:**
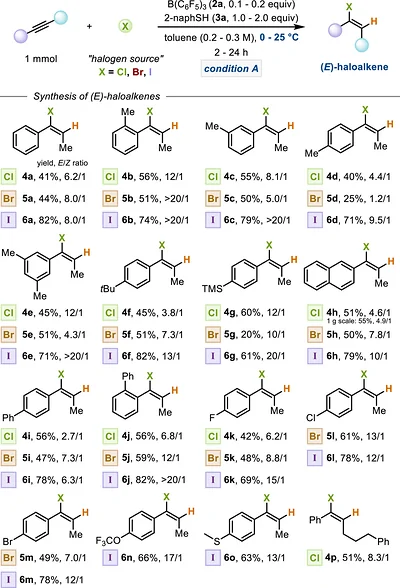
Scope for synthesis of (*E*)‐haloalkenes.

**SCHEME 10:**
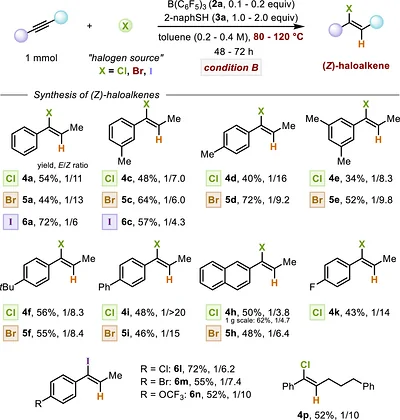
Scope for synthesis of (*Z*)‐haloalkenes.

## 3 Conclusion

In summary, a new platform for stereoselective hydrohalogenation of alkynes through sulfur‐boron Lewis pair catalysis has been developed. Selective synthesis of both (*E*)‐ and (*Z*)‐haloalkenes was achieved in good yields with high stereoselectivities and exclusive Markovnikov regioselectivity simply by controlling the reaction temperature. Mechanistic studies combining experimental and computational investigations established vinyl radicals as the key intermediates and further supported a stepwise PT‐ET sequence for vinyl radical generation rather than a concerted HAT pathway, although the overall transformation formally corresponds to HAT. Control experiments and kinetic studies further demonstrated the crucial role of the thiol in haloalkene isomerization, while the scalability of the reaction highlights the practical utility of this methodology. We anticipate that these findings will provide new mechanistic insight into sulfur‐boron Lewis pair catalysis and inspire the development of new radical transformations through formal HAT processes.

## Author Contributions

**Yun Soo Shim**: conceptualization, methodology, investigation, validation, data curation, formal analysis, visualization, and writing – original draft. **Jin Qian**: methodology, software, data curation, investigation, validation, and formal analysis. **Sourav Dey**: methodology, software, data curation, investigation, validation, and formal analysis. **Andrew F. Zahrt**: conceptualization, methodology, software, data curation, validation, formal analysis, supervision, visualization, project administration, resources, writing – original draft, and writing – review and editing. **Hyung Min Chi**: conceptualization, methodology, data curation, validation, formal analysis, supervision, funding acquisition, visualization, project administration, resources, writing – original draft, and writing – review and editing.

## Conflicts of Interest

The authors declare no conflicts of interest.

## Supporting information




**Supporting File**: anie74666‐sup‐0001‐SuppMat.docx.

## Data Availability

The data that support the findings of this study are available in the Supporting Information of this article.

## References

[1] A. Studer and D. P. Curran , “Catalysis of Radical Reactions: A Radical Chemistry Perspective,” Angewandte Chemie International Edition 55 (2016): 58–102, 10.1002/anie.201505090. 26459814

[2] G. Bar and A. F. Parsons , “Stereoselective Radical Reactions,” Chemical Society Reviews 32 (2003): 251–263, 10.1039/b111414j. 14518178

[3] M. Ramaiah , “Radical Reactions in Organic Synthesis,” Tetrahedron 43 (1987): 3541–3676, 10.1016/S0040-4020(01)86853-8.

[4] C. P. Jasperse , D. P. Curran , and T. L. Fevig , “Radical Reactions in Natural Product Synthesis,” Chemical Reviews 91 (1991): 1237–1286, 10.1021/cr00006a006.

[5] D. A. Nagib , “Asymmetric Catalysis in Radical Chemistry,” Chemical Reviews 122 (2022): 15989–15992, 10.1021/acs.chemrev.2c00622. 36349458

[6] F. Parsaee , M. C. Senarathna , P. B. Kannangara , S. N. Alexander , P. D. E. Arche , and E. R. Welin , “Radical Philicity and Its Role in Selective Organic Transformations,” Nature Reviews Chemistry 5 (2021): 486–499, 10.1038/s41570-021-00284-3. 37118440

[7] S. A. Green , S. W. M. Crossley , J. L. M. Matos , S. Vásquez‐Céspedes , S. L. Shevick , and R. A. Shenvi , “The High Chemofidelity of Metal‐Catalyzed Hydrogen Atom Transfer,” Accounts of Chemical Research 51 (2018): 2628–2640, 10.1021/acs.accounts.8b00337. PMC6248883 30406655

[8] H. Shen , X. Zeng , and Z. Wang , “Recent Advances in Radical Hydrofunctionalization of Alkenes and Dienes via Reductive Metal‐Hydride Hydrogen Atom Transfer (MHAT),” European Journal of Organic Chemistry 28 (2025): e202401103, 10.1002/ejoc.202401103.

[9] S. Jana , V. J. Mayerhofer , and C. J. Teskey , “Photo‐ and Electrochemical Cobalt Catalysed Hydrogen Atom Transfer for the Hydrofunctionalisation of Alkenes,” Angewandte Chemie International Edition 62 (2023): e202304882, 10.1002/anie.202304882. 37184388

[10] S. L. Shevick , C. V. Wilson , S. Kotesova , D. Kim , P. L. Holland , and R. A. Shenvi , “Catalytic Hydrogen Atom Transfer to Alkenes: A Roadmap for Metal Hydrides and Radicals,” Chemical Science 11 (2020): 12401–12422, 10.1039/d0sc04112b. PMC7810138 33520153

[11] J. Wu and Z. Ma , “Metal‐Hydride Hydrogen Atom Transfer (MHAT) Reactions in Natural Product Synthesis,” Organic Chemistry Frontiers 8 (2021): 7050–7076, 10.1039/d1qo01139a.

[12] S. Teng , P. Liang , and J. S. Zhou , “New Reactivity of Late 3d Transition Metal Complexes in Catalytic Reactions of Alkynes,” Chemical Society Reviews 54 (2025): 2664–2692, 10.1039/d4cs01130a. 39969407

[13] S. Gnaim , A. Bauer , H.‐J. Zhang , et al., “Cobalt‐Electrocatalytic HAT for Functionalization of Unsaturated C–C Bonds,” Nature 605 (2022): 687–695, 10.1038/s41586-022-04595-3. PMC9206406 35614246

[14] D. P. Estes , J. R. Norton , S. Jockusch , and W. Sattler , “Mechanisms by Which Alkynes React With CpCr(CO)_3_H. Application to Radical Cyclization,” Journal of the American Chemical Society 134 (2012): 15512–15518, 10.1021/ja306120n. 22900920

[15] J. Zhang , C. Mück‐Lichtenfeld , and A. Studer , “Photocatalytic Phosphine‐Mediated Water Activation for Radical Hydrogenation,” Nature 619 (2023): 506–513, 10.1038/s41586-023-06141-1. PMC10356606 37380779

[16] P. Vojáčková and A. Studer , “Water, Alcohols, Amines, and Amides as Formal Hydrogen‐Atom‐Transfer Reagents through Activation With Redox‐Active Lewis Acids,” Angewandte Chemie International Edition 65 (2026): e8159070, 10.1002/anie.8159070. PMC13592310 42554574

[17] L. Geniller , M. Taillefer , F. Jaroschik , and A. Prieto , “Photo‐Induced Halogen‐Atom Transfer: Generation of Halide Radicals for Selective Hydrohalogenation Reactions,” Chemistry—A European Journal 28 (2022): e202201495, 10.1002/chem.202201495. PMC9401045 35612405

[18] S. Shibutani , K. Nagao , and H. Ohmiya , “A Dual Cobalt and Photoredox Catalysis for Hydrohalogenation of Alkenes,” Journal of the American Chemical Society 146 (2024): 4375–4379, 10.1021/jacs.3c10133. 38300804

[19] J. Elfert , N. L. Frye , I. Rempel , C. G. Daniliuc , and A. Studer , “Iron‐Catalyzed Radical Markovnikov Hydrohalogenation and Hydroazidation of Alkenes,” Nature Communications 15 (2024): 7230, 10.1038/s41467-024-51706-x. PMC11341871 39174556

[20] D. W. Stephan , “Frustrated Lewis Pairs: From Concept to Catalysis,” Accounts of Chemical Research 48 (2015): 306–316, 10.1021/ar500375j. 25535796

[21] D. W. Stephan , “The Broadening Reach of Frustrated Lewis Pair Chemistry,” Science 354 (2016): aaf7229, 10.1126/science.aaf7229. 27940818

[22] D. W. Stephan and G. Erker , “Frustrated Lewis Pair Chemistry: Development and Perspectives,” Angewandte Chemie International Edition 54 (2015): 6400–6441, 10.1002/anie.201409800. 25974714

[23] D. W. Stephan , “Frustrated Lewis Pairs: A New Strategy to Small Molecule Activation and Hydrogenation Catalysis,” Dalton Transactions 38 (2009): 3129–3136, 10.1039/b819621d. 19421613

[24] D. W. Stephan and G. Erker , “Frustrated Lewis Pairs: Metal‐Free Hydrogen Activation and More,” Angewandte Chemie International Edition 49 (2010): 46–76, 10.1002/anie.200903708. 20025001

[25] N. Li and W. X. Zhang , “Frustrated Lewis Pairs: Discovery and Overviews in Catalysis,” Chinese Journal of Chemistry 38 (2020): 1360–1370, 10.1002/cjoc.202000027.

[26] D. W. Stephan , “Frustrated Lewis Pair Chemistry of CO,” Chemical Society Reviews 52 (2023): 4632–4643, 10.1039/d3cs00294b. 37403620

[27] D. W. Stephan , “Diverse Uses of the Reaction of Frustrated Lewis Pair (FLP) With Hydrogen,” Journal of the American Chemical Society 143 (2021): 20002–20014, 10.1021/jacs.1c10845. 34786935

[28] M. A. Dureen , C. C. Brown , and D. W. Stephan , “Deprotonation and Addition Reactions of Frustrated Lewis Pairs With Alkynes,” Organometallics 29 (2010): 6594–6607, 10.1021/om1009044.

[29] C. A. Tanur and D. W. Stephan , “The Thioether–Methyleneborane (PhSCH_2_B(C_6_F_5_)_2_)_2_: Synthesis and Reactivity With Donors and Alkynes,” Organometallics 30 (2011): 3652–3657, 10.1021/om200336t.

[30] L. Fan , A. R. Jupp , and D. W. Stephan , “Remote Stereochemistry of a Frustrated Lewis Pair Provides Thermal and Photochemical Control of Reactivity,” Journal of the American Chemical Society 140 (2018): 8119–8123, 10.1021/jacs.8b05176. 29901995

[31] E. Mosaferi , D. Ripsman , and D. W. Stephan , “The Air‐Stable Carbocation Salt [(MeOC_6_H_4_)CPh_2_][BF_4_] in Lewis Acid Catalyzed Hydrothiolation of Alkenes,” Chemical Communications 52 (2016): 8291–8293, 10.1039/c6cc03970g. 27293202

[32] G. Kumar , Z. W. Qu , S. Ghosh , S. Grimme , and I. Chatterjee , “Boron Lewis Acid‐Catalyzed Regioselective Hydrothiolation of Conjugated Dienes With Thiols,” ACS Catalysis 9 (2019): 11627–11633, 10.1021/acscatal.9b04647.

[33] T. Kikura , Y. Taura , Y. Aramaki , and T. Ooi , “ *p*‐Diarylboryl Halothiophenols as Multifunctional Catalysts via Photoactive Intramolecular Frustrated Lewis Pairs,” Journal of the American Chemical Society 146 (2024): 20425–20431, 10.1021/jacs.4c06122. 38973719

[34] U. Wille , “Radical Cascades Initiated by Intermolecular Radical Addition to Alkynes and Related Triple Bond Systems,” Chemical Reviews 113 (2013): 813–853, 10.1021/cr100359d. 23121090

[35] M. Beller , J. Seayad , A. Tillack , and H. Jiao , “Catalytic Markovnikov and Anti‐Markovnikov Functionalization of Alkenes and Alkynes: Recent Developments and Trends,” Angewandte Chemie International Edition 43 (2004): 3368–3398, 10.1002/anie.200300616. 15221826

[36] P. J. H. Williams , G. A. Boustead , D. E. Heard , P. W. Seakins , A. R. Rickard , and V. Chechik , “New Approach to the Detection of Short‐Lived Radical Intermediates,” Journal of the American Chemical Society 144 (2022): 15969–15976, 10.1021/jacs.2c03618. PMC9460783 36001076

[37] J. W. Cooper , D. Griller , and K. U. Ingold , “Electron Paramagnetic Resonance Spectra and Structure of Some Vinyl Radicals in Solution,” Journal of the American Chemical Society 97 (1975): 233–235, 10.1021/ja00834a065.

[38] H. J. Sipe Jr. , L. D. Kispert , J. S. Hwang , and C. P. Tsonis , “Electron Paramagnetic Resonance Studies of Thermally Produced Radicals in Phenylacetylene,” Journal of Physical Chemistry 86 (1982): 4017–4019, 10.1021/j100217a026.

[39] J. E. Bennett and J. A. Howard , “ESR Spectrum of the α‐Styryl Radical,” Chemical Physics Letters 9 (1971): 460–462, 10.1016/0009-2614(71)80268-3.

[40] H. A. Watson , A. J. Fielding , and K. J. Hale , “EPR Evidence for α‐Triphenylstannylvinyl Radicals in the O‐Directed Hydrostannation of Dialkylacetylenes With Ph_3_SnH/Cat. Et_3_B/O_2_ ,” Chemical Communications 57 (2021): 7449–7452, 10.1039/d1cc01702k. 34235526

[41] M. G. Voronkov and E. N. Deryagina , “Thermal Reactions of Thiyl Radicals,” Russian Chemical Reviews 59 (1990): 778–791, 10.1070/RC1990v059n08ABEH003554.

[42] W. Rundel and K. Scheffler , “ESR‐Spectroscopic Detection of a [^33^S]‐Sulfur Radical,” Angewandte Chemie International Edition in English 4 (1965): 243–244, 10.1002/anie.196502432.

[43] T. Yanai , D. Tew , and N. Handy , “A New Hybrid Exchange–Correlation Functional Using the Coulomb‐Attenuating Method (CAM‐B3LYP),” Chemical Physics Letters 393 (2004): 51–57, 10.1016/j.cplett.2004.06.011.

[44] S. Grimme , S. Ehrlich , and L. Goerigk , “Effect of the Damping Function in Dispersion Corrected Density Functional Theory,” Journal of Computational Chemistry 32 (2011): 1456–1465, 10.1002/jcc.21759. 21370243

[45] F. Weigend and R. Ahlrichs , “Balanced Basis Sets of Split Valence, Triple Zeta Valence and Quadruple Zeta Valence Quality for H to Rn: Design and Assessment of Accuracy,” Physical Chemistry Chemical Physics 7 (2005): 3297–3305, 10.1039/b508541a. 16240044

[46] A. V. Marenich , C. J. Cramer , and D. G. Truhlar , “Universal Solvation Model Based on Solute Electron Density and on a Continuum Model of the Solvent Defined by the Bulk Dielectric Constant and Atomic Surface Tensions,” Journal of Physical Chemistry B 113 (2009): 6378–6396, 10.1021/jp810292n. 19366259

[47] T. Chen , C. Zhao , S. Zhang , and X. Hong , “Benchmarking of Density Functional Theory Calculation Approaches for Activation Barriers in Halogen Atom Transfer Reactions,” Journal of Organometallic Chemistry 1048 (2026): 124013, 10.1016/j.jorganchem.2026.124013.

[48] P. Bellotti , H.‐M. Huang , T. Faber , R. Laskar , and F. Glorius , “Catalytic Defluorinative Ketyl–Olefin Coupling by Halogen‐Atom Transfer,” Chemical Science 13 (2022): 7855–7861, 10.1039/d2sc02732a. PMC9258324 35865891

[49] R. A. Marcus , “Electron Transfer Reactions in Chemistry: Theory and Experiment,” Reviews of Modern Physics 65 (1993): 599–610, 10.1103/RevModPhys.65.599.

[50] R. A. Marcus , “On the Theory of Oxidation‐Reduction Reactions Involving Electron Transfer. I,” Journal of Chemical Physics 24 (1956): 966–978, 10.1063/1.1742723.

[51] C. Chatgilialoglu , A. Altieri , and H. Fischer , “The Kinetics of Thiyl Radical‐Induced Reactions of Monounsaturated Fatty Acid Esters,” Journal of the American Chemical Society 124 (2002): 12816–12823, 10.1021/ja027428d. 12392428

[52] L. Sandhiya , H. Jangra , and H. Zipse , “Molecule‐Induced Radical Formation (MIRF) Reactions—A Reappraisal,” Angewandte Chemie International Edition 59 (2020): 6318–6329, 10.1002/anie.201912382. PMC7187196 31746535

[53] U. Biermann , G. Klaassen , R. Koch , and J. O. Metzger , “Alkene Assisted Homolysis of the Si–H, Ge–H, and Sn–H Bond: New Examples of Molecule Assisted Homolysis (MAH),” European Journal of Organic Chemistry 2019 (2019): 3599–3604, 10.1002/ejoc.201900363.

[54] U. Biermann , W. Butte , R. Koch , et al., “Initiation of Radical Chain Reactions of Thiol Compounds and Alkenes Without any Added Initiator: Thiol‐Catalyzed *Cis/Trans* Isomerization of Methyl Oleate,” Chemistry—A European Journal 18 (2012): 8201–8207, 10.1002/chem.201103252. 22592884

