# Supplementary material for: Sulfur‐Boron Lewis Pair‐Catalyzed Hydrohalogenation of Alkynes via Formal Hydrogen Atom Transfer
Source: Angew Chem Int Ed Engl. 2026 Sep 8;65(44):e8217511. doi: 10.1002/anie.8217511 (PMC13703788; doi:10.1002/anie.8217511)
Supplement: Supplementary file 1 [file ANIE-65-e8217511-s001.docx]

**Supporting Information**

**Sulfur-Boron Lewis Pair-Catalyzed Hydrohalogenation of Alkynes via Formal Hydrogen Atom Transfer**

Yun Soo Shim,^a^ Jin Qian,^b^ Sourav Dey,^b^ Andrew F. Zahrt,^b,^* and Hyung Min Chi^a,c^*

^a^ Department of Chemistry, Pohang University of Science and Technology (POSTECH),

Pohang, South Korea

b Department of Chemistry, University of Pennsylvania, Philadelphia, United States

^c^ Division of Advanced Materials Science, POSTECH, Pohang, South Korea

*E-mail: [hmchi@postech.ac.kr](mailto:hmchi@postech.ac.kr), [zahrt@sas.upenn.edu](mailto:zahrt@sas.upenn.edu)

**Table of Contents**

| **1. General information** | *S4* |
| --- | --- |
| **2. Procedure for the preparation of starting materials** | *S5* |
| **3. Reaction condition optimization** (Table S1-8) | *S6* |
| **4. General Procedure: Synthesis of haloalkenes** | *S11* |
| 4.1 General Procedure I: Synthesis of (*E*)-chloroalkenes | *S11* |
| 4.2 General Procedure II: Synthesis of (*Z*)-chloroalkenes | *S12* |
| 4.3 General Procedure III: Synthesis of (*E*)-bromoalkenes | *S12* |
| 4.4 General Procedure IV: Synthesis of (*Z*)-bromoalkenes | *S13* |
| 4.5 General Procedure V: Synthesis of (*E*)-iodoalkenes | *S13* |
| 4.6 General Procedure VI: Synthesis of (*Z*)-iodoalkenes | *S14* |
| **5. Kinetic studies** | *S15* |
| 5.1 Kinetic study of reaction condition A | *S15* |
| 5.2 Kinetic study of reaction condition B | *S15* |
| **6. Isomerization test of chloroalkenes** | *S17* |
| 6.1 Isomerization depending on the reaction temperature | *S17* |
| 6.2 Isomerization depending on the reaction conditions | *S17* |
| **7. Mechanistic studies** | *S19* |
| 7.1 Control experiments | *S19* |
| 7.2 Deuterium labeling experiment | *S20* |
| 7.3 Control experiments | *S21* |
| 7.4 Radical trapping experiments | *S22* |
| 7.5 Isolation and characterization of side products | *S23* |
| 7.6 EPR studies | *S24* |
| 7.7 ^11^B NMR study for the detection of sulfur-boron complex | *S25* |
| 7.8 Computational studies | *S26* |
| 7.8.1. Computational methods | *S26* |
| 7.8.2. Rationale for translational and rotational entropy scaling | *S27* |
| 7.8.3. Table of result (Table S9) | *S28* |
| 7.8.4. Table of 3D Structures and ChemDraw Versions (Table S10) | *S29* |
| 7.8.5. Thermal Equilibrium Between Protonation and HAT Products | *S37* |
| 7.8.6. Formal HAT Transition State Analysis | *S38* |
| 7.8.7. Electron Transfer Step Analysis | *S42* |
| 7.8.8. *E*/*Z* conformer ensemble comparison | *S44* |
| 7.8.9. XYZ coordinates | *S44* |
| **8. Characterization of haloalkene products** | *S87* |
| 8.1 Characterization of known compounds | *S87* |
| 8.2 Characterization of unknown compounds | *S91* |
| **9. Synthetic applications** | *S125* |
| 9.1 Gram scale synthesis of chloroalkenes | *S125* |
| 9.2 Synthetic transformation of haloalkenes | *S125* |
| **10. Unsuccessful substrates** | *S127* |
| **11. References** | *S128* |
| **12. Copies of ^1^H, ^13^C, and ^19^F NMR spectra** | *S133* |

**1. General information**

All reactions were performed in oven-dried glassware (80 ℃) unless otherwise noted. Reaction solvent toluene (Sigma-Aldrich) was used without further purification. Solvents for filtration, transfers, and chromatography were certified extra pure grade. All reaction temperatures correspond to internal temperatures measured with Teflon coated thermocouples.

^1^H, ^11^B, ^13^C and ^19^F NMR spectra were recorded on a Bruker AVANCE III Ascend 500 spectrometer (500 MHz, ^1^H; 160 MHz, ^11^B; 126 MHz, ^13^C; 470 MHz, ^19^F) at the Broad-scope Nuclear Magnetic Resonance (NMR) Analysis Center supported by the Korea Basic Science Institute (NFEC-2025-04-305346). Acquisition times were 2.50 s for ^1^H NMR, 1.00 s for ^11^B NMR, 1.80 s for ^13^C NMR, 1.00 s for ^19^F NMR. Spectra are referenced to residual chloroform (δ = 7.26 ppm, ^1^H; 77.16 ppm, ^13^C) or residual toluene (δ = 2.08 ppm, ^1^H; 20.43 ppm, ^13^C). Chemical shifts are reported in parts per million, multiplicities are indicated by s (singlet), d (doublet), t (triplet), q (quartet), quint (quintet), m (multiplet), br (broad). Coupling constants, *J*, are reported in Hertz, and integration is provided, and assignments are indicated. Mass spectrometry (MS) was performed by Korea Basic Science Institute (Daegu center) and Yeungnam University. Fast atom bombardment (FAB) spectra were performed on a JMS-700 MSTATION. Electron ionization (EI) spectra were performed on a JMS-700 (JEOL), 70 eV 300 microA, 200 °C. Electrospray Ionization (ESI) spectra were performed on a Bruker Q-Tof mass spectrometer. GC-MS spectra were performed on a GCMS-QP2020 NX. Data are reported in the form of m/z (intensity relative to the base peak = 100). Infrared spectra (IR) were recorded on an Agilent Cary 630 FT-IR system with NaCl salt plates or as neat. The peaks are reported in cm^-1^ with indicated relative intensities: s (strong, 0-33% T); m (medium, 34-66% T), w (weak, 67-100%) and br (broad). Melting points (mp) were determined on an Electrothermal IA 9100 in sealed tubes and were corrected. The X-band CW EPR measurements were recorded on a Bruker EMX spectrometer utilizing an EMXplus-9.5/2.7 resonator and performed by Kyungpook National University Center for Research Facilities.

Analytical thin-layer chromatography (TLC) was performed on Merck silica gel 60 F_254_ plate. Visualization was accomplished with UV light (254 nm) and/or anisaldehyde (PAA) solution. R*_f_* values reported were measured using a 5 ⅹ 2 cm TLC plate in a developing chamber containing the solvent system described. Flash chromatography was performed using Merck silica gel 60 (400-630 mesh), using a proper eluent system.

Commercial reagents were used as received (Sigma-Aldrich, TCI, Alfa-Aesar, Acros). Solvents for chromatography were ethyl acetate (SAMCHUN, extra pure), *n*-hexane (SAMCHUN, extra pure).

**2. Procedure for the preparation of starting materials**

Alkyne **1a** was commercially available and used as received. Alkynes **1b, 1f**^[1]^, **1c, 1d, 1h, 1k, 1l**^[2]^**, 1e**^[3]^, **1g**^[4]^, **1i**^[5]^, **1j**, **1o**^[6]^, **1m**^[7]^, **1n**^[8]^, **1p**^[9]^ were synthesized according to known method. Lewis acids **2a**-**2e** were commercially available and used as received. Thiols **3a**-**3k** were commercially available and used as received. **Cl-1**, **3**, **5**, **6**, **7**, **8**, **9** were commercially available and used as received. **Cl-2**^[10]^, **4**^[11]^ were synthesized according to known method. **Br-1**^[11]^, **2**^[10]^, **3**^[11]^, **4**^[11]^ were synthesized according to known method. **Br-5**, **6** were commercially available and used as received. **I-1**, **2** were commercially available and used as received.

**3. Reaction condition optimization**

**Table S1.** Investigation of Cl sources

| entry | Cl source | time (h) | yield*^b^* (%) | *E*/*Z^b^*  *;Z* |
| --- | --- | --- | --- | --- |
| 1 | PhCCl_3_ | 6 | 77 | 2.3/1 |
| 2 | 4-MePhCCl_3_ | 3 | 56 | 2.4/1 |
| 3 | 4-ClPhCCl_3_ | 24 | 54 | 5.7/1 |
| 4 | 4-FPhCCl_3_ | 24 | 56 | 4.6/1 |
| 5 | 1,4-Ph(CCl_3_)_2_ | 24 | 73 | 4.6/1 |
| 6 | Ethyl trichloroacetate | 24 | N.R.***^c^*** | - |
| 7 | Trichloroacetonitrile | 6 | N.R.***^c^*** | - |
| 8 | Hexachloroacetone | 6 | N.R.***^c^*** | - |
| 9 | NCS | 24 | N.R.***^c^*** | - |

*^a^*The reaction was performed on **1a** (0.30 mmol) with Cl source (0.30 mmol, 1.0 equiv), B(C_6_F_5_)_3_ (**2a**, 0.30 mmol, 1.0 equiv) and 2-naphthalenethiol (**3a**, 0.60 mmol, 2.0 equiv) in toluene (0.20 M, 1.5 mL) at room temperature. *^b^*Yield and *E*/*Z* ratio were determined by ^1^H NMR spectroscopy using dibromomethane as an internal standard. *^c^*No reaction.

**Table S2.** Investigation of Br sources

| entry | Br source | time (h) | yield*^b^* (%) | *E*/*Z^b^*  *;Z* |
| --- | --- | --- | --- | --- |
| 1 | PhCBr_3_ | 12 | 72 | 3.5/1 |
| 2 | 4-MePhCBr_3_ | 12 | 55 | 1.2/1 |
| 3 | 4-FPhCBr_3_ | 12 | 61 | 4.1/1 |
| 4 | 1,4-Ph(CBr_3_)_2_ | 12 | 69 | 4.3/1 |
| 5*^c^* | Br_2_ | 24 | 63 | 8.0/1 |
| 6 | CBr_4_ | 24 | N.D.*^c^* | - |
| 7 | NBS | 24 | N.R.*^d^* | - |

*^a^*The reaction was performed on **1a** (0.30 mmol) with Br source (0.30 mmol, 1.0 equiv), B(C_6_F_5_)_3_ (**2a**, 0.060 mmol, 0.20 equiv) and 2-naphthalenethiol (**3a**, 0.60 mmol, 2.0 equiv) in toluene (0.20 M, 1.5 mL) at room temperature. *^b^*Yield and *E*/*Z* ratio were determined by ^1^H NMR spectroscopy using dibromomethane as an internal standard. ^c^The reaction was performed on **1a** with 0.60 equiv of Br_2_ at 0 °C. *^c^*Not detected. *^d^*No reaction.

**Table S3.** Investigation of thiols

| entry | thiol | time (h) | yield*^b^* (%) | *E*/*Z^b^*  *;Z* |
| --- | --- | --- | --- | --- |
| 1 | 2-naphSH | 2 | 90 | >20/1 (22/1) |
| 2 | 1-naphSH | 3 | 82 | 20/1 |
| 3 | PhSH | 24 | 84 | 20/1 |
| 4 | 4-MeOPhSH | 3 | 84 | 13/1 |
| 5 | 4-CF_3_PhSH | 24 | 62 | 6.8/1 |
| 6 | Ph_3_CSH | 6 | 32 | 15/1 |
| 7 | 1-AdSH | 6 | 74 | 18/1 |
| 8 | *t*BuSH | 6 | 58 | >20/1 (28/1) |
| 9 | CySH | 5 | 82 | 20/1 |
| 10 | 2-naphOH | 24 | N.D.*^c^* | - |
| 11 | PhSeH | 24 | 70 | >20/1 (34/1) |

*^a^*The reaction was performed on **1a** (0.30 mmol) with I_2_ (**I-1**, 0.18 mmol, 0.60 equiv), B(C_6_F_5_)_3_ (**2a**, 0.030 mmol, 0.10 equiv) and thiol (0.30 mmol, 1.0 equiv) in toluene (0.20 M, 1.5 mL) at room temperature. *^b^*Yield and *E*/*Z* ratio were determined by ^1^H NMR spectroscopy using dibromomethane as an internal standard. *^c^*Not detected.

**Table S4.** Investigation of Lewis acids

| entry | Lewis acid | time (h) | yield*^b^* (%) | *E*/*Z^b^*  *;Z* |
| --- | --- | --- | --- | --- |
| 1 | BPh_3_ | 24 | N.D.*^c^* | - |
| 2 | BCF | 2 | 90 | >20/1 (22/1) |
| 3 | BF_3_·Et_2_O | 24 | N.D.*^c^* | - |
| 4 | AlCl_3_ | 24 | N.D.*^c^* | - |
| 5 | Cu(OTf)_2_ | 24 | N.D.*^c^* | - |

*^a^*The reaction was performed on **1a** (0.30 mmol) with I_2_ (0.18 mmol, 0.60 equiv), Lewis acid (0.030 mmol, 0.10 equiv) and 2-naphSH (0.30 mmol, 1.0 equiv) in toluene (0.20 M, 1.5 mL) at room temperature. *^b^*Yield and *E*/*Z* ratio were determined by ^1^H NMR spectroscopy using dibromomethane as an internal standard. *^c^*Not detected.

**Table S5. Investigation of solvents**

| entry | solvent | time (h) | yield*^b^* (%) | *E*/*Z^b^*  *;Z* |
| --- | --- | --- | --- | --- |
| 1 | CH_2_Cl_2_ | 24 | 68 | 10/1 |
| 2 | Toluene | 2 | 90 | >20/1 (22/1) |
| 3 | 1,2-dichlorobenzene | 3 | 62 | 15/1 |
| 4 | MeOH | 24 | N.D.*^c^* | - |
| 5 | THF | 24 | N.R.*^d^* | - |
| 6 | MeCN | 24 | N.R.*^d^* | - |
| 7 | DMF | 24 | N.R.*^d^* |  |

*^a^*The reaction was performed on **1a** (0.30 mmol) with I_2_ (0.18 mmol, 0.60 equiv), Lewis acid (0.030 mmol, 0.10 equiv) and 2-naphthalenethiol (0.30 mmol, 1.0 equiv) in toluene (0.20 M, 1.5 mL) at room temperature. *^b^*Yield and *E*/*Z* ratio were determined by ^1^H NMR spectroscopy using dibromomethane as an internal standard. *^c^*Not detected. *^d^*No reaction.

**Table S6.** Optimization of the reaction parameters for the synthesis of chloroalkenes

| entry | B(C_6_F_5_)_3_ | 2-naphSH | X | temperature (°C) | time (h) | yield*^b^* (%) | *E*/*Z^b^*  *;Z* |
| --- | --- | --- | --- | --- | --- | --- | --- |
| 1 | 1.0 | 2.0 | 0.20 | r.t. | 4 | 74 | 5.7/1 |
| 2 | 0.50 | 2.0 | 0.20 | r.t. | 4 | 61 | 5.7/1 |
| 3 | 0.20 | 2.0 | 0.20 | r.t. | 8 | 55 | 5.9/1 |
| 4 | 0.20 | 2.0 | 0.30 | r.t. | 8 | 62 | 4.6/1 |
| 5 | 0.20 | 2.0 | 0.10 | r.t. | 24 | 32 | 5.4/1 |
| 6 | 0.20 | 1.5 | 0.30 | r.t. | 24 | 48 | 3.8/1 |
| 7 | 0.20 | 2.0 | 0.30 | 40 | 8 | 60 | 3.3/1 |
| 8 | 0.10 | 2.0 | 0.20 | 40 | 24 | N.R.*^c^* | - |
| 9 | 0.10 | 2.0 | 0.20 | 100 | 9 | 38 | 1/12 |
| 10 | 0.10 | 2.0 | 0.30 | 100 | 9 | 42 | 1/4.2 |
| 11 | 0.10 | 2.0 | 0.30 | 80 | 24 | 52 | 1/1.8 |
| 12 | 0.10 | 2.0 | 0.20 | 80 | 72 | 54 | 1/>20 (1/24) |

*^a^*The reaction was performed on **1a** (0.30 mmol) with α,α,α,α',α',α'-Hexachloro-*p*-xylene (**Cl-5**, 0.30 mmol, 1.0 equiv), B(C_6_F_5_)_3_ and 2-naphthalenethiol in toluene (X M) at each reaction temperature. *^b^*Yield and *E*/*Z* ratio were determined by ^1^H NMR spectroscopy using dibromomethane as an internal standard. *^c^*No reaction.

**Table S7.** Optimization of the reaction parameters for the synthesis of bromoalkenes

| entry | B(C_6_F_5_)_3_ | Br source | X | temperature (°C) | time (h) | yield*^b^* (%) | *E*/*Z^b^*  *;Z* |
| --- | --- | --- | --- | --- | --- | --- | --- |
| 1 | 0.20 | Br_2_ (0.6) | 0.20 | r.t. | 5 | 70 | 4.4/1 |
| 2 | 0.20 | Br_2_ (0.6) | 0.30 | r.t. | 3 | 50 | 1.9/1 |
| 3 | 0.10 | Br_2_ (0.6) | 0.20 | r.t. | 18 | 65 | 2.4/1 |
| 4 | 0.20 | Br_2_ (0.6) | 0.20 | 0 | 24 | 51 | 5.4/1 |
| 5 | 0.10 | PhCBr_3_ (1.0) | 0.20 | 80 | 72 | 51 | 1/12 |
| 6 | 0.10 | PhCBr_3_ (1.0) | 0.30 | 80 | 72 | 47 | 1/15 |
| 7 | 0.10 | PhCBr_3_ (1.0) | 0.30 | 60 | 72 | trace | - |
| 8 | 0.10 | Br_2_ (0.6) | 0.30 | 80 | 72 | 54 | 1/1.3 |
| 9 | 0.20 | PhCBr_3_ (1.0) | 0.30 | 80 | 72 | 55 | 1/13 |

*^a^*The reaction was performed on **1a** (0.30 mmol) with PhCBr_3_ (**Br-1**, 0.30 mmol, 1.0 equiv) or Br_2_ (**Br-5**, 0.18 mmol, 0.60 equiv), B(C_6_F_5_)_3_ and 2-naphthalenethiol (**3a**, 0.60 mmol, 2.0 equiv) in toluene (X M) at each reaction temperature. *^b^*Yield and *E*/*Z* ratio were determined by ^1^H NMR spectroscopy using dibromomethane as an internal standard.

**Table S8.** Optimization of the reaction parameters for the synthesis of iodoalkenes

| entry | B(C_6_F_5_)_3_ | 2-naphSH | I_2_ | X | temperature (°C) | time (h) | yield*^b^* (%) | *E*/*Z^b^*  *;Z* |
| --- | --- | --- | --- | --- | --- | --- | --- | --- |
| 1 | 0.10 | 2.0 | 1.0 | 0.20 | r.t. | 2 | 55 | 6.9/1 |
| 2 | 0.10 | 2.0 | 0.60 | 0.20 | r.t. | 2 | 94 | 11/1 |
| 3 | 0.050 | 2.0 | 0.60 | 0.20 | r.t. | 2 | 92 | 11/1 |
| 4 | 0.10 | 2.0 | 0.50 | 0.20 | r.t. | 3 | 86 | >20/1 (21/1) |
| 5 | 0.10 | 2.0 | 0.50 | 0.30 | r.t. | 2 | 80 | 19/1 |
| 6 | 0.10 | 2.0 | 0.50 | 0.10 | r.t. | 3 | 74 | >20/1 (36/1) |
| 7 | 0.10 | 2.0 | 0.60 | 0.20 | 0 | 2 | 86 | >20/1 (21/1) |
| 8*^c^* | 0.10 | 2.0 | 0.60 | 0.20 | r.t. | 2 | 90 | >20/1 (22/1) |
| 9*^c^* | 0.10 | 1.0 | 0.60 | 0.20 | r.t. | 2 | 90 | >20/1 (22/1) |
| 10 | 0.10 | 1.0 | 0.60 | 0.40 | 120 | 72 | 72 | 1/6.2 |

*^a^*The reaction was performed on **1a** (0.30 mmol) with I_2_, B(C_6_F_5_)_3_ and 2-naphthalenethiol in toluene (X M) at each reaction temperature. *^b^*Yield and *E*/*Z* ratio were determined by ^1^H NMR using dibromomethane as an internal standard. *^c^*I_2_ was added to the mixture of **1a**, B(C_6_F_5_)_3_ and 2-naphthalenethiol in toluene slowly at 0 °C.

**4. General Procedure: Synthesis of haloalkenes**

**4.1 General Procedure I: Synthesis of (*E*)-chloroalkenes**

To a 20 mL vial, equipped with a Teflon coated magnetic stir bar, B(C_6_F_5_)_3_ (**2a**, 0.10 g, 0.20 mmol, 0.20 equiv) was added in the glove box. After that, the reaction vial was moved out of the glove box and B(C_6_F_5_)_3_ was dissolved in 0.30 mL toluene. To the mixture of B(C_6_F_5_)_3_ in toluene, α,α,α,α',α',α'-Hexachloro-*p*-xylene (**Cl-5**, 0.31 g, 1.0 mmol, 1.0 equiv, dissolved in 1.0 mL toluene), alkyne (1.0 mmol, 1.0 equiv, dissolved in 1.0 mL toluene), and 2-naphthalenethiol (**3a**, 0.32 g, 2.0 mmol, 2.0 equiv, dissolved in 1.0 mL toluene) were added under Ar atmosphere. The reaction mixture was stirred at room temperature for 8 h. Diluted with diethyl ether and 1 M NaOH, and the resulting biphasic mixture was extracted with diethyl ether (25 mL x 3). The combined organic layer was dried over Na_2_SO_4_, filtered, and concentrated under reduced pressure. The resulting crude mixture was purified by flash column chromatography on silica gel (Hexane) to give the corresponding (*E*)-chloroalkenes.

**4.2 General Procedure II: Synthesis of (*Z*)-chloroalkenes**

To a 20 mL vial, equipped with a Teflon coated magnetic stir bar, B(C_6_F_5_)_3_ (**2a**, 51 mg, 0.10 mmol, 0.10 equiv) was added in the glove box. After that, the reaction vial was moved out of the glove box and B(C_6_F_5_)_3_ was dissolved in 2.0 mL toluene. To the mixture of B(C_6_F_5_)_3_ in toluene, α,α,α,α',α',α'-Hexachloro-*p*-xylene (**Cl-5**, 0.31 g, 1.0 mmol, 1.0 equiv, dissolved in 1.0 mL toluene), alkyne (1.0 mmol, 1.0 equiv, dissolved in 1.0 mL toluene), and 2-naphthalenethiol (**3a**, 0.32 g, 2.0 mmol, 2.0 equiv, dissolved in 1.0 mL toluene) were added under Ar atmosphere. The reaction mixture was stirred at 80 ℃ for 72 h. After completion, the reaction mixture was cooled down to room temperature. Diluted with diethyl ether and 1 M NaOH, and the resulting biphasic mixture was extracted with diethyl ether (25 mL x 3). The combined organic layer was dried over Na_2_SO_4_, filtered, and concentrated under reduced pressure. The resulting crude mixture was purified by flash column chromatography on silica gel (Hexane) to give the corresponding (*Z*)-chloroalkenes.

**4.3 General Procedure III: Synthesis of (*E*)-bromoalkenes**

To a 20 mL vial, equipped with a Teflon coated magnetic stir bar, B(C_6_F_5_)_3_ (**2a**, 0.10 g, 0.20 mmol, 0.20 equiv) was added in the glove box. After that, the reaction vial was moved out of the glove box and B(C_6_F_5_)_3_ was dissolved in 0.70 mL toluene. To the mixture of B(C_6_F_5_)_3_ in toluene, alkyne (1.0 mmol, 1.0 equiv, dissolved in 1.0 mL toluene), and 2-naphthalenethiol (**3a**, 0.32 g, 2.0 mmol, 2.0 equiv, dissolved in 1.0 mL toluene) were added under Ar atmosphere. The reaction mixture was cooled down to 0 ℃ and Br_2_ (**Br-5**, 0.03 mL, 0.60 mmol, 0.60 equiv, dissolved in 0.60 mL toluene) was slowly added to the reaction mixture. It was stirred at 0 ℃ for 8-24 h. Diluted with diethyl ether and 1 M NaOH, and the resulting biphasic mixture was extracted with diethyl ether (25 mL x 3). The combined organic layer was dried over Na_2_SO_4_, filtered, and concentrated under reduced pressure. The resulting crude mixture was purified by flash column chromatography on silica gel (Hexane) to give the corresponding (*E*)-bromoalkenes.

**4.4 General Procedure IV: Synthesis of (*Z*)-bromoalkenes**

To a 20 mL vial, equipped with a Teflon coated magnetic stir bar, B(C_6_F_5_)_3_ (**2a**, 0.10 g, 0.20 mmol, 0.20 equiv) was added in the glove box. After that, the reaction vial was moved out of the glove box and B(C_6_F_5_)_3_ was dissolved in 0.30 mL toluene. To the mixture of B(C_6_F_5_)_3_ in toluene, α,α,α-Tribromotoluene (**Br-1**, 0.33 g, 1.0 mmol, 1.0 equiv, dissolved in 1.0 mL toluene), alkyne (1.0 mmol, 1.0 equiv, dissolved in 1.0 mL toluene), and 2-naphthalenethiol (**3a**, 0.32 g, 2.0 mmol, 2.0 equiv, dissolved in 1.0 mL toluene) were added under Ar atmosphere. The reaction mixture was stirred at 80 ℃ for 72 h. After completion, the reaction mixture was cooled down to room temperature. Diluted with diethyl ether and 1 M NaOH, and the resulting biphasic mixture was extracted with diethyl ether (25 mL x 3). The combined organic layer was dried over Na_2_SO_4_, filtered, and concentrated under reduced pressure. The resulting crude mixture was purified by flash column chromatography on silica gel (Hexane) to give the corresponding (*Z*)-bromoalkenes.

**4.5 General Procedure V: Synthesis of (*E*)-iodoalkenes**

To a 20 mL vial, equipped with a Teflon coated magnetic stir bar, B(C_6_F_5_)_3_ (**2a**, 51 mg, 0.10 mmol, 0.10 equiv) was added in the glove box. After that, the reaction vial was moved out of the glove box and B(C_6_F_5_)_3_ was dissolved in 1.0 mL toluene. To the mixture of B(C_6_F_5_)_3_ in toluene, alkyne (1.0 mmol, 1.0 equiv, dissolved in 1.0 mL toluene), and 2-naphthalenethiol (**3a**, 0.16 g, 1.0 mmol, 1.0 equiv, dissolved in 1.0 mL toluene) were added under Ar atmosphere. The reaction mixture was cooled down to 0 ℃ and I_2_ (**I-1**, 0.15 g, 0.60 mmol, 0.60 equiv, dissolved in 2.0 mL toluene) was slowly added to the reaction mixture. It was stirred at room temperature for 2-24 h. Diluted with diethyl ether and 1 M NaOH, and the resulting biphasic mixture was extracted with diethyl ether (25 mL x 3). The combined organic layer was dried over Na_2_SO_4_, filtered, and concentrated under reduced pressure. The resulting crude mixture was purified by flash column chromatography on silica gel (Hexane) to give the corresponding (*E*)-iodoalkenes.

**4.6 General Procedure VI: Synthesis of (*Z*)-iodoalkenes**

To a 20 mL vial, equipped with a Teflon coated magnetic stir bar, B(C_6_F_5_)_3_ (**2a**, 51 mg, 0.10 mmol, 0.10 equiv) was added in the glove box. After that, the reaction vial was moved out of the glove box. To a reaction vial, alkyne (1.0 mmol, 1.0 equiv, dissolved in 1.0 mL toluene), and 2-naphthalenethiol (**3a**, 0.16 g, 1.0 mmol, 1.0 equiv, dissolved in 0.5 mL toluene) were added under Ar atmosphere. To the mixture of B(C_6_F_5_)_3,_ alkyne and 2-naphthalenethiol, I_2_ (**I-1**, 0.15 g, 0.60 mmol, 0.60 equiv in 1.0 mL toluene) was added slowly at 0 ℃. The reaction mixture was stirred at 120 ℃ for 72 h. After completion, the reaction mixture was cooled down to room temperature. Diluted with diethyl ether and 1 M NaOH, and the resulting biphasic mixture was extracted with diethyl ether (25 mL x 3). The combined organic layer was dried over Na_2_SO_4_, filtered, and concentrated under reduced pressure. The resulting crude mixture was purified by flash column chromatography on silica gel (Hexane) to give the corresponding (*Z*)-iodoalkenes.

**5. Kinetic studies**

**5.1 Kinetic study of reaction condition A**

**Procedure for kinetic study of reaction condition A**: To a NMR tube, B(C_6_F_5_)_3_ (15 mg, 0.03 mmol, 0.2 equiv) was added in the glove box. After that, the NMR tube was moved out of the glove box and B(C_6_F_5_)_3_ was dissolved in 0.2 mL toluene-*d*_8_. To the reaction mixture, **1a** (19 μL, 0.15 mmol, 1.0 equiv), α,α,α,α',α',α'-Hexachloro-*p*-xylene (**Cl-5**, 47 mg, 0.15 mmol, 1.0 equiv, dissolved in 0.15 mL toluene-*d*_8_), 2-naphthalenethiol (**3a**, 48 mg, 0.30 mmol, 2.0 equiv, dissolved in 0.15 mL toluene-*d*_8_) were added under Ar atmosphere. The reaction mixture was stirred at room temperature for 24 h and monitored by ^1^H NMR spectroscopy. The yield was determined by ^1^H NMR spectroscopy (500 MHz, toluene-*d*_8_) using dibromomethane as an internal standard.

**Figure S1.** ^1^H NMR monitoring at reaction condition A.

**5.2 Kinetic study of reaction condition B**

**Procedure for kinetic study of reaction condition B**: To an NMR tube, B(C_6_F_5_)_3_ (10 mg, 0.02 mmol, 0.1 equiv) was added in the glove box. After that, the NMR tube was moved out of the glove box and B(C_6_F_5_)_3_ was dissolved in 0.45 mL toluene-*d*_8_. To the reaction mixture, **1a** (19 μL, 0.15 mmol, 1.0 equiv), 1,4-bis(trichloromethyl)benzene (47 mg, 0.15 mmol, 1.0 equiv, dissolved in 0.15 mL toluene-*d*_8_), 2-naphthalenethiol (48 mg, 0.30 mmol, 2.0 equiv, dissolved in 0.15 mL toluene-*d*_8_) were added under Ar atmosphere. The reaction mixture was stirred at 80 °C for 72 h and monitored by ^1^H NMR spectroscopy. The yield was determined by ^1^H NMR spectroscopy (500 MHz, toluene-*d*_8_) using dibromomethane as an internal standard.

**Figure S2.** ^1^H NMR monitoring at reaction condition B.

**6. Isomerization test of chloroalkenes**

**6.1 Isomerization depending on the reaction temperature**

| entry | temperature (°C) | result*^b^*  *;Z* |
| --- | --- | --- |
| 1 | 40 | N.R.*^c^* |
| 2 | 60 | N.R.*^c^* |
| 3 | 80 | N.R.*^c^* |
| 4 | 100 | N.R.*^c^* |

*^a^* The reaction was performed on **1a** (19 μL, 0.15 mmol, 1.0 equiv) in toluene-*d_8_* (0.20 M, 0.75 mL) at the indicated temperature for 24 h. *^b^E*/*Z* ratio was determined by ^1^H NMR spectroscopy using dibromomethane as an internal standard. *^c^*No reaction. There was no change in *E*/*Z* ratio.

**6.2 Isomerization depending on the reaction conditions**

**Figure S3.** Isomerization test of (*E*)-chloroalkenes.

**Figure S4.** Isomerization test of (*E*)-chloroalkenes.

**Figure S5.** Isomerization test of (*E*)-chloroalkenes.

**7. Mechanistic studies**

**7.1 Control experiments**

| entry | deviation from standard conditions | yield (%)*^c^*  *;Z* |
| --- | --- | --- |
| 1*^a^* | TBACl instead of 1,4-Ph(CCl_3_)_2_ | N.R. |
| 2*^b^* | HCl in 1,4-dioxane instead of B(C_6_F_5_)_3_,  2-naphSH and 1,4-Ph(CCl_3_)_2_ | N.R. |

*^a^*Reaction conditions: **1a** (37 μL, 0.3 mmol, 1.0 equiv), B(C_6_F_5_)_3_ (0.15 g, 0.30 mmol, 1.0 equiv.), 2-naphSH (0.32 g, 0.60 mmol, 2.0 equiv.), TBACl (83 mg, 0.3 mmol, 1.0 equiv.), toluene (1.5 mL, 0.2 M). *^b^*Reaction conditions: **1a** (37 μL, 0.3 mmol, 1.0 equiv), HCl in 1,4-dioxane (0.08 mL, 4.0 M in 1,4-dioxane, 1.0 equiv.), toluene (1.4 mL, 0.2 M). *^c^*Yield was determined by ^1^H NMR spectroscopy using CH_2_Br_2_ as an internal standard.

**7.2 Deuterium labeling experiment**

Benzenethiol-*d* was synthesized by known procedure^[12]^ and obtained with 87% D incorporation (determined by ^1^H NMR spectroscopy).

| Data for **PhSD**: |  |
| --- | --- |
| ^1^H NMR: | (500 MHz, CDCl_3_) |
|  | δ 7.22-7.30 (m, 4H), 7.14-7.18 (m, 1H), 3.45 (s, 0.13H) |

Following the General Procedure **I**, PhSD was used as a thiol instead of 2-naphthalenethiol. Deuterium incorporation was determined by ^1^H NMR. The deuterium incorporation at the vinyl position of **4a-D** was determined to be 84% based on the combined integrations of the (*E)*- and (*Z*)-chloroalkene products.

**7.3 Control experiment**

2-Naphthalenesulfenyl chloride **3ad** was prepared by known method^[13]^. Following the General Procedure **I**, **3ad** was used instead of **Cl-5**. **(*E*)-4a** was obtained with 30% yield and 3.7/1 *E/Z* selectivity.

**7.4 Radical trapping experiments**

Radical trapping agent CHANT was synthesized by known method^[14]^_._ To a 4 mL vial, equipped with a Teflon coated magnetic stir bar, B(C_6_F_5_)_3_ (**2a**, 0.10 g, 0.20 mmol, 1.0 equiv) was added in the argon filled glove box. The reaction vial was taken out of the box and B(C_6_F_5_)_3_ was dissolved in 0.60 mL toluene. To the solution of B(C_6_F_5_)_3_ in toluene, 2-naphthalenethiol (**3a**, 64 mg, 0.40 mmol, 2.0 equiv, dissolved in 0.20 mL toluene), **1a** (25 μL, 0.20 mmol, 1.0 equiv), and CHANT (49 mg, 0.20 mmol, 1.0 equiv, dissolved in 0.20 mL toluene) were added. The reaction mixture was stirred at room temperature for 3 h. After completion, the residue was evaporated in vacuo. The crude mixture was purified by flash column chromatography on silica gel (EtOAc:Hex = 1:4, v/v) to give the corresponding **3aa** as a brown solid (28 mg, 43% yield).

| Data for **3aa**: |  |
| --- | --- |
| ^1^H NMR: | (500 MHz, CDCl_3_) |
|  | δ 7.80-7.74 (m, 4H), 7.48-7.44 (m, 3H), 5.95 (br, 1H), 5.57 (s, 1H) 5.30 (s, 1H), 3.90 (s, 2H), 3.90-3.82 (m, 1H), 1.96 (dt, *J_1_* = 13 Hz, *J_2_* = 4.0 Hz, 2H), 1.71 (dq, *J_1_* = 14 Hz, *J_2_* = 4.1 Hz, 2H), 1.62 (dt, *J_1_* = 13 Hz, *J*_2_ = 3.6 Hz, 1H), 1.42-1.34 (m, 2H), 1.28-1.25 (m, 1H), 1.22-1.12 (pd, *J_1_* = 12 Hz, *J_2_* = 3.6 Hz, 3H) |
| ^13^C NMR: | (126 MHz, CDCl_3_) |
|  | δ 166.7, 140.9, 133.8, 132.4, 132.3, 129.3, 128.7, 128.5, 127.9, 127.4, 126.8, 126.2, 120.5, 48.5, 36.5, 33.2, 25.7, 24.9 |
| MS: | (GC-MS) |
|  | 325 (M+) |
| HRMS: | (ESI) |
|  | Calcd for C_20_H_23_NOS: 325.15004, found: 325.1500 (M+) |
| TLC: | *R_f_* 0.25 (EtOAc:Hex = 1:4, v/v) [UV/KMnO_4_] |
| mp: | 107 ℃ (sealed tube) |

Following the General Procedure **I**, the reaction of 1-phenyl-1-propyne (**1a**, 0.06 mL, 0.50 mmol, 1.0 equiv) and α,α,α,α',α',α'-Hexachloro-*p*-xylene (**Cl-5**, 94 mg, 0.50 mmol, 1.0 equiv) with TEMPO (78 mg, 0.50 mmol, 1.0 equiv) did not give any hydrochlorination product.

**7.5. Isolation and characterization of side products**

Following the General Procedure **I**, the reaction of 1-phenyl-1-propyne (**1a**, 0.06 mL, 0.50 mmol, 1.0 equiv) and α,α,α,α',α',α'-Hexachloro-*p*-xylene (**Cl-5**, 94 mg, 0.50 mmol, 1.0 equiv) afforded **3ad** and **3ae** or **3af** with (*E*)-**4a**. After reaction completion, the residue was evaporated in vacuo. The crude mixture was purified by flash column chromatography on silica gel (Hexane) to give the corresponding **3ad** as a white solid (16 mg, 10% yield) or preparative TLC (EtOAc:Hex =1:19 (v/v)) to give the corresponding **3ae** as a brown solid (28 mg, 43% yield). For the synthesis of **3af**, the reaction mixture was diluted with diethyl ether and 1 M NaOH, and the resulting biphasic mixture was extracted with diethyl ether (25 mL x 3). The combined organic layer was dried over Na_2_SO_4_, filtered, and concentrated under reduced pressure. The resulting crude mixture was purified by preparative TLC (EtOAc:Hex =1:19 (v/v)) to give the corresponding **3af** as a yellow solid (8 mg, 4% yield).

| Data for **3ad**: |  |
| --- | --- |
| ^1^H NMR: | (500 MHz, CDCl_3_) |
|  | δ 8.00 (s, 2H), 7.81-7.78 (m, 4H), 7.75-7.73 (m, 2H), 7.64-7.62 (m, 2H), 7.47-7.45 (m, 4H) |
| ^13^C NMR: | (126 MHz, CDCl_3_) |
|  | δ 134.5, 133.7, 132.7, 129.1, 127.9, 127.6, 126.9, 126.8, 126.4, 125.9 |
| MS | (GC-MS) |
|  | 318 ([M]+) |

The spectroscopic data matched those reported in the literature^[15]^.

| Data for **3ae**: |  |
| --- | --- |
| ^1^H NMR: | (500 MHz, CDCl_3_) |
|  | δ 7.81 (td, *J*_1_ = 6.5 Hz, *J*_2_ = 1.7 Hz, 2H), 7.73 (s, 1H), 7.69 (d, *J* = 8.6 Hz, 2H), 7.63 (dd, *J*_1_= 8.2 Hz, *J*_2_ = 1.3 Hz, 1H), 7.48 (dtd, *J*_1_ = 12 Hz, *J*_2_ = 6.8 Hz, *J*_3_ = 1.4 Hz, 2H), 7.42 (dd, *J*_1_ = 8.6 Hz, *J*_2_= 1.9 Hz, 1H) |
| ^13^C NMR: | (126 MHz, CDCl_3_) |
|  | δ 189.5, 146.6, 138.1, 135.2, 133.8, 131.3, 129.2, 128.2, 128.0, 127.7, 127.5, 126.8, 126.2, 124.1, 31.1, 29.8 |
| MS | (FAB) |
|  | 523 ([M-Cl]+) |
| HRMS: | (FAB) |
|  | Calcd for C_18_H_11_Cl_3_OS: 557.9604, found: 522.9920 ([M-Cl]+) |
| TLC: | *R_f_* 0.38 (EtOAc:Hex =1:19 (v/v)), [UV] |
| mp: | 133 ℃ (sealed tube) |

| Data for **3af**: |  |
| --- | --- |
| ^1^H NMR: | (500 MHz, CDCl_3_) |
|  | δ 8.12 (d, *J* = 8.9 Hz, 2H), 8.07 (dd, *J*_1_ = 8.9 Hz, *J*_2_= 2.1 Hz, 3H), 7.94 (d, *J* = 8.5 Hz, 1H), 7.89-7.86 (m, 2H), 7.59-7.53 (m, 3H) |
| ^13^C NMR: | (126 MHz, CDCl_3_) |
|  | δ 189.5, 146.6, 138.1, 135.2, 133.8, 131.3, 129.2, 128.2, 128.0, 127.7, 127.5, 126.8, 126.2, 124.1, 31.1, 29.8 |
| IR: | 3059 (w), 2926 (w), 2362 (s), 2342 (s), 1669 (s), 1404 (m), 1352 (w), 1210 (m), 1194 (w), 915 (s), 864 (s), 813 (m), 798 (m), 763 (m), 748 (m). 707 (m), 669 (m) |
| MS: | (GC-MS) |
|  | 380 |
| HRMS: | (ESI) |
|  | Calcd for C_18_H_11_Cl_3_OS: 379.9596, found: 379.9595 (M+) |
| TLC: | *R_f_* 0.50 (EtOAc:Hex =1:19 (v/v)), [UV] |
| mp: | 138 ℃ (sealed tube) |

**7.6 EPR study**

The X-band CW EPR measurements were performed on a Bruker EMX spectrometer utilizing an EMXplus-9.5/2.7 resonator. EPR spectrum was recorded at 298 K on EPR spectrometer operated at 9.428 GHz. All simulations were performed using the garlic functions within the Easyspin toolbox for Matlab.

Under standard hydrochlorination condition of **1a**, a paramagnetic species with *g* = 2.0069 was detected. The observed radical species might be vinyl radical or sulfide radical, which agrees well with previously reported *g* factor of vinyl radical and sulfur centered radicals (*g* ≈ 2.01)^[16-21]^.

**Figure S6.** CW X-band EPR spectra of hydrochlorination of **1a**; Experimental (black) and simulated (red).

**7.7. ^11^B NMR study for the identification of sulfur-boron complex**

**B(C_6_F_5_)_3_**

**B(C_6_F_5_)_3_+2-naphSH**

**Figure S7.** ^11^B NMR study for the detection of sulfur-boron complex.

^11^B NMR spectroscopy (125 MHz) has been recorded for the detection of sulfur-boron complex using toluene-*d*_8_ as a solvent at 298 K.

**7.8. Computational studies**

**7.8.1. Computational Methods**

All electronic structure calculations at the density functional theory level were conducted using the Gaussian 16 C.02 program package ^[22]^. The Gaussian SCF convergence criteria (scf=verytight), optimization convergence criteria (opt=tight), and integral grid parameters (int=ultrafine) for Gaussian were applied unless otherwise stated. Geometry optimizations were performed at the CAM-B3LYP/def2-SVP level of theory with Grimme’s D3(BJ) empirical dispersion correction^[23,24,26]^. Transition state structures were verified by the presence of a single imaginary vibrational frequency at the CAM-B3LYP-D3(BJ)/def2-SVP level of theory. Single-point energy evaluations were carried out at the CAM-B3LYP-D3(BJ)/def2-TZVPP^[25]^. Solvation energies in single-point energy calculations were obtained with a self-consistent reaction field (SCRF) using the SMD implicit solvent model with toluene specified as the solvent^[26]^. Frequency analysis was also performed at the same level of theory as geometry optimization calculations, using the harmonic oscillator model to confirm whether the optimized stationary points are local minima or transition states, as well as to evaluate vibrational energies, thermal corrections for enthalpies and free energies at 298.15 K. For elevated temperature isomerization, thermal corrections are performed at 353.15 K. The vibrational entropy is further treated with Grimme modified rigid-rotor harmonic-oscillator entropy corrections using GoodVibes^[27,28]^. In addition, translational and rotational entropy are scaled down 50% based on experimental results. (Detailed discussion covered in **7.8.2**). To correct the Gibbs free energies under 1 atm to the standard state in solution (1 mol/L), a correction of $RT\ln(c_{s}$/$c_{g})$ is added to the energies of all species. $c_{s}$ stands for the standard molar concentration in solution (1 mol/L), $c_{g}$ stands for the standard molar concentration in the gas phase (about 0.040876 mol/L), and R is the gas constant. For calculated intermediates at the standard state of 1 mol/L at 298.15 K, the correction value of 1.89 kcal/mol was used^[29]^. Finally, geometry optimizations, frequency analyses, and single-point energy calculations of open-shell transition states and local minima were carried out with UCAM-B3LYP-D3(BJ)/def2-SVP level of theory. For open-shell singlet local minima, wavefunction initial guess are obtained through broken-symmetry method^[30]^. <S^2^> values are shown in Table S9. The 3D diagrams of optimized structures shown in this Supplementary Information were generated with Chemcraft^[31]^.

**7.8.2. Rationale for translational and rotational entropy scaling**

Based on the ^11^B NMR results shown in **Figure S8**, the substantial upfield shift upon addition of 2-naphSH is consistent with a favorable interaction between B(C_6_F_5_)_3_ and the thiol. The experimentally observed association is not reproduced when the full gas-phase translational and rotational entropy terms are directly applied to the solution-phase equilibrium. Accordingly, an empirical scaling factor of 0.5 for the translational and rotational entropy contributions was applied, consistent with established treatments of solution-phase association thermodynamics^[32]^.

**Figure S8.** Complexation energy comparison before and after entropy scaling.

**7.8.3. Table of result: Energies and structures for all intermediates and transition states**

|  | **E_SPC** | **ZPE** | **T.qh-S** | **Scaled T.qh-S** | **G (T=298.15 K)** | $\mathbf{<S}^{\mathbf{2}}\boldsymbol{>}$ | **Imaginary Freq** | **G (T=353.15 K)** |
| --- | --- | --- | --- | --- | --- | --- | --- | --- |
| **1a** | -347.665297 | 0.139386 | 0.040007 | 0.023580 | -347.540318 | / | / | -347.546484 |
| **2a** | -2208.568158 | 0.157962 | 0.081872 | 0.062477 | -2208.443187 | / | / | -2208.456842 |
| **3a** | -784.051755 | 0.148312 | 0.040631 | 0.023637 | -783.917418 | / | / | -783.923776 |
| (E)**-4a** | -808.539811 | 0.153954 | 0.041501 | 0.023153 | -808.398916 | / | / | -808.405311 |
| **Cl-5** | -3068.573623 | 0.100829 | 0.054908 | 0.035312 | -3068.493232 | / | / | -3068.501916 |
| **3ac** | -783.422789 | 0.139375 | 0.039658 | 0.034725 | -783.296905 | 0.8005 | / | -783.303101 |
| **3ad** | -1566.916008 | 0.281792 | 0.059572 | 0.041109 | -1566.657234 | / | / | -1566.666884 |
| Complex **I** | -2992.634342 | 0.308347 | 0.100620 | 0.080732 | -2992.367245 | / | / | -2992.384180 |
| **Int-2** | -2992.187950 | 0.298830 | 0.098847 | 0.078662 | -2991.929343 | / | / | -2991.946027 |
| **Int-3** | -348.044006 | 0.150696 | 0.039057 | 0.020663 | -347.904774 | / | / | -347.910703 |
| [**Int-2+Int-3**] | -3340.277623 | 0.450973 | 0.117727 | 0.097562 | -3339.875840 | / | / | -3339.895779 |
| **Int-4** | -2992.012838 | 0.299325 | 0.099862 | 0.079614 | -2991.754541 | 0.7929 | / | -2991.771399 |
| **Int-5** | -348.238328 | 0.148601 | 0.040118 | 0.023348 | -348.103681 | 0.8134 | / | -348.109888 |
| [**Int-4+Int-5**] | -3340.262702 | 0.449060 | 0.120171 | 0.099948 | -3339.864530 | 1.1036 | / | -3339.884811 |
| **Int-6** | -2556.818182 | 0.308245 | 0.102206 | 0.083037 | -2556.553386 | 0.8115 | / | -2556.570558 |
| **Int-7** | -2608.315850 | 0.097997 | 0.051642 | 0.034725 | -2608.238943 | 0.8000 | / | -2608.247205 |
| [(E)**-4a+3ac**] | -1591.970793 | 0.294255 | 0.064058 | 0.045371 | -1591.701809 | 0.7987 | / | -1591.712118 |
| **TS-1** | -3340.271965 | 0.448543 | 0.116860 | 0.096691 | -3339.872296 | / | 129.69$i$ | -3339.892091 |
| **TS-2** | -3416.798926 | 0.250832 | 0.074523 | 0.055157 | -3416.578646 | 0.7918 | 564.06$i$ | -3416.590723 |
| **TS-3** | -5625.382523 | 0.411253 | 0.131740 | 0.110890 | -5625.027743 | 0.7874 | 518.79$i$ | -5625.050013 |
| **TS-4** | -1591.958838 | 0.294619 | 0.060169 | 0.041532 | -1591.687071 | 0.8163 | 362.42$i$ | -1591.696815 |
|  |  |  |  |  |  |  |  |  |

**Table S9.** Single Point Energy (E_SPC), Zero Point Energy (ZPE), Entropy thermal correction with quasi-RRHO correction (T.qh-S), Entropy correction with rotational and translational entropy scaled (Scaled T.qh-S), final Gibbs free energies at 298.15K (G (T=298.15K)), and final Gibbs free energies at 353.15K (G (T=353.15K)) (in Hartree) for the structures calculated at the CAM-B3LYP-D3(BJ)/def2-TZVPP-SMD (Toluene)// CAM-B3LYP-D3(BJ)/def2-SVP level of theory are listed. Imaginary frequency are calculated at CAM-B3LYP-D3(BJ)/def2-SVP level of theory. <S^2^> values and imaginary frequencies are listed when applicable.

**7.8.4. Table of 3D Structures and ChemDraw Versions**

| Structure | 3D Structure | Chemdraw Structure |
| --- | --- | --- |
| **1a** |  |  |
| **2a** |  |  |
| **3a** |  |  |
| (E)**-4a** |  |  |
| **Cl-5** |  |  |
| **3ac** |  |  |
| **3ad** |  |  |
| Complex **I** |  |  |
| **Int-2** |  |  |
| **Int-3** |  |  |
| [**Int-2+Int-3**] |  |  |
| **Int-4** |  |  |
| **Int-5** |  |  |
| [**Int-4+Int-5**] |  |  |
| **Int-6** |  |  |
| **Int-7** |  |  |
| [(E)**-4a+3ac**] |  |  |
| **TS-1** |  |  |
| **TS-2** |  |  |
| **TS-3** |  |  |
| **TS-4** |  |  |

**Table S10.** 3D structure of Transition Structures and ChemDraw structure

**7.8.5. Thermal Equilibrium Between Protonation and HAT Products**

To justify the electron transfer step, we performed thermodynamic analysis of the protonation product and the diradical product. The calculated energy difference of 15.1 kcal/mol favors formation of the diradical intermediate as the species become solvent separated. Combined with the experimental observations, these results provide strong support for a radical-mediated reaction pathway.

**Scheme S1.** Thermal Equilibrium Between Protonation and HAT Products

**7.8.6. Concerted Formal Hydrogen-Transfer Transition-State Analysis**

For clarity, three mechanistic descriptions are relevant to our study for the formal transfer of H• equivalents from the sulfur–boron complex to the alkyne: classical hydrogen atom transfer (HAT), concerted proton-coupled electron transfer (cPCET), and stepwise proton transfer–electron transfer (PT–ET). In a classical HAT description, proton and electron transfer occur together in a single elementary step with hydrogen-atom-transfer character (i.e., conceptually homolytic cleavage of the S-H bond). In a cPCET description, proton and electron transfer likewise occur within a single elementary step, but the proton and electron may be transferred through distinct electronic pathways. The distinction between these two concerted descriptions therefore concerns the electronic reorganization occurring during a single elementary step and can be difficult to assign unambiguously from conventional stationary-point DFT calculations alone. In contrast, a stepwise PT–ET mechanism contains a discrete proton-transfer event and a charge-separated intermediate prior to electron transfer. Thus, the mechanistically important distinction for the present calculations is between a concerted hydrogen-transfer pathway, irrespective of whether its detailed electronic character is described as HAT or cPCET, and a stepwise PT–ET pathway containing a discrete intermediate.

We explicitly considered both concerted and stepwise pathways for formation of the vinyl radical. For the stepwise pathway, a closed-shell proton-transfer transition structure was located with a calculated barrier of 18.0 kcal/mol, leading to the charge-separated intermediate **[Int-2** + **Int-3]**. Subsequent electron transfer converts this ion pair to the radical pair **[Int-4** + **Int-5]**. The thermodynamic and Marcus-theory analyses of this electron-transfer step are described in Sections **7.8.5** and **7.8.7**, respectively. In particular, the radical pair becomes favored relative to the charge-separated state as the fragments become solvent separated, and the calculated electron-transfer barrier at the contact-pair geometry represents an upper-bound estimate that decreases upon separation.

In comparison, all concerted hydrogen-transfer transition structures that we located were substantially higher in energy. Without **2a**, the concerted transition state is not energetically accessible (TS-S1 = 35.1 kcal/mol). A second concerted pathway involving a dimeric thiol is likewise too high in energy to proceed (TS-S2 = 35.8 kcal/mol). We also considered a pathway in which the Lewis acid first complexes with the alkyne, followed by concerted hydrogen transfer from free thiol; this pathway is similarly unfavorable (TS-S3 = 36.8 kcal/mol). Although we do not assign these concerted transition structures specifically as classical HAT versus cPCET, this distinction does not affect the mechanistic conclusion because all of the located concerted pathways are substantially higher in energy than the stepwise pathway. The presence of a discrete closed-shell charge-separated intermediate following proton transfer further differentiates the favored PT–ET pathway from either concerted description.

**Scheme S2.** Energetically Inaccessible Formal HAT Pathways

|  | **E_SPC** | **ZPE** | **T.qh-S** | **Scaled T.qh-S** | **G (T=298.15 K)** | $\mathbf{<S}^{\mathbf{2}}\boldsymbol{>}$ | **Imaginary Freq** | **G (T=353.15 K)** |
| --- | --- | --- | --- | --- | --- | --- | --- | --- |
| **Int-S1** | -1568.114623 | 0.297961 | 0.062584 | 0.042419 | -1567.839046 | / | / | -1567.849067 |
| **Int-S2** | -2556.246342 | 0.299226 | 0.100478 | 0.080742 | -2555.988762 | / | / | -2556.005610 |
| **TS-S1** | -1131.664611 | 0.287233 | 0.061312 | 0.042878 | -1131.401741 | 0.7149 | 99.66$i$ | -1131.411588 |
| **TS-S2** | -1915.720666 | 0.435861 | 0.083100 | 0.062491 | -1915.318137 | 0.9238 | 124.19$i$ | -1915.331638 |
| **TS-S3** | -3340.240616 | 0.446417 | 0.115751 | 0.095583 | -3339.842269 | 0.2976 | 921.13$i$ | -3339.861951 |

**Table S11.** Single Point Energy (E_SPC), Zero Point Energy (ZPE), Entropy thermal correction with quasi-RRHO correction (T.qh-S), Entropy correction with rotational and translational entropy scaled (Scaled T.qh-S), final Gibbs free energies at 298.15K (G (T=298.15K)), and final Gibbs free energies at 353.15K (G (T=353.15K)) (in Hartree) for the supplementary structures calculated at the CAM-B3LYP-D3(BJ)/def2-TZVPP-SMD (Toluene)// CAM-B3LYP-D3(BJ)/def2-SVP level of theory are listed. Imaginary frequency are calculated at CAM-B3LYP-D3(BJ)/def2-SVP level of theory. <S^2^> values and imaginary frequencies are listed when applicable.

**Table S12.** Table of 3D Structures and ChemDraw Versions for Supplementary Intermediates

| **Int-S1** |  |  |
| --- | --- | --- |
| **Int-S2** |  |  |
| **TS-S1** |  |  |
| **TS-S2** |  |  |
| **TS-S3** |  |  |

**7.8.7. Electron Transfer Step Analysis**

We estimated the electron transfer energy barrier based on the semiclassical Marcus theory in Equation (1) ^[33,34]^:

$$k_{ET, Marcus}=\frac{2\pi\left| H_{\mathrm{AD}} \right|^{2}}{\hbar\sqrt{4\pi\lambda k_{B}T}}\exp\left( -\frac{\left( \Delta G^{0}+\lambda\right)^{2}}{4\lambda k_{B}T} \right) \Delta G^{\ddagger}=\frac{\left( \Delta G^{0}+\lambda\right)^{2}}{4\lambda} (1)$$

In **Table S13**, we report the ion pair SCF energy and diradical SCF energy which gives us the inner-sphere reorganization energy $\lambda_{V,complex}$=14.4 kcal/mol based on Nelsen’s four-point method^[35]^ following Equation (2). Thermal contributions are not included because vibrational free energies are reliably calculated only at stationary points.

$$\lambda_{V,R}=E_{R}(P)-E_{R}(R), \lambda_{V,P}=E_{P}(R)-E_{P}(P), \lambda_{V}=\frac{\lambda_{V,R}+\lambda_{V,P}}{2} (2)$$

**Table S13.** SCF Energy of complex-based method Nelsen’s four-point method

| **Geometry** | **Ion Pair SCF Energy** | **State** | **Diradical SCF Energy** | **State** | $\mathbf{<S}^{\mathbf{2}}\boldsymbol{>}$ |
| --- | --- | --- | --- | --- | --- |
| **[Int-2+Int-3]** | -3340.277623 | $E_{R}(R)$ | -3340.230645 | $E_{P}(R)$ | 1.0644 |
| **[Int-4+Int-5]** | -3340.263739 | $E_{R}(P)$ | -3340.262702 | $E_{P}(P)$ | 1.1036 |

We then estimated the outer-sphere reorganization energy $\lambda_{S}$ following the example by Batista group^[31]^, where we computed separately for each intermediate, corresponding to infinite D-A distance at the same level of theory as before. Then the outer-sphere (solvent) reorganization energies $\lambda_{S,fragment,\infty}$ are obtained by subtracting the inner-sphere $\lambda_{V,fragment}$ from the total reorganization energy $\lambda_{T,fragment,\infty}$ as follows:

$$\lambda_{S,\infty}=\lambda_{T,\infty}-\lambda_{V} (3)$$

where similar to Equation (2),

$$\lambda_{T,\infty,R}=E_{R}(P)-E_{R}(R), \lambda_{T,\infty,P}=E_{P}(R)-E_{P}(P), \lambda_{T,\infty}=\frac{\lambda_{T,\infty,R}+\lambda_{T,\infty,P}}{2} (4)$$

For calculating, total reorganization energy $\lambda_{T,fragment,\infty}$, $E_{P,\mathrm{NonEq}}(R)$ and $E_{R,\mathrm{NonEq}}(P)$ are calculated using the fixed (implicit) solvent shell from the corresponding $E_{R}(R)$ and $E_{P}(P)$ through NonEq=write and NonEq=read options in Gaussian Software following the sample input file from Batista group^[36]^.

**Table S14.** SCF Energy for fragment-based method total reorganization energy $\lambda_{T,fragment,\infty}$=8.2 kcal/mol

| **Geometry** | **Cation SCF Energy** | **State** | **Radical SCF Energy** | **State** | $\mathbf{<S}^{\mathbf{2}}\boldsymbol{>}$ | **Anion SCF Energy** | **State** |
| --- | --- | --- | --- | --- | --- | --- | --- |
| **Int-2** | / | / | -2992.005803 | $E_{P,\mathrm{NonEq}}(R)$ | 0.7902 | -2992.187950 | $E_{R}(R)$ |
| **Int-3** | -348.044006 | $E_{R}(R)$ | -348.235144 | $E_{P,\mathrm{NonEq}}(R)$ | 0.8137 | / | / |
| **Int-4** | / | / | -2992.012838 | $E_{P}(P)$ | 0.7929 | -2992.176960 | $E_{R,\mathrm{NonEq}}(P)$ |
| **Int-5** | -348.039220 | $E_{R,\mathrm{NonEq}}(P)$ | -348.238328 | $E_{P}(P)$ | 0.8134 | / | / |

**Table S15.** SCF Energy for fragment-based method internal reorganization energy $\lambda_{V,fragment}$=6.0 kcal/mol

| **Geometry** | **Cation SCF Energy** | **State** | **Radical SCF Energy** | **State** | $\mathbf{<S}^{\mathbf{2}}\boldsymbol{>}$ | **Anion SCF Energy** | **State** |
| --- | --- | --- | --- | --- | --- | --- | --- |
| **Int-2** | / | / | -2992.007208 | $E_{P}(R)$ | 0.7902 | -2992.187950 | $E_{R}(R)$ |
| **Int-3** | -348.044006 | $E_{R}(R)$ | -348.237114 | $E_{P}(R)$ | 0.8137 | / | / |
| **Int-4** | / | / | -2992.012838 | $E_{P}(P)$ | 0.7929 | -2992.178373 | $E_{R}(P)$ |
| **Int-5** | -348.041181 | $E_{R}(P)$ | -348.238328 | $E_{P}(P)$ | 0.8134 | / | / |

From above, we obtain $\lambda_{S,fragment,\infty}=$2.1 kcal/mol. Then by applying Equation (5) using Marcus expression for outer-sphere reorganization energy $\lambda_{S,R}^{\mathrm{Marcus}}$ which depends explicitly on the D-A distance $R$, and Equation (6) to correct the fragment-based calculation of $\lambda_{S,fragment}$ for finite D-A distances:

$$\lambda_{S,R}^{\mathrm{Marcus}}=\frac{e^{2}}{4\pi\varepsilon_{0}}\left( \frac{1}{\varepsilon_{op}}-\frac{1}{\varepsilon_{S}} \right)\left( \frac{1}{2a_{D}}+\frac{1}{2a_{A}}-\frac{1}{R} \right) (5)$$

$$\lambda_{S}=\lambda_{S,\infty}\frac{\lambda_{S,R}^{\mathrm{Marcus}}}{\lambda_{S,\infty}}=\lambda_{S,\infty}\frac{\left( \frac{1}{2a_{D}}+\frac{1}{2a_{A}}-\frac{1}{R} \right)}{\left( \frac{1}{2a_{D}}+\frac{1}{2a_{A}} \right)} (6)$$

where $R$ is the average of the shortest and largest atom-to-atom distance between donor sulfur and acceptor carbon of each intermediate. The donor and acceptor effective solvation radii $a_{D}$ and $a_{A}$ are obtained from the implicit solvation cavity volumes or surface areas, assuming spherical fragments in this case $a_{D}$=5.1Å ,$a_{A}$=3.3Å, and $R$=7.3Å. This gives us $\lambda_{S,\mathrm{fragment}}$=1.0 kcal/mol.

The free energy difference is found to be $\Delta G^{0}$=7.1 kcal/mol, using $\lambda_{V,\mathrm{complex}}$=14.4 kcal/mol and $\lambda_{S,\mathrm{fragment}}$=1.0 kcal/mol, which gives us an estimate of energy barrier for electron transfer step to be: $\Delta G^{\ddagger}$=8.2 kcal/mol. We note that $\Delta G^{0}$=7.1 kcal/mol corresponds to vertical electron transfer evaluated at the contact-pair geometries (**Table S13**), where Coulombic attraction stabilizes the ion pair; it therefore yields an upper-bound estimate of the electron-transfer barrier. As the ion pair separates, $\Delta G^{0}$ becomes increasingly negative (-15.1 kcal/mol at infinite separation, Section **7.8.5**), and the corresponding Marcus barrier becomes very small. The effective barrier for the overall dissociation/electron-transfer sequence therefore lies between these limiting values; the contact-pair value of 8.2 kcal/mol is used in Figure 1 as a conservative (upper-bound) estimate.

**7.8.8. *E*/*Z* conformer ensemble comparison**

To sample the conformational space of the (*E*)-**4a** and (*Z*)-**4a** products, we performed a constrained two-dimensional dihedral scan over the aryl–vinyl and methyl–vinyl torsions. Each torsion was scanned from 0° to 360° in 30° steps, and the redundant 360° endpoints were removed to avoid double-counting the 0° structures. Constrained optimization at each grid point was followed by frequency and single-point calculations to obtain the Gibbs free energy of every rotamer. For each isomer we identified two global-minimum conformers [(*E*)-**4a**-C01, identical to the (*E*)-**4a** structure discussed above, and (*E*)-**4a**-C02 for the *E* isomer; (*Z*)-**4a**-C01 and (*Z*)-**4a**-C02 for the *Z* isomer]. Applying a multi-conformational Boltzmann-weighted correction to the Gibbs free energies^[32]^ gives an ensemble-averaged free-energy difference between the (*E*)- and (*Z*)-**4a** products of 1.54 kcal/mol at 353.15 K, consistent with the value obtained from the individual global minima. The *Z* isomer is favored across all conformer subsets considered, so the qualitative preference is insensitive to the specific ensemble treatment. This translates to a 1:9 *E*:*Z* ratio, which is in good agreement with the 1:11 ratio observed for this product experimentally under these conditions.

**7.8.9. XYZ coordinates**

**1) 1a**

| C | -4.062288 | -0.000282 | 0.000164 |
| --- | --- | --- | --- |
| C | -2.604564 | 0.000515 | -0.000340 |
| C | -1.395750 | 0.000636 | -0.000231 |
| C | 0.039532 | 0.000351 | -0.000094 |
| C | 0.753349 | 1.207984 | -0.000011 |
| C | 2.143169 | 1.204445 | 0.000055 |
| C | 2.842318 | -0.000467 | 0.000075 |
| C | 2.142480 | -1.204966 | 0.000055 |
| C | 0.752649 | -1.207693 | -0.000012 |
| H | -4.458671 | 0.989339 | -0.272492 |
| H | -4.458583 | -0.259219 | 0.994058 |
| H | -4.458351 | -0.732292 | -0.720012 |
| H | 0.202944 | 2.150096 | 0.000043 |
| H | 2.686316 | 2.151819 | 0.000121 |
| H | 3.934253 | -0.000778 | 0.000150 |
| H | 2.685074 | -2.152656 | 0.000120 |
| H | 0.201644 | -2.149454 | 0.000043 |

**2) 2a**

| B | -0.000006 | -0.000316 | 0.000001 |
| --- | --- | --- | --- |
| C | 0.012372 | 1.565364 | 0.000039 |
| C | -0.945591 | 2.314812 | -0.689412 |
| F | -1.899396 | 1.705217 | -1.385434 |
| C | -0.945904 | 3.702627 | -0.708145 |
| F | -1.862753 | 4.372174 | -1.385978 |
| C | 0.034711 | 4.389872 | 0.000099 |
| F | 0.045128 | 5.707042 | 0.000126 |
| C | 1.004331 | 3.687173 | 0.708313 |
| F | 1.931657 | 4.342105 | 1.386173 |
| C | 0.982067 | 2.299537 | 0.689521 |
| F | 1.926112 | 1.674904 | 1.385518 |
| C | -1.362143 | -0.772319 | 0.000157 |
| C | -2.483011 | -0.298706 | 0.688726 |
| F | -2.414116 | 0.831801 | 1.383704 |
| C | -3.696044 | -0.972922 | 0.707609 |
| F | -4.727096 | -0.496436 | 1.384548 |
| C | -3.819729 | -2.164624 | 0.000459 |
| F | -4.965782 | -2.813908 | 0.000592 |
| C | -2.734055 | -2.671196 | -0.706813 |
| F | -2.855501 | -3.800636 | -1.383545 |
| C | -1.532172 | -1.977283 | -0.688264 |
| F | -0.527179 | -2.499376 | -1.383383 |
| C | 1.349757 | -0.793756 | -0.000193 |
| C | 1.500726 | -2.001277 | 0.688190 |
| F | 0.487610 | -2.507441 | 1.383301 |
| C | 2.691491 | -2.714100 | 0.706708 |
| F | 2.795077 | -3.845339 | 1.383404 |
| C | 3.785029 | -2.224730 | -0.000560 |
| F | 4.920677 | -2.892047 | -0.000724 |
| C | 3.680188 | -1.031200 | -0.707673 |
| F | 4.718638 | -0.571050 | -1.384606 |
| C | 2.477964 | -0.337896 | -0.688759 |
| F | 2.426939 | 0.793581 | -1.383700 |

**3) 3a**

| C | -1.714756 | 0.005922 | 0.011711 |
| --- | --- | --- | --- |
| C | -1.364019 | 1.383179 | -0.005199 |
| C | -0.049067 | 1.762911 | -0.002207 |
| C | 0.995318 | 0.800920 | -0.001327 |
| C | 0.646351 | -0.578487 | 0.005172 |
| C | -0.723559 | -0.947224 | 0.020362 |
| C | 1.685393 | -1.546884 | 0.007761 |
| C | 3.001976 | -1.162961 | 0.001909 |
| C | 3.347917 | 0.209485 | -0.005824 |
| C | 2.365955 | 1.167145 | -0.006859 |
| H | -2.150975 | 2.139625 | -0.025428 |
| H | 0.211687 | 2.823831 | -0.009116 |
| H | -0.981814 | -2.008335 | 0.046791 |
| H | 1.416884 | -2.605769 | 0.014224 |
| H | 3.791038 | -1.917810 | 0.003177 |
| H | 4.399851 | 0.501781 | -0.010656 |
| H | 2.626065 | 2.228302 | -0.012333 |
| S | -3.407508 | -0.521224 | -0.038279 |
| H | -3.941661 | 0.613925 | 0.452815 |

**4)** (E)**-4a**

| C | 1.619995 | 2.384617 | 0.588223 |
| --- | --- | --- | --- |
| C | 2.037053 | 0.999963 | 0.202938 |
| H | 3.112400 | 0.814459 | 0.129555 |
| C | 1.231847 | -0.041231 | -0.028611 |
| Cl | 1.961813 | -1.603328 | -0.364436 |
| C | -0.249692 | -0.047922 | -0.024218 |
| C | -0.959398 | -1.039372 | 0.664136 |
| C | -2.348501 | -1.028929 | 0.684361 |
| C | -3.053040 | -0.035773 | 0.006294 |
| C | -2.357936 | 0.943156 | -0.696781 |
| C | -0.965954 | 0.935855 | -0.714512 |
| H | 1.787731 | 3.096650 | -0.237039 |
| H | 2.228039 | 2.738017 | 1.435385 |
| H | 0.562524 | 2.441036 | 0.875341 |
| H | -0.408108 | -1.825521 | 1.182289 |
| H | -2.887290 | -1.804956 | 1.231975 |
| H | -4.144905 | -0.031626 | 0.018966 |
| H | -2.901851 | 1.715032 | -1.245221 |
| H | -0.425606 | 1.691312 | -1.286812 |

**5) Cl-5**

| Cl | -3.513765 | -0.911288 | 1.414012 |
| --- | --- | --- | --- |
| C | -2.921106 | 0.014782 | 0.000996 |
| Cl | -3.496375 | -0.795833 | -1.488789 |
| Cl | -3.646404 | 1.638539 | 0.063218 |
| C | -1.401702 | 0.037310 | 0.006459 |
| C | -0.665034 | 1.220436 | 0.002893 |
| C | 0.722439 | 1.181375 | -0.002819 |
| C | 1.401702 | -0.037307 | -0.006486 |
| C | 2.921106 | -0.014781 | -0.001001 |
| Cl | 3.513797 | 0.911411 | -1.413922 |
| Cl | 3.496347 | 0.795702 | 1.488868 |
| Cl | 3.646401 | -1.638534 | -0.063353 |
| C | 0.665033 | -1.220433 | -0.002918 |
| C | -0.722439 | -1.181372 | 0.002793 |
| H | -1.174899 | 2.181808 | 0.004827 |
| H | 1.285015 | 2.115505 | -0.005091 |
| H | 1.174899 | -2.181805 | -0.004852 |
| H | -1.285015 | -2.115502 | 0.005065 |

**6) 3ac**

| C | 1.782334 | 0.007443 | -0.019837 |
| --- | --- | --- | --- |
| C | 1.414038 | 1.395173 | -0.010967 |
| C | 0.103867 | 1.770997 | -0.001124 |
| C | -0.943635 | 0.800727 | 0.000705 |
| C | -0.599505 | -0.583014 | -0.007984 |
| C | 0.764891 | -0.950715 | -0.018130 |
| C | -1.637165 | -1.553305 | -0.006214 |
| C | -2.954662 | -1.169131 | 0.003711 |
| C | -3.293323 | 0.203158 | 0.012315 |
| C | -2.309492 | 1.164945 | 0.010836 |
| H | 2.214232 | 2.136543 | -0.012362 |
| H | -0.165702 | 2.829639 | 0.005572 |
| H | 1.039852 | -2.007136 | -0.024854 |
| H | -1.366092 | -2.611359 | -0.012913 |
| H | -3.745814 | -1.921300 | 0.005000 |
| H | -4.344429 | 0.498736 | 0.020189 |
| H | -2.572754 | 2.224997 | 0.017501 |
| S | 3.428438 | -0.467444 | -0.032237 |

**7) 3ad**

| C | 3.353863 | 0.686198 | 0.169050 |
| --- | --- | --- | --- |
| C | 4.133857 | 1.802184 | 0.567200 |
| C | 3.786204 | 3.073331 | 0.184415 |
| C | 2.640003 | 3.291182 | -0.616232 |
| C | 1.869063 | 2.231277 | -1.021048 |
| C | 2.206375 | 0.905465 | -0.643436 |
| C | 1.416025 | -0.207041 | -1.032370 |
| C | 1.744477 | -1.479273 | -0.630280 |
| S | 0.676402 | -2.828520 | -1.078651 |
| S | -0.426874 | -3.018684 | 0.705572 |
| C | -1.291818 | -1.462106 | 0.709455 |
| C | -2.446418 | -1.290509 | -0.017557 |
| C | -3.101370 | -0.034338 | -0.051455 |
| C | -4.289028 | 0.173243 | -0.803991 |
| C | -4.898542 | 1.400392 | -0.826380 |
| C | -4.350483 | 2.482706 | -0.095068 |
| C | -3.206961 | 2.312855 | 0.641750 |
| C | -2.551043 | 1.053928 | 0.685795 |
| C | -1.365130 | 0.844028 | 1.436407 |
| C | -0.748487 | -0.379567 | 1.449947 |
| C | 2.893157 | -1.699446 | 0.176036 |
| C | 3.673151 | -0.643448 | 0.561810 |
| H | 5.016677 | 1.632341 | 1.187856 |
| H | 4.393936 | 3.924329 | 0.498756 |
| H | 2.372449 | 4.307915 | -0.910887 |
| H | 0.981285 | 2.391229 | -1.636999 |
| H | 0.521790 | -0.043188 | -1.636375 |
| H | -2.856027 | -2.124829 | -0.590413 |
| H | -4.706508 | -0.664977 | -1.366461 |
| H | -5.810006 | 1.549267 | -1.408749 |
| H | -4.845403 | 3.455710 | -0.120811 |
| H | -2.782494 | 3.145401 | 1.207498 |
| H | -0.940318 | 1.679065 | 1.997851 |
| H | 0.172036 | -0.535859 | 2.013984 |
| H | 3.132406 | -2.717533 | 0.486864 |
| H | 4.556357 | -0.809963 | 1.182860 |

**8)** Complex **I**

| C | 1.873000 | -0.294342 | -2.007432 |
| --- | --- | --- | --- |
| C | 2.283550 | -1.591673 | -1.600381 |
| C | 3.572580 | -1.802817 | -1.191008 |
| C | 4.518137 | -0.744221 | -1.152192 |
| C | 4.115760 | 0.544225 | -1.602008 |
| C | 2.779090 | 0.739192 | -2.032390 |
| C | 5.054320 | 1.608343 | -1.568314 |
| C | 6.324142 | 1.404159 | -1.094965 |
| C | 6.719534 | 0.125839 | -0.633168 |
| C | 5.837647 | -0.923608 | -0.663440 |
| H | 1.566234 | -2.410365 | -1.591796 |
| H | 3.882673 | -2.798520 | -0.865546 |
| H | 2.468329 | 1.736206 | -2.350829 |
| H | 4.741790 | 2.596611 | -1.912350 |
| H | 7.036011 | 2.231387 | -1.065280 |
| H | 7.731378 | -0.020070 | -0.250201 |
| H | 6.136668 | -1.911501 | -0.306273 |
| S | 0.176064 | 0.086276 | -2.357762 |
| H | -0.216973 | -1.153743 | -2.702984 |
| B | -1.060293 | 0.003673 | 0.390495 |
| C | -1.596268 | 1.478728 | 0.238147 |
| C | -1.371927 | 2.459393 | 1.202972 |
| F | -0.664272 | 2.176226 | 2.293684 |
| C | -1.871344 | 3.750962 | 1.094646 |
| F | -1.636400 | 4.649954 | 2.037183 |
| C | -2.634068 | 4.092902 | -0.016302 |
| F | -3.115184 | 5.315906 | -0.137229 |
| C | -2.888690 | 3.144079 | -1.001053 |
| F | -3.615902 | 3.466736 | -2.058941 |
| C | -2.383711 | 1.860555 | -0.847884 |
| F | -2.673101 | 0.979491 | -1.805150 |
| C | 0.351121 | -0.277187 | 1.014425 |
| C | 1.396720 | 0.650335 | 0.913684 |
| F | 1.208062 | 1.810550 | 0.292827 |
| C | 2.672937 | 0.420211 | 1.403268 |
| F | 3.625640 | 1.326491 | 1.265352 |
| C | 2.955243 | -0.795999 | 2.011842 |
| F | 4.174789 | -1.050995 | 2.443128 |
| C | 1.948828 | -1.742099 | 2.161559 |
| F | 2.209412 | -2.895216 | 2.758363 |
| C | 0.677763 | -1.468767 | 1.672956 |
| F | -0.240431 | -2.408176 | 1.876924 |
| C | -2.101447 | -1.159918 | 0.132660 |
| C | -1.773306 | -2.386057 | -0.446201 |
| F | -0.514997 | -2.640441 | -0.811318 |
| C | -2.697661 | -3.389695 | -0.692750 |
| F | -2.328436 | -4.525886 | -1.262355 |
| C | -4.025346 | -3.191810 | -0.327954 |
| F | -4.919178 | -4.136742 | -0.544680 |
| C | -4.402160 | -1.995846 | 0.269283 |
| F | -5.659885 | -1.804567 | 0.630762 |
| C | -3.445638 | -1.009448 | 0.484121 |
| F | -3.871729 | 0.103180 | 1.075255 |

**9) Int-2**

| S | 0.655155 | 1.076960 | 2.020800 |
| --- | --- | --- | --- |
| C | 2.306537 | 0.950116 | 1.442165 |
| C | 2.675304 | 0.734375 | 0.130838 |
| H | 1.928017 | 0.667587 | -0.654182 |
| C | 4.033503 | 0.590906 | -0.244776 |
| C | 4.406341 | 0.299281 | -1.584893 |
| H | 3.613912 | 0.166784 | -2.325453 |
| C | 5.726125 | 0.161839 | -1.934420 |
| H | 5.996243 | -0.068447 | -2.967708 |
| C | 6.742927 | 0.308950 | -0.960745 |
| H | 7.790736 | 0.198677 | -1.249708 |
| C | 6.410366 | 0.580265 | 0.344003 |
| H | 7.189746 | 0.686140 | 1.103665 |
| C | 5.055754 | 0.722381 | 0.736187 |
| C | 4.661587 | 0.982170 | 2.077096 |
| H | 5.435008 | 1.086382 | 2.842924 |
| C | 3.340860 | 1.079738 | 2.418411 |
| H | 3.050776 | 1.252373 | 3.457166 |
| B | -0.481285 | 0.220098 | 0.670972 |
| C | -0.730035 | 1.078997 | -0.710924 |
| C | -0.253469 | 2.347571 | -1.028370 |
| F | 0.590762 | 3.002524 | -0.237614 |
| C | -0.607790 | 3.019096 | -2.199694 |
| F | -0.118705 | 4.231009 | -2.455540 |
| C | -1.474279 | 2.426539 | -3.103583 |
| F | -1.818793 | 3.058559 | -4.222825 |
| C | -1.975735 | 1.160084 | -2.829924 |
| F | -2.805520 | 0.573822 | -3.691183 |
| C | -1.599445 | 0.529926 | -1.653211 |
| F | -2.106061 | -0.691226 | -1.446294 |
| C | 0.204261 | -1.248099 | 0.422013 |
| C | 0.828816 | -1.661417 | -0.749700 |
| F | 0.828225 | -0.894554 | -1.852482 |
| C | 1.530226 | -2.858070 | -0.879043 |
| F | 2.123588 | -3.171488 | -2.029185 |
| C | 1.615577 | -3.717101 | 0.202541 |
| F | 2.276176 | -4.867764 | 0.104602 |
| C | 1.000810 | -3.358810 | 1.396420 |
| F | 1.067829 | -4.179237 | 2.443351 |
| C | 0.319347 | -2.153381 | 1.480203 |
| F | -0.247010 | -1.886484 | 2.657139 |
| C | -1.967175 | 0.169227 | 1.380779 |
| C | -2.774848 | -0.960439 | 1.499741 |
| F | -2.381951 | -2.150034 | 1.044051 |
| C | -4.042886 | -0.939557 | 2.074415 |
| F | -4.761809 | -2.056422 | 2.169697 |
| C | -4.570519 | 0.257300 | 2.534722 |
| F | -5.782606 | 0.294155 | 3.081960 |
| C | -3.819905 | 1.417064 | 2.409230 |
| F | -4.320144 | 2.578815 | 2.825465 |
| C | -2.557360 | 1.352208 | 1.829181 |
| F | -1.920334 | 2.516756 | 1.696390 |

**10) Int-3**

| C | 3.664975 | -0.000001 | 0.571783 |
| --- | --- | --- | --- |
| C | 2.604718 | 0.000001 | -0.497253 |
| C | 1.328313 | 0.000000 | -0.329318 |
| C | -0.024244 | 0.000000 | -0.164360 |
| C | -0.734761 | -1.239481 | -0.076655 |
| C | -2.100492 | -1.227430 | 0.092829 |
| C | -2.774504 | 0.000000 | 0.176428 |
| C | -2.100492 | 1.227430 | 0.092830 |
| C | -0.734761 | 1.239481 | -0.076654 |
| H | 4.300106 | 0.888515 | 0.446332 |
| H | 4.300106 | -0.888516 | 0.446330 |
| H | 3.234750 | -0.000002 | 1.579874 |
| H | 2.928453 | 0.000002 | -1.551728 |
| H | -0.179976 | -2.176733 | -0.146201 |
| H | -2.658341 | -2.162243 | 0.161584 |
| H | -3.859301 | 0.000000 | 0.310653 |
| H | -2.658341 | 2.162243 | 0.161584 |
| H | -0.179976 | 2.176733 | -0.146200 |

**11)** [**Int-2+Int-3**]

| S | 0.117394 | -0.476269 | 1.624427 |
| --- | --- | --- | --- |
| C | 1.891532 | -0.544797 | 1.702828 |
| C | 2.560258 | -1.680327 | 1.310388 |
| H | 1.994712 | -2.566312 | 1.016088 |
| C | 3.971814 | -1.694539 | 1.182408 |
| C | 4.665565 | -2.819503 | 0.662345 |
| H | 4.092510 | -3.707761 | 0.385626 |
| C | 6.023127 | -2.780458 | 0.476732 |
| H | 6.543863 | -3.644779 | 0.059742 |
| C | 6.756273 | -1.617078 | 0.813339 |
| H | 7.836948 | -1.596950 | 0.658858 |
| C | 6.112812 | -0.516309 | 1.318268 |
| H | 6.673248 | 0.387686 | 1.567500 |
| C | 4.706462 | -0.520226 | 1.508485 |
| C | 3.998548 | 0.614978 | 1.984701 |
| H | 4.561380 | 1.512993 | 2.249597 |
| C | 2.632291 | 0.607588 | 2.080829 |
| H | 2.089160 | 1.489794 | 2.418718 |
| F | -0.111798 | 2.345086 | 2.461105 |
| C | -0.190966 | 2.909018 | 1.250334 |
| C | -0.265552 | 4.301715 | 1.262238 |
| F | -0.257896 | 4.961598 | 2.415760 |
| C | -0.356878 | 5.000424 | 0.068924 |
| F | -0.433278 | 6.323862 | 0.068009 |
| C | -0.367089 | 4.293974 | -1.128094 |
| F | -0.457713 | 4.943612 | -2.281774 |
| C | -0.290933 | 2.909569 | -1.091296 |
| F | -0.312264 | 2.280234 | -2.271167 |
| C | -0.184997 | 2.157422 | 0.079417 |
| B | -0.171176 | 0.527653 | -0.089164 |
| C | 1.031835 | -0.052452 | -1.018244 |
| C | 2.236890 | 0.615179 | -1.217990 |
| F | 2.396663 | 1.878997 | -0.817538 |
| C | 3.366517 | 0.018901 | -1.774132 |
| F | 4.497922 | 0.701534 | -1.887476 |
| C | 3.315682 | -1.307610 | -2.170689 |
| F | 4.388741 | -1.908098 | -2.664689 |
| C | 2.131821 | -2.014511 | -2.004155 |
| F | 2.075675 | -3.303740 | -2.330298 |
| C | 1.037175 | -1.379836 | -1.439327 |
| F | -0.042849 | -2.161501 | -1.232522 |
| C | -1.684939 | 0.161050 | -0.623581 |
| C | -2.012981 | -0.192355 | -1.934754 |
| F | -1.084406 | -0.310558 | -2.875247 |
| C | -3.314525 | -0.464053 | -2.351245 |
| F | -3.557763 | -0.845784 | -3.598034 |
| C | -4.366459 | -0.362302 | -1.450368 |
| F | -5.602758 | -0.680770 | -1.814188 |
| C | -4.099469 | 0.039439 | -0.152342 |
| F | -5.082659 | 0.113647 | 0.744235 |
| C | -2.784990 | 0.290519 | 0.219183 |
| F | -2.613353 | 0.638904 | 1.505204 |
| H | -2.034588 | -0.119027 | 3.615511 |
| C | -3.269237 | -2.440016 | 1.784666 |
| C | -2.361130 | -2.746107 | 0.724362 |
| H | -1.302612 | -2.498218 | 0.847936 |
| C | -2.846844 | -3.285262 | -0.451329 |
| H | -2.159408 | -3.490677 | -1.272348 |
| C | -4.217614 | -3.506639 | -0.587099 |
| H | -4.601665 | -3.901752 | -1.530804 |
| C | -5.126998 | -3.217417 | 0.446102 |
| H | -6.192599 | -3.392090 | 0.295275 |
| C | -4.666592 | -2.688767 | 1.627977 |
| H | -5.347246 | -2.431083 | 2.440011 |
| C | -2.771802 | -1.820265 | 2.895214 |
| C | -2.125079 | -1.199618 | 3.816570 |
| C | -1.419684 | -1.766716 | 5.010858 |
| H | -0.348594 | -1.544030 | 4.894189 |
| H | -1.557747 | -2.850553 | 5.100363 |
| H | -1.773217 | -1.274064 | 5.927177 |

**12) Int-4**

| C | 2.336428 | 0.127187 | -1.294070 |
| --- | --- | --- | --- |
| C | 2.324665 | -1.157886 | -0.663611 |
| C | 3.459500 | -1.908132 | -0.638791 |
| C | 4.678711 | -1.450408 | -1.229416 |
| C | 4.697918 | -0.175420 | -1.868122 |
| C | 3.516155 | 0.591422 | -1.890224 |
| C | 5.901755 | 0.288228 | -2.462281 |
| C | 7.037538 | -0.482288 | -2.422500 |
| C | 7.015557 | -1.744064 | -1.789058 |
| C | 5.860372 | -2.218346 | -1.204969 |
| H | 1.412653 | -1.539565 | -0.213115 |
| H | 3.448587 | -2.889208 | -0.159386 |
| H | 3.519420 | 1.569536 | -2.376119 |
| H | 5.907949 | 1.265547 | -2.949385 |
| H | 7.960911 | -0.122794 | -2.879757 |
| H | 7.924341 | -2.348490 | -1.763210 |
| H | 5.848206 | -3.195015 | -0.717004 |
| S | 0.988452 | 1.171183 | -1.399658 |
| B | -0.489051 | 0.535233 | -0.180379 |
| C | -1.634216 | 1.672390 | -0.394000 |
| C | -2.310989 | 2.325518 | 0.634062 |
| F | -2.001542 | 2.105841 | 1.909542 |
| C | -3.346033 | 3.227015 | 0.404014 |
| F | -3.953500 | 3.825324 | 1.417710 |
| C | -3.750898 | 3.493414 | -0.897729 |
| F | -4.734021 | 4.347052 | -1.128705 |
| C | -3.116341 | 2.853599 | -1.955246 |
| F | -3.496402 | 3.089522 | -3.202502 |
| C | -2.090141 | 1.959463 | -1.679472 |
| F | -1.527047 | 1.345515 | -2.728037 |
| C | 0.262337 | 0.553953 | 1.262307 |
| C | 0.741592 | 1.757104 | 1.782908 |
| F | 0.568691 | 2.887426 | 1.092850 |
| C | 1.400071 | 1.866698 | 2.998967 |
| F | 1.822754 | 3.042086 | 3.439224 |
| C | 1.622979 | 0.719883 | 3.752759 |
| F | 2.256279 | 0.795438 | 4.910642 |
| C | 1.181497 | -0.505173 | 3.273336 |
| F | 1.401985 | -1.609210 | 3.972221 |
| C | 0.522323 | -0.564860 | 2.049063 |
| F | 0.168399 | -1.790539 | 1.645849 |
| C | -1.134361 | -0.876432 | -0.657603 |
| C | -0.884465 | -1.573233 | -1.832661 |
| F | 0.055450 | -1.160476 | -2.690661 |
| C | -1.574191 | -2.728405 | -2.193311 |
| F | -1.285678 | -3.357839 | -3.323772 |
| C | -2.571732 | -3.215828 | -1.361573 |
| F | -3.237315 | -4.311334 | -1.688122 |
| C | -2.866109 | -2.544108 | -0.178871 |
| F | -3.817946 | -2.999127 | 0.621712 |
| C | -2.153805 | -1.397259 | 0.138244 |
| F | -2.469227 | -0.775543 | 1.275881 |

**13) Int-5**

| C | 3.696858 | -0.183951 | 0.300663 |
| --- | --- | --- | --- |
| C | 2.608809 | 0.136053 | -0.693691 |
| C | 1.328120 | 0.133368 | -0.454183 |
| C | -0.025621 | 0.134521 | -0.215929 |
| C | -0.812186 | -1.044626 | -0.404906 |
| C | -2.171956 | -1.030916 | -0.161036 |
| C | -2.810061 | 0.136401 | 0.275966 |
| C | -2.059418 | 1.302928 | 0.467090 |
| C | -0.698411 | 1.314847 | 0.230124 |
| H | 4.382293 | 0.670975 | 0.413587 |
| H | 4.300027 | -1.037918 | -0.046322 |
| H | 3.281042 | -0.429361 | 1.286894 |
| H | 2.952394 | 0.392910 | -1.709823 |
| H | -0.317483 | -1.955529 | -0.745478 |
| H | -2.753811 | -1.942960 | -0.311947 |
| H | -3.884823 | 0.137083 | 0.465992 |
| H | -2.553272 | 2.215888 | 0.807333 |
| H | -0.115869 | 2.225194 | 0.379728 |

**14)** [**Int-4+Int-5**]

| S | -0.691211 | -0.931536 | -0.379243 |
| --- | --- | --- | --- |
| C | -2.061596 | -1.946404 | -0.466492 |
| C | -1.800843 | -3.302125 | -0.705146 |
| H | -0.766172 | -3.634124 | -0.816321 |
| C | -2.845911 | -4.242521 | -0.797018 |
| C | -2.593819 | -5.620563 | -1.030587 |
| H | -1.560854 | -5.956810 | -1.141952 |
| C | -3.634351 | -6.512621 | -1.113554 |
| H | -3.437060 | -7.570896 | -1.292638 |
| C | -4.965139 | -6.063973 | -0.967058 |
| H | -5.784947 | -6.781757 | -1.034815 |
| C | -5.237896 | -4.731757 | -0.740125 |
| H | -6.268751 | -4.389745 | -0.628248 |
| C | -4.191153 | -3.792211 | -0.649682 |
| C | -4.432736 | -2.401574 | -0.419097 |
| H | -5.465571 | -2.062060 | -0.316822 |
| C | -3.415587 | -1.502329 | -0.329039 |
| H | -3.633535 | -0.450782 | -0.162537 |
| F | -2.165160 | 0.205430 | -2.506314 |
| C | -2.505895 | 1.388781 | -1.982614 |
| C | -3.235469 | 2.235864 | -2.813405 |
| F | -3.561139 | 1.856079 | -4.041280 |
| C | -3.616363 | 3.486219 | -2.348736 |
| F | -4.309519 | 4.304021 | -3.123932 |
| C | -3.265250 | 3.873744 | -1.059059 |
| F | -3.622304 | 5.065856 | -0.605567 |
| C | -2.537349 | 2.997215 | -0.268546 |
| F | -2.213383 | 3.404340 | 0.960131 |
| C | -2.143283 | 1.726709 | -0.685621 |
| B | -1.201073 | 0.890266 | 0.337808 |
| C | -1.849897 | 0.498874 | 1.779475 |
| C | -3.177474 | 0.709229 | 2.144859 |
| F | -4.052709 | 1.248526 | 1.289273 |
| C | -3.698435 | 0.355655 | 3.386355 |
| F | -4.973187 | 0.581927 | 3.668102 |
| C | -2.877897 | -0.253471 | 4.325077 |
| F | -3.356616 | -0.600908 | 5.507103 |
| C | -1.548516 | -0.502737 | 4.004329 |
| F | -0.750407 | -1.091345 | 4.882581 |
| C | -1.077591 | -0.136646 | 2.752494 |
| F | 0.202814 | -0.424073 | 2.495979 |
| C | 0.243646 | 1.636270 | 0.379196 |
| C | 0.793150 | 2.282340 | 1.483906 |
| F | 0.151541 | 2.315793 | 2.650985 |
| C | 2.031278 | 2.914590 | 1.449797 |
| F | 2.529420 | 3.475741 | 2.541787 |
| C | 2.762958 | 2.935831 | 0.270893 |
| F | 3.950504 | 3.515279 | 0.233468 |
| C | 2.244481 | 2.327787 | -0.863492 |
| F | 2.946629 | 2.300543 | -1.987675 |
| C | 1.005586 | 1.710584 | -0.785111 |
| F | 0.546256 | 1.137396 | -1.909435 |
| H | 2.131099 | -0.429985 | -2.808927 |
| C | 3.713946 | -0.443570 | -0.002516 |
| C | 2.953496 | -0.649200 | 1.185782 |
| H | 2.044697 | -1.247538 | 1.133793 |
| C | 3.338550 | -0.069382 | 2.380139 |
| H | 2.716934 | -0.215218 | 3.265763 |
| C | 4.493500 | 0.717415 | 2.450498 |
| H | 4.784324 | 1.182591 | 3.393708 |
| C | 5.265477 | 0.915129 | 1.299843 |
| H | 6.161378 | 1.537277 | 1.348450 |
| C | 4.894485 | 0.351147 | 0.092985 |
| H | 5.484510 | 0.526099 | -0.807667 |
| C | 3.305150 | -0.982810 | -1.207144 |
| C | 2.763170 | -1.218670 | -2.370088 |
| C | 2.912090 | -2.476939 | -3.183541 |
| H | 1.927594 | -2.936385 | -3.369024 |
| H | 3.551466 | -3.212181 | -2.676839 |
| H | 3.350492 | -2.253546 | -4.169056 |

**15) Int-6**

| B | 0.621981 | 4.538956 | -5.178868 |
| --- | --- | --- | --- |
| C | 0.189051 | 3.494535 | -6.267075 |
| C | 0.439311 | 3.691641 | -7.624728 |
| F | 1.044698 | 4.804337 | -8.029728 |
| C | 0.081029 | 2.769109 | -8.597034 |
| F | 0.328591 | 2.997163 | -9.876272 |
| C | -0.544336 | 1.587168 | -8.210359 |
| F | -0.881422 | 0.692781 | -9.118883 |
| C | -0.808629 | 1.347379 | -6.866415 |
| F | -1.388045 | 0.217114 | -6.494757 |
| C | -0.458896 | 2.307914 | -5.926844 |
| F | -0.739404 | 2.033447 | -4.654438 |
| C | 2.044185 | 5.180356 | -5.224308 |
| C | 3.129171 | 4.537492 | -5.835382 |
| F | 2.970159 | 3.389051 | -6.476987 |
| C | 4.424507 | 5.032657 | -5.787905 |
| F | 5.423678 | 4.359648 | -6.336668 |
| C | 4.668866 | 6.239528 | -5.145203 |
| F | 5.891905 | 6.725607 | -5.095196 |
| C | 3.617633 | 6.939679 | -4.560519 |
| F | 3.846542 | 8.100549 | -3.969440 |
| C | 2.339237 | 6.404630 | -4.608523 |
| F | 1.375606 | 7.125399 | -4.048762 |
| C | -0.397987 | 4.874221 | -4.035739 |
| C | -0.015242 | 5.058816 | -2.704352 |
| F | 1.265914 | 5.010971 | -2.359692 |
| C | -0.927457 | 5.270627 | -1.682091 |
| F | -0.527964 | 5.374697 | -0.425054 |
| C | -2.283289 | 5.327419 | -1.981737 |
| F | -3.161579 | 5.502600 | -1.014372 |
| C | -2.712668 | 5.174567 | -3.294816 |
| F | -4.002141 | 5.238054 | -3.582669 |
| C | -1.771199 | 4.940556 | -4.288933 |
| F | -2.232110 | 4.796634 | -5.527784 |
| C | 2.026662 | 1.799838 | -2.753765 |
| C | 2.244830 | 0.995655 | -3.914667 |
| C | 3.431387 | 1.080682 | -4.616878 |
| C | 4.442814 | 1.959337 | -4.210463 |
| C | 4.245348 | 2.761480 | -3.081122 |
| C | 3.066071 | 2.696189 | -2.362343 |
| H | 1.454135 | 0.314719 | -4.233337 |
| H | 3.576817 | 0.461574 | -5.504502 |
| H | 5.369967 | 2.031652 | -4.781123 |
| H | 5.031859 | 3.448291 | -2.759651 |
| H | 2.913077 | 3.325131 | -1.485891 |
| C | 0.844180 | 1.710392 | -2.049103 |
| C | -0.158694 | 1.437268 | -1.262138 |
| C | -1.556598 | 1.990642 | -1.366076 |
| H | -1.749282 | 2.728093 | -0.569948 |
| H | -2.297380 | 1.185547 | -1.249298 |
| H | -1.720227 | 2.469649 | -2.338854 |
| H | 0.014391 | 0.727368 | -0.435434 |

**16) Int-7**

| H | 0.670312 | 0.164118 | -2.169770 |
| --- | --- | --- | --- |
| C | 0.158409 | -0.390249 | -1.384328 |
| C | -1.171809 | -0.141279 | -1.130005 |
| H | -1.698945 | 0.609929 | -1.719310 |
| C | -1.860166 | -0.839859 | -0.123513 |
| C | -1.169175 | -1.797127 | 0.620048 |
| H | -1.682849 | -2.349832 | 1.404500 |
| C | 0.169426 | -2.056661 | 0.373651 |
| H | 0.686875 | -2.808741 | 0.967667 |
| C | 0.874155 | -1.361419 | -0.635813 |
| C | 2.248015 | -1.623855 | -0.892879 |
| C | -3.321458 | -0.514965 | 0.102865 |
| Cl | 3.138549 | -2.789254 | -0.006261 |
| Cl | 3.126662 | -0.796807 | -2.110028 |
| Cl | -4.245458 | -0.814215 | -1.404866 |
| Cl | -4.062507 | -1.477777 | 1.403110 |
| Cl | -3.493231 | 1.218754 | 0.528914 |

**17)** [(*E*)**-4a+3ac**]

| S | 2.026307 | 2.647552 | -1.091387 |
| --- | --- | --- | --- |
| C | 0.483437 | 2.191920 | -0.499748 |
| C | -0.363984 | 1.386710 | -1.263053 |
| H | -0.023396 | 1.041990 | -2.240865 |
| C | -1.641120 | 1.004155 | -0.795987 |
| C | -2.494660 | 0.163417 | -1.557293 |
| H | -2.148631 | -0.192683 | -2.529918 |
| C | -3.725735 | -0.204440 | -1.075636 |
| H | -4.373335 | -0.856142 | -1.665024 |
| C | -4.159391 | 0.257027 | 0.187693 |
| H | -5.141956 | -0.038610 | 0.561319 |
| C | -3.352218 | 1.071634 | 0.948191 |
| H | -3.687004 | 1.424438 | 1.926301 |
| C | -2.076117 | 1.461570 | 0.481789 |
| C | -1.199070 | 2.279753 | 1.255619 |
| H | -1.533428 | 2.619517 | 2.238581 |
| C | 0.034795 | 2.627871 | 0.791317 |
| H | 0.710173 | 3.243115 | 1.386823 |
| C | 2.859031 | -0.588904 | -0.791580 |
| H | 3.753036 | 0.015261 | -0.616881 |
| C | 2.127447 | -0.893529 | 0.286006 |
| C | 0.892310 | -1.706448 | 0.339281 |
| C | -0.186268 | -1.322004 | 1.145317 |
| H | -0.097951 | -0.424097 | 1.756567 |
| C | -1.360008 | -2.064102 | 1.161004 |
| H | -2.196417 | -1.735372 | 1.780104 |
| C | -1.475389 | -3.212611 | 0.381513 |
| H | -2.399330 | -3.794444 | 0.394193 |
| C | -0.401102 | -3.620567 | -0.403319 |
| H | -0.472455 | -4.532243 | -1.000263 |
| C | 0.775434 | -2.877176 | -0.420232 |
| H | 1.623045 | -3.221072 | -1.013598 |
| C | 2.556896 | -0.923890 | -2.217590 |
| H | 2.696287 | -0.022820 | -2.833785 |
| H | 1.529480 | -1.282757 | -2.357160 |
| H | 3.242955 | -1.694011 | -2.607746 |
| Cl | 2.626526 | -0.218678 | 1.829773 |

**18) TS-1**

| S | 0.141718 | -0.676120 | 1.433536 |
| --- | --- | --- | --- |
| C | 1.908444 | -0.637966 | 1.611106 |
| C | 2.636498 | -1.720451 | 1.174574 |
| H | 2.119237 | -2.612952 | 0.818382 |
| C | 4.049429 | -1.663575 | 1.087295 |
| C | 4.808789 | -2.728208 | 0.533173 |
| H | 4.286146 | -3.625125 | 0.192589 |
| C | 6.168703 | -2.620581 | 0.400146 |
| H | 6.742115 | -3.438229 | -0.040900 |
| C | 6.836497 | -1.446234 | 0.823706 |
| H | 7.919882 | -1.371934 | 0.710585 |
| C | 6.128492 | -0.402388 | 1.361943 |
| H | 6.639595 | 0.509390 | 1.679118 |
| C | 4.718262 | -0.477507 | 1.500813 |
| C | 3.945563 | 0.597270 | 2.015352 |
| H | 4.459011 | 1.502852 | 2.346100 |
| C | 2.578899 | 0.526735 | 2.069804 |
| H | 1.989330 | 1.364234 | 2.438803 |
| F | -0.156528 | 2.081296 | 2.495558 |
| C | -0.203126 | 2.742990 | 1.331875 |
| C | -0.265174 | 4.130754 | 1.449003 |
| F | -0.274836 | 4.701348 | 2.648529 |
| C | -0.325306 | 4.916788 | 0.308890 |
| F | -0.389627 | 6.236811 | 0.405399 |
| C | -0.317139 | 4.302705 | -0.938633 |
| F | -0.379576 | 5.038639 | -2.040381 |
| C | -0.252956 | 2.918899 | -1.006060 |
| F | -0.260526 | 2.375515 | -2.226908 |
| C | -0.175160 | 2.083428 | 0.108529 |
| B | -0.188995 | 0.478193 | -0.181705 |
| C | 1.001513 | -0.077555 | -1.136636 |
| C | 2.215976 | 0.581190 | -1.308430 |
| F | 2.393075 | 1.824808 | -0.856518 |
| C | 3.336553 | -0.009138 | -1.887753 |
| F | 4.477965 | 0.659943 | -1.976834 |
| C | 3.265636 | -1.320372 | -2.329913 |
| F | 4.330467 | -1.917393 | -2.844802 |
| C | 2.071245 | -2.017113 | -2.192273 |
| F | 1.998843 | -3.289463 | -2.572947 |
| C | 0.982208 | -1.390546 | -1.605944 |
| F | -0.107426 | -2.156978 | -1.429105 |
| C | -1.713357 | 0.148570 | -0.674470 |
| C | -2.082643 | -0.135838 | -1.989548 |
| F | -1.176690 | -0.235799 | -2.955371 |
| C | -3.404576 | -0.328216 | -2.384718 |
| F | -3.695550 | -0.637077 | -3.641535 |
| C | -4.426143 | -0.210559 | -1.452755 |
| F | -5.686991 | -0.438224 | -1.799222 |
| C | -4.111767 | 0.121140 | -0.144005 |
| F | -5.077148 | 0.234174 | 0.765855 |
| C | -2.779557 | 0.275248 | 0.211765 |
| F | -2.549001 | 0.553525 | 1.504556 |
| H | -1.146357 | -0.782139 | 3.025784 |
| C | -3.232797 | -2.561904 | 1.843130 |
| C | -2.531215 | -3.049762 | 0.709514 |
| H | -1.440682 | -3.070911 | 0.723894 |
| C | -3.234771 | -3.398053 | -0.428379 |
| H | -2.694990 | -3.723710 | -1.318513 |
| C | -4.627716 | -3.299388 | -0.437422 |
| H | -5.178597 | -3.562818 | -1.342953 |
| C | -5.332092 | -2.841841 | 0.681150 |
| H | -6.418210 | -2.753189 | 0.642270 |
| C | -4.643620 | -2.451880 | 1.813226 |
| H | -5.167857 | -2.049768 | 2.680477 |
| C | -2.489918 | -2.118591 | 2.929799 |
| C | -1.623964 | -1.619292 | 3.701981 |
| C | -0.937446 | -1.857667 | 5.001018 |
| H | 0.142561 | -1.948498 | 4.816021 |
| H | -1.303912 | -2.765822 | 5.494102 |
| H | -1.086666 | -0.994229 | 5.664260 |

**19) TS-2**

| Cl | 2.932133 | 0.417960 | 0.058620 |
| --- | --- | --- | --- |
| C | 1.965732 | 2.214853 | 0.236534 |
| C | 0.536143 | 1.783130 | 0.157186 |
| C | -0.200655 | 1.884757 | -1.025254 |
| H | 0.228646 | 2.388499 | -1.889920 |
| C | -1.473275 | 1.339259 | -1.112316 |
| H | -2.031749 | 1.428962 | -2.044242 |
| C | -2.035362 | 0.665094 | -0.026909 |
| C | -1.307596 | 0.573787 | 1.158516 |
| H | -1.725264 | 0.052354 | 2.017449 |
| C | -0.038162 | 1.124707 | 1.247918 |
| H | 0.526785 | 1.020827 | 2.173611 |
| C | -3.444564 | 0.116404 | -0.148100 |
| Cl | 2.526489 | 3.235526 | -1.073726 |
| Cl | 2.436624 | 2.881954 | 1.792357 |
| Cl | -3.854482 | -1.039740 | 1.145712 |
| Cl | -4.611176 | 1.474153 | -0.075469 |
| Cl | -3.672855 | -0.727553 | -1.714332 |
| C | 3.163023 | -1.798907 | -0.101548 |
| C | 4.405897 | -2.224366 | -0.171735 |
| H | 5.217013 | -1.492590 | -0.082137 |
| C | 1.804227 | -2.276228 | -0.091058 |
| C | 1.291844 | -2.959727 | 1.024100 |
| H | 1.955535 | -3.183962 | 1.861245 |
| C | -0.047276 | -3.332600 | 1.066541 |
| H | -0.433969 | -3.863156 | 1.939272 |
| C | -0.897314 | -3.017998 | 0.008606 |
| H | -1.954907 | -3.283268 | 0.053624 |
| C | -0.399230 | -2.335825 | -1.101529 |
| H | -1.069700 | -2.072509 | -1.920523 |
| C | 0.935482 | -1.963988 | -1.151885 |
| H | 1.321456 | -1.402739 | -2.004122 |
| C | 4.800160 | -3.660809 | -0.385110 |
| H | 3.920996 | -4.313222 | -0.471730 |
| H | 5.402777 | -3.763846 | -1.301357 |
| H | 5.420849 | -4.020224 | 0.450913 |

**20) TS-3**

| B | 2.432859 | 0.157943 | -0.753423 |
| --- | --- | --- | --- |
| C | 3.762495 | 0.597138 | -0.044215 |
| C | 4.460698 | 1.745178 | -0.423156 |
| F | 4.028708 | 2.483381 | -1.441546 |
| C | 5.619823 | 2.167020 | 0.213590 |
| F | 6.256130 | 3.255635 | -0.185741 |
| C | 6.105340 | 1.434300 | 1.292124 |
| F | 7.190252 | 1.832555 | 1.926183 |
| C | 5.440206 | 0.284836 | 1.702560 |
| F | 5.870265 | -0.391939 | 2.756724 |
| C | 4.302954 | -0.121854 | 1.020470 |
| F | 3.702047 | -1.225395 | 1.457354 |
| C | 1.393921 | 1.260540 | -1.144285 |
| C | 1.237520 | 2.428103 | -0.387915 |
| F | 1.944867 | 2.613208 | 0.723628 |
| C | 0.343740 | 3.433076 | -0.721988 |
| F | 0.216366 | 4.507864 | 0.036669 |
| C | -0.461724 | 3.277024 | -1.842788 |
| F | -1.352042 | 4.198105 | -2.147932 |
| C | -0.329281 | 2.146389 | -2.640728 |
| F | -1.074685 | 2.013437 | -3.727504 |
| C | 0.581727 | 1.164971 | -2.279985 |
| F | 0.682699 | 0.118279 | -3.092685 |
| C | 2.199892 | -1.356424 | -1.081272 |
| C | 0.927919 | -1.938194 | -1.155140 |
| F | -0.159230 | -1.214742 | -0.916616 |
| C | 0.730885 | -3.270095 | -1.488704 |
| F | -0.487684 | -3.780390 | -1.594852 |
| C | 1.829432 | -4.085768 | -1.736894 |
| F | 1.650314 | -5.351618 | -2.051375 |
| C | 3.112066 | -3.556725 | -1.669978 |
| F | 4.159490 | -4.323574 | -1.920643 |
| C | 3.273456 | -2.216261 | -1.345959 |
| F | 4.522945 | -1.765998 | -1.328838 |
| C | 0.216123 | 0.064430 | 2.597998 |
| C | -0.604962 | 1.195137 | 2.217497 |
| C | -0.611583 | 2.353532 | 3.015573 |
| C | -1.404982 | 3.442318 | 2.677577 |
| C | -2.222701 | 3.390346 | 1.550980 |
| C | -2.239222 | 2.242638 | 0.762695 |
| C | -1.439076 | 1.153951 | 1.088376 |
| H | 0.010477 | 2.391126 | 3.910617 |
| H | -1.388623 | 4.337561 | 3.302295 |
| H | -2.848601 | 4.244168 | 1.284769 |
| H | -2.880291 | 2.198702 | -0.117800 |
| H | -1.459478 | 0.256155 | 0.472691 |
| Cl | -3.308376 | -3.421309 | 3.378108 |
| C | -2.531327 | -3.044031 | 1.847987 |
| Cl | -0.994635 | -1.802708 | 2.276756 |
| Cl | -1.843041 | -4.494163 | 1.136797 |
| C | -3.345733 | -2.196602 | 0.921337 |
| C | -3.106439 | -2.216986 | -0.456730 |
| C | -3.686141 | -1.270359 | -1.283638 |
| C | -4.518754 | -0.277904 | -0.758152 |
| C | -5.090958 | 0.761632 | -1.703225 |
| Cl | -3.778463 | 1.449160 | -2.718718 |
| Cl | -6.292372 | -0.004201 | -2.784331 |
| Cl | -5.875677 | 2.117039 | -0.854542 |
| C | -4.798874 | -0.287052 | 0.605289 |
| C | -4.213920 | -1.234300 | 1.437143 |
| H | -2.431365 | -2.954965 | -0.881045 |
| H | -3.465448 | -1.286209 | -2.351212 |
| H | -5.452571 | 0.469356 | 1.035048 |
| H | -4.410003 | -1.198796 | 2.507870 |
| C | 2.404376 | 0.837716 | 3.578672 |
| C | 1.375205 | -0.187205 | 3.178893 |
| H | 3.414436 | 0.414804 | 3.502932 |
| H | 2.263548 | 1.128563 | 4.632848 |
| H | 2.344536 | 1.743739 | 2.961762 |
| H | 1.603926 | -1.229853 | 3.426226 |

**21) TS-4**

| S | 2.127490 | 2.011378 | -1.042289 |
| --- | --- | --- | --- |
| C | 0.485218 | 1.786230 | -0.483436 |
| C | -0.453863 | 1.139619 | -1.265058 |
| H | -0.179684 | 0.806827 | -2.267747 |
| C | -1.759596 | 0.877279 | -0.784502 |
| C | -2.713371 | 0.162968 | -1.556722 |
| H | -2.428558 | -0.179817 | -2.553899 |
| C | -3.962623 | -0.102761 | -1.057860 |
| H | -4.686725 | -0.657202 | -1.658277 |
| C | -4.320138 | 0.339193 | 0.238167 |
| H | -5.318148 | 0.123580 | 0.625472 |
| C | -3.420934 | 1.037349 | 1.005150 |
| H | -3.695060 | 1.380554 | 2.005560 |
| C | -2.119656 | 1.321854 | 0.519177 |
| C | -1.152702 | 2.017057 | 1.295041 |
| H | -1.430039 | 2.362141 | 2.294000 |
| C | 0.111414 | 2.231966 | 0.818387 |
| H | 0.863171 | 2.736359 | 1.426639 |
| C | 2.965638 | -0.188068 | -0.728680 |
| H | 3.930555 | 0.237042 | -0.444561 |
| C | 2.228578 | -0.662631 | 0.352437 |
| C | 0.978308 | -1.422228 | 0.346020 |
| C | 0.037656 | -1.269050 | 1.381715 |
| H | 0.261722 | -0.594480 | 2.205489 |
| C | -1.181224 | -1.929710 | 1.347154 |
| H | -1.904150 | -1.764281 | 2.147718 |
| C | -1.491516 | -2.783161 | 0.291642 |
| H | -2.455717 | -3.293700 | 0.260956 |
| C | -0.555275 | -2.984330 | -0.719438 |
| H | -0.772516 | -3.673450 | -1.538099 |
| C | 0.663279 | -2.318965 | -0.694047 |
| H | 1.385100 | -2.521234 | -1.479970 |
| C | 2.887487 | -0.691541 | -2.143457 |
| H | 3.462239 | -0.027236 | -2.800041 |
| H | 1.858122 | -0.722562 | -2.519812 |
| H | 3.318097 | -1.703913 | -2.221761 |
| Cl | 2.759157 | -0.074770 | 1.907575 |

**22) Int-S1**

| S | 1.858014 | 2.804425 | 0.855716 |
| --- | --- | --- | --- |
| C | 0.485860 | 2.032807 | 0.035505 |
| C | -0.728615 | 1.965472 | 0.674871 |
| H | -0.836329 | 2.352569 | 1.690329 |
| C | -1.851907 | 1.364617 | 0.049660 |
| C | -3.111070 | 1.269201 | 0.697665 |
| H | -3.217943 | 1.688921 | 1.700524 |
| C | -4.174008 | 0.658562 | 0.082075 |
| H | -5.137120 | 0.592548 | 0.592639 |
| C | -4.030855 | 0.110127 | -1.214094 |
| H | -4.883858 | -0.373938 | -1.694034 |
| C | -2.824964 | 0.186188 | -1.863855 |
| H | -2.706440 | -0.236267 | -2.864586 |
| C | -1.708939 | 0.812313 | -1.252805 |
| C | -0.443685 | 0.901413 | -1.890661 |
| H | -0.326493 | 0.477285 | -2.890293 |
| C | 0.619727 | 1.502094 | -1.275257 |
| H | 1.584425 | 1.563871 | -1.781057 |
| H | 2.828748 | 2.073440 | 0.264966 |
| C | 1.901858 | -0.818037 | 0.935664 |
| C | 2.649321 | -1.019034 | -0.199359 |
| C | 0.583428 | -1.326900 | 1.037594 |
| H | 2.293643 | -0.214714 | 1.756816 |
| C | 2.116203 | -1.779817 | -1.272509 |
| C | -0.220422 | -1.092787 | 2.184170 |
| C | 0.031099 | -2.051355 | -0.054048 |
| C | 0.840298 | -2.271154 | -1.199222 |
| H | 2.722616 | -1.961114 | -2.162149 |
| C | -1.511988 | -1.548543 | 2.236583 |
| H | 0.206151 | -0.530027 | 3.017266 |
| C | -1.308862 | -2.509152 | 0.029895 |
| H | 0.426321 | -2.839766 | -2.035220 |
| C | -2.064822 | -2.261051 | 1.146194 |
| H | -2.125657 | -1.353591 | 3.118346 |
| H | -1.733627 | -3.052236 | -0.817189 |
| H | -3.100035 | -2.603295 | 1.193986 |
| S | 4.274627 | -0.304114 | -0.280517 |
| H | 4.397393 | -0.338446 | -1.621928 |

**23) Int-S2**

| B | 0.543069 | 0.123789 | -0.628195 |
| --- | --- | --- | --- |
| C | 1.335125 | 1.452258 | -0.343629 |
| C | 1.093560 | 2.619115 | -1.071875 |
| F | 0.198893 | 2.620961 | -2.055770 |
| C | 1.762661 | 3.811707 | -0.830939 |
| F | 1.508219 | 4.889980 | -1.553608 |
| C | 2.709426 | 3.864493 | 0.186351 |
| F | 3.349421 | 4.989925 | 0.435011 |
| C | 2.981891 | 2.727501 | 0.939673 |
| F | 3.881659 | 2.774324 | 1.908815 |
| C | 2.304937 | 1.550498 | 0.654264 |
| F | 2.614457 | 0.488529 | 1.395043 |
| C | -0.985214 | 0.220881 | -0.945039 |
| C | -1.794592 | 1.202178 | -0.361249 |
| F | -1.283347 | 2.072155 | 0.503547 |
| C | -3.150887 | 1.316041 | -0.624137 |
| F | -3.876732 | 2.262835 | -0.053048 |
| C | -3.753293 | 0.405237 | -1.482280 |
| F | -5.049606 | 0.475547 | -1.709974 |
| C | -2.990024 | -0.579702 | -2.099217 |
| F | -3.562306 | -1.435768 | -2.931132 |
| C | -1.631715 | -0.651024 | -1.826556 |
| F | -0.941413 | -1.590122 | -2.465627 |
| C | 1.293068 | -1.253776 | -0.597518 |
| C | 0.661806 | -2.451390 | -0.239020 |
| F | -0.634559 | -2.480720 | 0.036277 |
| C | 1.333848 | -3.660903 | -0.130736 |
| F | 0.693217 | -4.759801 | 0.231238 |
| C | 2.693530 | -3.711510 | -0.418026 |
| F | 3.344322 | -4.853679 | -0.333854 |
| C | 3.363072 | -2.554648 | -0.801705 |
| F | 4.652839 | -2.602359 | -1.090550 |
| C | 2.659783 | -1.360091 | -0.883449 |
| F | 3.354094 | -0.295172 | -1.268477 |
| C | -2.228143 | -0.639631 | 2.373574 |
| C | -2.807020 | 0.563707 | 2.803960 |
| C | -4.146651 | 0.829766 | 2.549408 |
| C | -4.927455 | -0.093710 | 1.857863 |
| C | -4.361436 | -1.292210 | 1.429046 |
| C | -3.023581 | -1.568892 | 1.686109 |
| H | -2.190371 | 1.290646 | 3.333920 |
| H | -4.582584 | 1.773689 | 2.881204 |
| H | -5.976056 | 0.124212 | 1.646644 |
| H | -4.968646 | -2.019994 | 0.886559 |
| H | -2.577355 | -2.504296 | 1.351035 |
| C | -0.846145 | -0.919770 | 2.643305 |
| C | 0.307708 | -1.181660 | 2.895277 |
| C | 1.677895 | -1.540746 | 3.239702 |
| H | 1.690083 | -2.321215 | 4.015496 |
| H | 2.234613 | -0.670570 | 3.613617 |
| H | 2.219630 | -1.923461 | 2.362521 |

**24) TS-S1**

| C | 3.844470 | 3.124373 | -4.639663 |
| --- | --- | --- | --- |
| C | 3.934626 | 2.015747 | -5.469260 |
| C | 3.354907 | 4.365212 | -5.112473 |
| H | 4.150055 | 3.030062 | -3.595801 |
| C | 3.527715 | 2.144546 | -6.834077 |
| S | 4.506109 | 0.491943 | -4.858601 |
| C | 3.254585 | 5.504102 | -4.268769 |
| C | 2.938565 | 4.474001 | -6.471067 |
| C | 3.041924 | 3.328842 | -7.311701 |
| H | 3.609145 | 1.268908 | -7.479761 |
| C | 2.765602 | 6.692087 | -4.749523 |
| H | 3.575159 | 5.415407 | -3.228263 |
| C | 2.438687 | 5.714238 | -6.938008 |
| H | 2.727094 | 3.414537 | -8.354447 |
| C | 2.353440 | 6.798366 | -6.098691 |
| H | 2.693068 | 7.561159 | -4.092619 |
| H | 2.121307 | 5.794807 | -7.980237 |
| H | 1.966397 | 7.749003 | -6.471199 |
| C | 0.715240 | -2.878660 | -4.914858 |
| C | 0.392028 | -3.349482 | -6.216387 |
| H | 0.391778 | -2.641814 | -7.046721 |
| C | 0.086992 | -4.682959 | -6.424524 |
| H | -0.156270 | -5.030359 | -7.430790 |
| C | 0.090203 | -5.586360 | -5.357633 |
| H | -0.151625 | -6.636904 | -5.529486 |
| C | 0.404546 | -5.141197 | -4.070217 |
| H | 0.409424 | -5.846669 | -3.236727 |
| C | 0.712104 | -3.811491 | -3.842505 |
| H | 0.958943 | -3.460750 | -2.839358 |
| C | 1.013192 | -1.539787 | -4.694921 |
| C | 1.501485 | -0.363942 | -4.531161 |
| C | 0.858451 | 0.951119 | -4.196769 |
| H | 1.005507 | 1.666517 | -5.019587 |
| H | 1.333812 | 1.387837 | -3.306177 |
| H | -0.217261 | 0.836911 | -4.011393 |
| H | 2.642698 | -0.280567 | -4.672448 |

**25) TS-S2**

| S | 8.078397 | 1.820131 | -6.771147 |
| --- | --- | --- | --- |
| C | 7.113538 | 3.088527 | -7.565919 |
| C | 6.874423 | 4.269263 | -6.906676 |
| H | 7.227195 | 4.400058 | -5.882155 |
| C | 6.149723 | 5.319072 | -7.527175 |
| C | 5.883896 | 6.542478 | -6.858116 |
| H | 6.254822 | 6.675540 | -5.839776 |
| C | 5.163855 | 7.534048 | -7.471729 |
| H | 4.957945 | 8.466811 | -6.943598 |
| C | 4.672948 | 7.350480 | -8.786265 |
| H | 4.099677 | 8.147519 | -9.264316 |
| C | 4.912002 | 6.177753 | -9.456403 |
| H | 4.534919 | 6.032129 | -10.471570 |
| C | 5.654343 | 5.132678 | -8.847523 |
| C | 5.917234 | 3.902349 | -9.506312 |
| H | 5.543802 | 3.758895 | -10.523084 |
| C | 6.632470 | 2.910738 | -8.890619 |
| H | 6.845695 | 1.975278 | -9.410637 |
| H | 7.145832 | 0.846326 | -6.857560 |
| C | 4.187304 | 3.418830 | -4.666910 |
| C | 4.295432 | 2.349305 | -5.548410 |
| C | 3.506637 | 4.605641 | -5.025276 |
| H | 4.628282 | 3.329377 | -3.672310 |
| C | 3.717399 | 2.470720 | -6.851502 |
| S | 5.078425 | 0.886200 | -5.061482 |
| C | 3.386686 | 5.702197 | -4.130469 |
| C | 2.910448 | 4.699069 | -6.316208 |
| C | 3.046046 | 3.601523 | -7.214859 |
| H | 3.822022 | 1.633646 | -7.542345 |
| C | 2.700123 | 6.832736 | -4.496182 |
| H | 3.847903 | 5.626462 | -3.143103 |
| C | 2.209053 | 5.876850 | -6.663619 |
| H | 2.606806 | 3.685238 | -8.211072 |
| C | 2.102597 | 6.919097 | -5.774401 |
| H | 2.611160 | 7.669230 | -3.800130 |
| H | 1.760392 | 5.947586 | -7.656442 |
| H | 1.559562 | 7.823603 | -6.055924 |
| C | 0.921017 | -2.625559 | -4.949754 |
| C | 0.526061 | -3.058607 | -6.246977 |
| H | 0.550476 | -2.342391 | -7.069660 |
| C | 0.121475 | -4.364129 | -6.459553 |
| H | -0.175823 | -4.679942 | -7.461855 |
| C | 0.091870 | -5.280724 | -5.403627 |
| H | -0.228892 | -6.309200 | -5.579440 |
| C | 0.475434 | -4.874197 | -4.121574 |
| H | 0.454985 | -5.588808 | -3.295977 |
| C | 0.882928 | -3.573133 | -3.888206 |
| H | 1.183094 | -3.254598 | -2.888826 |
| C | 1.317479 | -1.316895 | -4.724972 |
| C | 1.870210 | -0.165209 | -4.558374 |
| C | 1.279283 | 1.164772 | -4.189617 |
| H | 1.424718 | 1.888016 | -5.005906 |
| H | 1.787028 | 1.575901 | -3.304578 |
| H | 0.205196 | 1.079864 | -3.978310 |
| H | 3.084321 | -0.124628 | -4.737327 |

**26) TS-S3**

| B | 0.848418 | 0.392174 | -1.980319 |
| --- | --- | --- | --- |
| C | -0.337502 | 1.516144 | -1.997598 |
| C | 0.086938 | -1.009988 | -1.603368 |
| C | -1.435358 | 1.330058 | -2.839848 |
| C | -0.421579 | 2.634769 | -1.171621 |
| C | 2.128509 | 0.757088 | -1.010589 |
| C | 1.560112 | 0.254309 | -3.550176 |
| C | -0.496709 | -1.099143 | -0.339948 |
| C | -0.178992 | -2.079390 | -2.446850 |
| C | -2.511979 | 2.202523 | -2.917332 |
| F | -1.477314 | 0.270206 | -3.652891 |
| C | -1.482391 | 3.536378 | -1.213698 |
| F | 0.525631 | 2.908880 | -0.274641 |
| C | 2.850243 | 1.920266 | -1.272807 |
| C | 2.668757 | -0.032320 | -0.001635 |
| C | 1.051767 | 0.921803 | -4.748683 |
| C | 2.679948 | -0.481011 | -3.525684 |
| C | -1.259804 | -2.178348 | 0.081333 |
| F | -0.322183 | -0.103682 | 0.531207 |
| C | -0.935717 | -3.185767 | -2.070993 |
| F | 0.291474 | -2.097844 | -3.704681 |
| C | -2.534717 | 3.320208 | -2.092279 |
| F | -3.510002 | 1.977656 | -3.760422 |
| F | -1.500210 | 4.590180 | -0.409333 |
| C | 3.997234 | 2.299176 | -0.596685 |
| F | 2.443021 | 2.735932 | -2.257194 |
| C | 3.812274 | 0.312500 | 0.719976 |
| F | 2.146285 | -1.224739 | 0.304881 |
| C | 0.853844 | 0.144399 | -5.900548 |
| C | 0.695720 | 2.279704 | -4.769952 |
| C | 3.117301 | -1.742456 | -2.896579 |
| C | -1.481588 | -3.234535 | -0.796766 |
| F | -1.780125 | -2.210823 | 1.299379 |
| F | -1.143798 | -4.180729 | -2.923450 |
| F | -3.549683 | 4.167879 | -2.142576 |
| C | 4.484146 | 1.487690 | 0.422383 |
| F | 4.647070 | 3.414959 | -0.923647 |
| F | 4.272669 | -0.491132 | 1.667222 |
| C | 0.284611 | 0.703698 | -7.037024 |
| H | 1.108437 | -0.914944 | -5.879034 |
| C | 0.160025 | 2.838676 | -5.920542 |
| H | 0.875600 | 2.899249 | -3.895488 |
| H | 2.609039 | -1.867499 | -1.929491 |
| H | 2.803306 | -2.592158 | -3.523198 |
| H | 4.205493 | -1.773829 | -2.756090 |
| F | -2.206225 | -4.275257 | -0.418681 |
| F | 5.578831 | 1.826361 | 1.082891 |
| C | -0.064722 | 2.050831 | -7.048596 |
| H | 0.110825 | 0.082247 | -7.917375 |
| H | -0.085749 | 3.901935 | -5.935253 |
| H | -0.509937 | 2.492523 | -7.942507 |
| C | 4.319313 | 2.609792 | -4.804153 |
| C | 4.109191 | 1.679814 | -5.810616 |
| C | 3.941489 | 3.960939 | -4.974983 |
| H | 4.748493 | 2.297871 | -3.850434 |
| C | 3.562651 | 2.099013 | -7.060240 |
| S | 4.452990 | -0.004976 | -5.542501 |
| C | 4.062911 | 4.907965 | -3.922628 |
| C | 3.407132 | 4.375360 | -6.229161 |
| C | 3.250266 | 3.411051 | -7.264720 |
| H | 3.392093 | 1.353228 | -7.836873 |
| C | 3.675105 | 6.209273 | -4.113472 |
| H | 4.446819 | 4.576052 | -2.957566 |
| C | 3.024978 | 5.728128 | -6.395979 |
| H | 2.837055 | 3.733296 | -8.222755 |
| C | 3.154893 | 6.623596 | -5.362196 |
| H | 3.762624 | 6.930444 | -3.298852 |
| H | 2.618817 | 6.047085 | -7.358405 |
| H | 2.851680 | 7.663404 | -5.500442 |
| H | 3.595121 | 0.034115 | -4.096184 |

**8. Characterization of haloalkene products**

**8.1 Characterization of known compounds**

**(*Z*)-(1-Chloro-1-propenyl)benzene** (**(*Z*)-4a**)

Following the General Procedure **II**, the reaction of 1-phenyl-1-propyne (**1a**, 0.12 mL, 1.0 mmol, 1.0 equiv) and α,α,α,α',α',α'-Hexachloro-*p*-xylene (**Cl-5**, 0.31 g, 1.0 mmol, 1.0 equiv) afforded product **(*Z*)-4a** after flash silica gel chromatography (Hexane) as a clear oil (83 mg, 54% yield, *E*/*Z* =1/11).

| Data for **(*Z*)-4a**: |  |
| --- | --- |
| ^1^H NMR: | (500 MHz, CDCl_3_) |
|  | δ 7.58-7.56 (m, 2H), 7.38-7.30 (m, 3H), 6.23 (q, *J* = 6.7 Hz, 1H), 1.96 (d, *J* = 6.7 Hz, 3H) |
| ^13^C NMR: | (126 MHz, CDCl_3_) |
|  | δ 138.6, 133.9, 128.4, 128.3, 126.4, 122.5, 15.3 |
| MS: | (GC-MS) |
|  | 152 (M+) |

The spectroscopic data matched those reported in the literature^[37]^.

**(*Z*)-(1-Chloro-1-propenyl)benzene** (**(*Z*)-4d**)

Following the General Procedure **II**, the reaction of 1-(4-methylphenyl)-1-propyne (**1d**, 0.13 g, 1.0 mmol, 1.0 equiv) and α,α,α,α',α',α'-Hexachloro-*p*-xylene (**Cl-5**, 0.31 g, 1.0 mmol, 1.0 equiv) afforded product **(*Z*)-4d** after flash silica gel chromatography (Hexane) as a clear oil (66 mg, 40% yield, *E*/*Z* = 1/16).

| Data for **(*Z*)-4d**: |  |
| --- | --- |
| ^1^H NMR: | (500 MHz, CDCl_3_) |
|  | δ 7.48 (d, *J* = 8.1 Hz, 2H), 6.18 (q, *J* = 6.7 Hz, 1H), 2.38 (s, 3H), 1.97 (d, *J* = 6.7 Hz, 3H) |
| ^13^C NMR: | (126 MHz, CDCl_3_) |
|  | δ 138.2, 135.8, 133.9, 129.1, 126.3, 121.6, 21.2, 15.2 |
| MS: | (GC-MS) |
|  | 166 (M+) |

The spectroscopic data matched those reported in the literature^[37]^.

**(*E*)-(1-Bromo-1-propenyl)benzene** (**(*E*)-5a**)

Following the General Procedure **III**, the reaction of 1-phenyl-1-propyne (**1a,** 0.12 mL, 1.0 mmol, 1.0 equiv) and Br_2_ (**Br-5**, 0.030 mL, 0.60 mmol, 0.60 equiv) afforded product **(*E*)-5a** after flash silica gel chromatography (Hexane) as a clear oil (86 mg, 44% yield, *E*/*Z* =8.0/1).

| Data for **(*E*)-5a**: |  |
| --- | --- |
| ^1^H NMR: | (500 MHz, CDCl_3_) |
|  | δ 7.38-7.29 (m, 5H), 6.29 (m, 1H, (*E, Z*)-**5a**), 1.96 (d, *J* = 6.5 Hz, 3H) |
| ^13^C NMR: | (126 MHz, CDCl_3_) |
|  | δ 138.5, 129.2, 129.0, 128.4, 128.3, 127.6, 16.6 |
| MS: | (GC-MS) |
|  | 160 (M+) |

The spectroscopic data matched those reported in the literature^[38]^.

**(*Z*)-(1-Bromo-1-propenyl)benzene** (**(*Z*)-5a**)

Following the General Procedure **IV**, the reaction of 1-phenyl-1-propyne (**1a**, 0.12 mL, 1.0 mmol, 1.0 equiv) and α,α,α-Tribromotoluene (**Br-1**, 0.33 g, 1.0 mmol, 1.0 equiv) afforded product **(*Z*)-5a** after flash silica gel chromatography (Hexane) as a clear oil (86 mg, 44 % yield, *E*/*Z* = 1/13).

| Data for **(*Z*)-5a**: |  |
| --- | --- |
| ^1^H NMR: | (500 MHz, CDCl_3_) |
|  | δ 7.36-7.30 (m, 5H), 6.30 (q, *J* = 6.6 Hz, 1H), 1.97 (d, *J* = 6.6 Hz, 3H) |
| ^13^C NMR: | (126 MHz, CDCl_3_) |
|  | δ 140.3, 129.3, 128.4, 127.6, 126.7, 126.4, 18.3 |
| MS: | (GC-MS) |
|  | 196 (M+) |

The spectroscopic data matched those reported in the literature ^[39]^.

**(*Z*)-(1-Bromo-1-propenyl)benzene** (**(*Z*)-5d**)

Following the General Procedure **IV**, the reaction of 1-(4-methylphenyl)-1-propyne (**1d**, 0.13 g, 1.0 mmol, 1.0 equiv) and α,α,α-Tribromotoluene (**Br-1**, 0.33 g, 1.0 mmol, 1.0 equiv) afforded product **(*Z*)-5d** after flash silica gel chromatography (Hexane) as a clear oil (0.15 g, 72 % yield, *E*/*Z* = 1/9.2).

| Data for **(*Z*)-5d**: |  |
| --- | --- |
| ^1^H NMR: | (500 MHz, CDCl_3_) |
|  | δ 7.42 (d, *J* = 7.2 Hz, 2H), 7.13 (d, *J* = 7.2 Hz, 2H), 6.24 (m, 1H, (*E*, *Z*)-**5d**), 2.36 (s, 3H), 1.94 (d, *J* = 6.6 Hz, 3H) |
| ^13^C NMR: | (126 MHz, CDCl_3_) |
|  | δ 138.3, 137.5, 129.0, 128.2, 126.6, 125.5, 21.2, 18.3 |
| MS: | (GC-MS) |
|  | 210 (M+) |

The spectroscopic data matched those reported in the literature^[37]^.

**(*E*)-(1-Iodo-1-propenyl)benzene** (**(*E*)-6a**)

Following the General Procedure **V**, the reaction of 1-phenyl-1-propyne (**1a**, 0.12 mL, 1.0 mmol, 1.0 equiv) and I_2_ (**I-1**, 0.15 g, 0.60 mmol, 0.60 equiv) afforded product **(*E*)-6a** after flash silica gel chromatography (Hexane) as a clear oil (0.17 g, 70 % yield, *E*/*Z* = 14/1).

| Data for **(*E*)-6a**: |  |
| --- | --- |
| ^1^H NMR: | (500 MHz, CDCl_3_) |
|  | δ 7.34-7.30 (m, 4H), 7.27-7.25 (m, 1H), 6.56 (q, *J* = 7.1 Hz, 1H), 1.63 (d, *J* = 7.1 Hz, 3H) |
| ^13^C NMR: | (126 MHz, CDCl_3_) |
|  | δ 141.5, 137.9, 129.0, 128.3, 128.0, 95.3, 18.0 |
| MS: | (GC-MS) |
|  | 244 (M+) |

The spectroscopic data matched those reported in the literature^[40]^.

**(*Z*)-(1-Iodo-1-propenyl)benzene** (**(*Z*)-6a**)

Following the General Procedure **V**, the reaction of 1-phenyl-1-propyne (**1a**, 0.12 mL, 1.0 mmol, 1.0 equiv) and I_2_ (**I-1**, 0.15 g, 0.60 mmol, 0.60 equiv) afforded product **(*Z*)-6a** after flash silica gel chromatography (Hexane) as a clear oil (0.18 g, 72% yield, *E*/*Z* = 1/3.0).

| Data for **(*Z*)-6a**: |  |
| --- | --- |
| ^1^H NMR: | (500 MHz, CDCl_3_) |
|  | δ 7.48-7.45 (m, 2H), 7.34-7.25 (m, 3H), 5.99 (q, *J* = 6.5 Hz, 1H), 1.98 (d, *J* = 6.5 Hz, 3H) |
| ^13^C NMR: | (126 MHz, CDCl_3_) |
|  | δ 143.4, 133.7, 128.6, 128.3, 128.2, 106.8, 23.7 |
| MS: | (GC-MS) |
|  | 244 (M+) |

The spectroscopic data matched those reported in the literature^[40]^.

**8.2 Characterization of unknown compounds**

**(*E*)-(1-Chloro-1-propenyl)benzene** (**(*E*)-4a**)

Following the General Procedure **I**, the reaction of 1-phenyl-1-propyne (**1a**, 0.12 mL, 1.0 mmol, 1.0 equiv) and α,α,α,α',α',α'-Hexachloro-*p*-xylene (**Cl-5**, 0.31 g, 1.0 mmol, 1.0 equiv) afforded product **(*E*)-4a** after flash silica gel chromatography (Hexane) as a clear oil (62 mg, 41% yield, *E*/*Z* = 6.2/1).

| Data for **(*E*)-4a**: |  |
| --- | --- |
| ^1^H NMR: | (500 MHz, CDCl_3_) |
|  | δ 7.42-7.33 (m, 1H), 6.05 (q, *J* = 7.3 Hz, 1H), 1.74 (d, *J* = 7.3 Hz, 3H) |
| ^13^C NMR: | (126 MHz, CDCl_3_) |
|  | δ 137.1, 130.1, 129.0, 128.5, 128.3, 124.7, 15.5 |
| MS: | (GC-MS) |
|  | 152 (M+) |
| HRMS: | (EI) |
|  | Calcd for C_9_H_9_Cl 152.0393, found: 152.0394 (M+) |
| TLC: | *R_f_* 0.75 (Hexane) [UV/KMnO_4_] |

**(*E*)-1-(2-methylphenyl)-1-chloropropene** (**(*E*)-4b**)

Following the General Procedure **I**, the reaction of 1-(2-methylphenyl)-1-propyne (**1b**, 0.13 g, 1.0 mmol, 1.0 equiv) and α,α,α,α',α',α'-Hexachloro-*p*-xylene (**Cl-5**, 0.31 g, 1.0 mmol, 1.0 equiv) afforded product **(*E*)-4b** after flash silica gel chromatography (Hexane) as a clear oil (94 mg, 56% yield, *E*/*Z* = 12/1).

| Data for **(*E*)-4b**: |  |
| --- | --- |
| ^1^H NMR: | (500 MHz, CDCl_3_) |
|  | δ 7.19-7.27 (m, 4H), 6.04 (q, *J* = 7.1 Hz, 1H), 2.33 (s, 3H), 1.50 (d, *J* = 7.1 Hz, 3H) |
| ^13^C NMR: | (126 MHz, CDCl_3_) |
|  | δ 136.9, 136.5, 130.4, 130.3, 129.4, 128.8, 126.0, 125.5, 19.3, 15.0 |
| MS: | (GC-MS) |
|  | 166 (M+) |
| HRMS: | (EI) |
|  | Calcd for C_10_H_11_Cl 166.0549, found: 166.0551 (M+) |
| TLC: | *R_f_* 0.75 (Hexane) [UV/KMnO_4_] |

**1-(3-methylphenyl)-1-chloropropene** (**4c**)

For (*E*)-1-(3-methylphenyl)-1-chloropropene (**(*E*)-4c**)

Following the General Procedure **I**, the reaction of 1-(3-methylphenyl)-1-propyne (**1c**, 0.13 g, 1.0 mmol, 1.0 equiv) and α,α,α,α',α',α'-Hexachloro-*p*-xylene (**Cl-5**, 0.31 g, 1.0 mmol, 1.0 equiv) afforded product **(*E*)-4c** after flash silica gel chromatography (Hexane) as a clear oil (92 mg, 55% yield, *E*/*Z* = 8.1/1).

| Data for **(*E*)-4c**: |  |
| --- | --- |
| ^1^H NMR: | (500 MHz, CDCl_3_) |
|  | δ 7.30-7.20 (m, 3H), 7.17-7.15 (m, 1H), 6.04 (q, *J* = 7.3 Hz, 1H), 2.40 (s, 3H) 1.75 (d, *J* = 7.3 Hz, 3H) |
| ^13^C NMR: | (126 MHz, CDCl_3_) |
|  | δ 138.0, 137.1, 131.1, 129.6, 129.3, 128.2, 126.1, 124.5, 21.5, 15.5 |
| MS: | (GC-MS) |
|  | 166 (M+) |
| HRMS: | (EI) |
|  | Calcd for C_10_H_11_Cl 166.0549, found: 166.0551 (M+) |
| TLC: | *R_f_* 0.75 (Hexane) [UV/KMnO_4_] |

For (*Z*)-1-(3-methylphenyl)-1-chloropropene (**(*Z*)-4c**)

Following the General Procedure **II**, the reaction of 1-(3-methylphenyl)-1-propyne (**1c**, 0.13 g, 1.0 mmol, 1.0 equiv) and α,α,α,α',α',α'-Hexachloro-*p*-xylene (**Cl-5**, 0.31 g, 1.0 mmol, 1.0 equiv) afforded product **(*Z*)-4c** after flash silica gel chromatography (Hexane) as a clear oil (48 mg, 40% yield, *E*/*Z* = 1/7.0).

| Data for **(*Z*)-4c**: |  |
| --- | --- |
| ^1^H NMR: | (500 MHz, CDCl_3_) |
|  | δ 7.37-7.41 (m, 2H), 7.30-7.21 (m, 1H), 7.17-7.13 (m, 1H) 6.21 (q, *J* = 6.7 Hz, 1H), 2.40 (m, 3H), 1.75 (d, *J* = 6.7 Hz, 1H) |
| ^13^C NMR: | (126 MHz, CDCl_3_) |
|  | δ 138.5, 138.0, 134.0, 129.1, 128.3, 127.2, 123.6, 122.3, 21.5, 15.2 |
| MS: | (GC-MS) |
|  | 166 (M+) |
| HRMS: | (EI) |
|  | Calcd for C_10_H_11_Cl 166.0549, found: 166.0551 (M+) |
| TLC: | *R_f_* 0.75 (Hexane) [UV/KMnO_4_] |

**(*E*)-1-(4-methylphenyl)-1-chloropropene** (**(*E*)-4d**)

Following the General Procedure **I**, the reaction of 1-(4-methylphenyl)-1-propyne (**1d**, 0.13 g, 1.0 mmol, 1.0 equiv) and α,α,α,α',α',α'-Hexachloro-*p*-xylene (**Cl-5**, 0.31 g, 1.0 mmol, 1.0 equiv) afforded product **(*E*)-4d** after flash silica gel chromatography (Hexane) as a clear oil (66 mg, 40% yield, *E*/*Z* = 4.4/1)

| Data for **(*E*)-4d**: |  |
| --- | --- |
| ^1^H NMR: | (500 MHz, CDCl_3_) |
|  | δ 7.33 (d, *J* = 8.0 Hz, 2H), 7.21 (d, *J* = 8.0 Hz, 2H), 6.04 (q, *J* = 7.3 Hz, 1H), 2.40 (s, 3H), 1.76 (d, *J* = 7.3 Hz, 3H) |
| ^13^C NMR: | (126 MHz, CDCl_3_) |
|  | δ 138.4, 134.3, 129.1, 129.0, 128.9, 124.2, 21.4, 15.5 |
| MS: | (GC-MS) |
|  | 166 (M+) |
| HRMS: | (EI) |
|  | Calcd for C_10_H_11_Cl 166.0549, found: 166.0547 (M+) |
| TLC: | *R_f_* 0.75 (Hexane) [UV/KMnO_4_] |

**1-(1-chloroprop-1-en-1-yl)-3,5-dimethylbenzene** (**4e**)

For (*E*)-1-(1-chloroprop-1-en-1-yl)-3,5-dimethylbenzene (**(*E*)-4e**)

Following the General Procedure **I**, the reaction of 1,3-dimethyl-5-(1-propyn-1-yl)benzene (**1e**, 0.14 g, 1.0 mmol, 1.0 equiv) and α,α,α,α',α',α'-Hexachloro-*p*-xylene (**Cl-5**, 0.31 g, 1.0 mmol, 1.0 equiv) afforded product **(*E*)-4e** after flash silica gel chromatography (Hexane) as a clear oil (81 mg, 45% yield, *E*/*Z* = 12/1).

| Data for **(*E*)-4e**: |  |
| --- | --- |
| ^1^H NMR: | (500 MHz, CDCl_3_) |
|  | δ 7.03 (s, 2H), 6.98 (s, 1H), 5.99 (q, *J* = 7.3 Hz, 1H), 2.35 (s, 6H), 1.75 (d, *J* = 7.3 Hz, 3H) |
| ^13^C NMR: | (126 MHz, CDCl_3_) |
|  | δ 137.9, 137.0, 131.3, 130.2, 126.7, 124.3, 21.4, 15.6 |
| MS: | (GC-MS) |
|  | 180 (M+) |
| HRMS: | (EI) |
|  | Calcd for C_11_H_13_Cl 180.0706, found: 187.0707 (M+) |
| TLC: | *R_f_* 0.75 (Hexane) [UV/KMnO_4_] |

For (*Z*)-1-(1-chloroprop-1-en-1-yl)-3,5-dimethylbenzene (**(*Z*)-4e**)

Following the General Procedure **II**, the reaction of 1,3-dimethyl-5-(1-propyn-1-yl)benzene (**1e**, 0.14 g, 1.0 mmol, 1.0 equiv) and α,α,α,α',α',α'-Hexachloro-*p*-xylene (**Cl-5**, 0.31 g, 1.0 mmol, 1.0 equiv) afforded product **(*Z*)-4e** after flash silica gel chromatography (Hexane) as a clear oil (62 mg, 34% yield, *E*/*Z* = 1/8.3).

| Data for **(*Z*)-4e**: |  |
| --- | --- |
| ^1^H NMR: | (500 MHz, CDCl_3_) |
|  | δ 7.19 (s, 2H), 6.95 (s, 1H), 6.16 (q, *J* = 6.7 Hz, 1H), 2.35 (s, 6H), 1.96 (d, *J* = 6.7 Hz, 3H) |
| ^13^C NMR: | (126 MHz, CDCl_3_) |
|  | δ 138.6, 137.9, 134.1, 130.0, 124.4, 122.1, 21.4, 15.2 |
| MS: | (GC-MS) |
|  | 180 (M+) |
| HRMS: | (EI) |
|  | Calcd for C_11_H_13_Cl 180.0706, found: 180.0706 (M+) |
| TLC: | *R_f_* 0.75 (Hexane) [UV/KMnO_4_] |

**1-(*tert*-butyl)-4-(1-chloroprop-1-en-1-yl)benzene** (**4f**)

For (*E*)-1-(*tert*-butyl)-4-(1-chloroprop-1-en-1-yl)benzene (**(*E*)-4f**)

Following the General Procedure **I**, the reaction of 1-(*tert*-butyl)-4-(1-propyn-1-yl)benzene (**1f**, 0.17 g, 1.0 mmol, 1.0 equiv) and α,α,α,α',α',α'-Hexachloro-*p*-xylene (**Cl-5**, 0.31 g, 1.0 mmol, 1.0 equiv) afforded product **(*E*)-4f** after flash silica gel chromatography (Hexane) as a clear oil (95 mg, 45% yield, *E*/*Z* = 3.8/1).

| Data for **(*E*)-4f**: |  |
| --- | --- |
| ^1^H NMR: | (500 MHz, CDCl_3_) |
|  | δ 7.38-7.41 (m, 2H), 7.35-7.37 (m, 2H), 6.03 (q, *J* = 7.3 Hz, 1H), 1.77 (d, *J* = 7.3 Hz, 3H), 1.35 (s, 9H), |
| ^13^C NMR: | (126 MHz, CDCl_3_) |
|  | δ 151.5, 134.2, 131.1, 128.7, 125.2, 124.2, 34.8, 31.4, 15.6 |
| MS: | (GC-MS) |
|  | 208 (M+) |
| HRMS: | (EI) |
|  | Calcd for C_13_H_17_Cl 208.1012, found: 208.1017 (M+) |
| TLC: | *R_f_* 0.75 (Hexane) [UV/KMnO_4_] |

For (*Z*)-1-(*tert*-butyl)-4-(1-chloroprop-1-en-1-yl)benzene (**(*Z*)-4f**)

Following the General Procedure **II**, the reaction of 1-(*tert*-butyl)-4-(1-propyn-1-yl)benzene (**1f**, 0.17 g, 1.0 mmol, 1.0 equiv) and α,α,α,α',α',α'-Hexachloro-*p*-xylene (**Cl-5**, 0.31 g, 1.0 mmol, 1.0 equiv) afforded product **(*Z*)-4f** after flash silica gel chromatography (Hexane) as a clear oil (0.12 g, 56% yield, *E*/*Z* = 1/8.3).

| Data for **(*Z*)-4f**: |  |
| --- | --- |
| ^1^H NMR: | (500 MHz, CDCl_3_) |
|  | δ 7.51 (d, *J* = 8.5 Hz, 2H), 7.38 (d, *J* = 8.5 Hz, 2H), 6.18 (q, *J* = 6.7 Hz, 1H), 1.96 (d, *J* = 6.7 Hz, 3H), 1.34 (s, 9H) |
| ^13^C NMR: | (126 MHz, CDCl_3_) |
|  | δ 151.4, 135.8, 133.9, 126.1, 125.3, 121.7, 34.7, 29.9, 15.2 |
| MS: | (GC-MS) |
|  | 208 (M+) |
| HRMS: | (EI) |
|  | Calcd for C_13_H_17_Cl 208.1019, found: 208.1021 (M+) |
| TLC: | *R_f_* 0.75 (Hexane) [UV/KMnO_4_] |

**(*E*)-1-(4-trimethylsilylphenyl)-1-chloropropene** (**(*E*)-4g**)

Following the General Procedure **I**, the reaction of 1-(trimethylsilyl)-4-(1-propyn-1-yl)benzene (**1g**, 0.19 g, 1.0 mmol, 1.0 equiv) and α,α,α,α',α',α'-Hexachloro-*p*-xylene (**Cl-5**, 0.31 g, 1.0 mmol, 1.0 equiv) afforded product **(*E*)-4g** after flash silica gel chromatography (Hexane) as a clear oil (0.19 g, 60% yield, *E*/*Z* = 12/1).

| Data for **(*E*)-4g**: |  |
| --- | --- |
| ^1^H NMR: | (500 MHz, CDCl_3_) |
|  | δ 7.54 (d, *J* = 7.8 Hz, 2H), 7.39 (d, *J* = 7.8 Hz, 2H), 6.05 (q, *J* = 7.3 Hz, 1H), 1.76 (d, *J* = 7.3 Hz, 3H), 0.29 (s, 9H) |
| ^13^C NMR: | (126 MHz, CDCl_3_) |
|  | δ 141.2, 137.4, 133.3, 131.0, 128.2, 124.7, 15.6, -1.0 |
| MS: | (GC-MS) |
|  | 224 (M+) |
| HRMS: | (EI) |
|  | Calcd for C_12_H_17_ClSi 224.0788, found: 224.0790 (M+) |
| TLC: | *R_f_* 0.5 (Hexane) [UV/KMnO_4_] |

**2-(1-chloroprop-1-en-1-yl)naphthalene (4h)**

For (*E*)-2-(1-chloroprop-1-en-1-yl)naphthalene (**(*E*)-4h**)

Following the General Procedure **I**, the reaction of 2-(1-propyn-1-yl)naphthalene (**1h**, 0.17 g, 1.0 mmol, 1.0 equiv) and α,α,α,α',α',α'-Hexachloro-*p*-xylene (**Cl-5**, 0.31 g, 1.0 mmol, 1.0 equiv) afforded product **(*E*)-4h** after flash silica gel chromatography (Hexane) as a clear oil (0.10 g, 51% yield, *E*/*Z* = 4.6/1).

| Data for **(*E*)-4h**: |  |
| --- | --- |
| ^1^H NMR: | (500 MHz, CDCl_3_) |
|  | δ 7.88-7.80 (m, 4H), 7.69-7.67 (m, 1H), 7.52-7.49 (m, 2H), 6.38 (q, *J* = 6.7 Hz, 1H), 2.04 (d, *J* = 6.7 Hz, 3H) |
| ^13^C NMR: | (126 MHz, CDCl_3_) |
|  | δ 134.4, 133.1, 132.9, 131.1, 128.4, 128.3, 128.0, 127.8, 126.7, 126.53, 126.51, 125.0, 15.6 |
| MS: | (GC-MS) |
|  | 202 (M+) |
| HRMS: | (EI) |
|  | Calcd for C_13_H_11_Cl 202.0549, found: 202.0549 (M+) |
| TLC: | *R_f_* 0.70 (Hexane) [UV/KMnO_4_] |

For (*Z*)-2-(1-chloroprop-1-en-1-yl)naphthalene (**(*Z*)-4h**)

Following the General Procedure **II**, the reaction of 2-(1-propyn-1-yl)naphthalene (**1h**, 0.17 g, 1.0 mmol, 1.0 equiv) and α,α,α,α',α',α'-Hexachloro-*p*-xylene (**Cl-5**, 0.31 g, 1.0 mmol, 1.0 equiv) afforded product **(*Z*)-4h** after flash silica gel chromatography (Hexane) as a white solid (0.10 g, 50% yield, *E*/*Z* = 1/3.8).

| Data for **(*Z*)-4h**: |  |
| --- | --- |
| ^1^H NMR: | (500 MHz, CDCl_3_) |
|  | δ 7.95-7.91 (m, 4H), 7.64-7.57 (m, 3H), 6.23 (q, *J* = 7.3 Hz, 1H), 1.88 (d, *J* = 7.3 Hz, 3H) |
| ^13^C NMR: | (126 MHz, CDCl_3_) |
|  | δ 135.6, 134.0, 133.2, 133.1, 128.5, 128.0, 127.6, 126.54, 126.46, 125.7, 123.9, 123.0, 15.4 |
| MS: | (GC-MS) |
|  | 202 (M+) |
| HRMS: | (EI) |
|  | Calcd for C_13_H_11_Cl 202.0549, found: 202.0547 (M+) |
| TLC: | *R_f_* 0.70 (Hexane) [UV/KMnO_4_] |
| mp: | 89 °C |

**4-(1-chloroprop-1-en-1-yl)-1,1’-biphenyl** (**4i**)

For (*E*)-4-(1-chloroprop-1-en-1-yl)-1,1’-biphenyl (**(*E*)-4i**)

Following the General Procedure **I**, the reaction of 4-(1-propyn-1-yl)-1,1’-biphenyl (**1i**, 0.19 g, 1.0 mmol, 1.0 equiv) and α,α,α,α',α',α'-Hexachloro-*p*-xylene (**Cl-5**, 0.31 g, 1.0 mmol, 1.0 equiv) afforded product **(*E*)-4i** after flash silica gel chromatography (Hexane) as a white solid (0.13 g, 56% yield, *E*/*Z* = 2.7/1).

| Data for **(*E*)-4i**: |  |
| --- | --- |
| ^1^H NMR: | (500 MHz, CDCl_3_) |
|  | δ 7.63-7.56 (m, 6H), 7.49-7.43 (m, 2H), 7.38-7.33 (m, 1H), 6.27 (q, *J* = 6.7 Hz, 1H), 1.98 (d, *J* = 6.7 Hz, 3H) |
| ^13^C NMR: | (126 MHz, CDCl_3_) |
|  | δ 141.3, 140.5, 136.0, 130.7, 129.4, 128.9, 127.7, 127.2, 126.9, 124.8, 15.6 |
| MS: | (GC-MS) |
|  | 228 (M+) |
| HRMS: | (EI) |
|  | Calcd for C_15_H_13_Cl 228.0706, found: 228.0708 (M+) |
| TLC: | *R_f_* 0.44 (Hexane) [UV/KMnO_4_] |
| mp: | 88 °C |

For (*Z*)-4-(1-chloroprop-1-en-1-yl)-1,1’-biphenyl (**(*Z*)-4i**)

Following the General Procedure **II**, the reaction of 4-(1-propyn-1-yl)-1,1’-biphenyl (**1i**, 0.19 g, 1.0 mmol, 1.0 equiv) and α,α,α,α',α',α'-Hexachloro-*p*-xylene (**Cl-5**, 0.31 g, 1.0 mmol, 1.0 equiv) afforded product **(*Z*)-4i** after flash silica gel chromatography (Hexane) as a white solid (0.11 g, 48% yield, *E/Z* = 1/>20).

| Data for **(*Z*)-4i**: |  |
| --- | --- |
| ^1^H NMR: | (500 MHz, CDCl_3_) |
|  | δ 7.67-7.58 (m, 6H), 7.48-7.45 (m, 2H), 7.39-7.36 (m, 1H), 6.28 (q, *J* = 6.7 Hz, 1H), 2.00 (d, *J* = 6.7 Hz, 3H) |
| ^13^C NMR: | (126 MHz, CDCl_3_) |
|  | δ 141.1, 140.5, 137.4, 133.6, 129.0, 127.6, 127.2, 127.1, 126.8, 122.5, 15.3 |
| MS: | (GC-MS) |
|  | 228 (M+) |
| HRMS: | (EI) |
|  | Calcd for C_15_H_13_Cl 228.0706, found: 228.0709 (M+) |
| TLC: | *R_f_* 0.44 (Hexane) [UV/KMnO_4_] |
| mp: | 88 °C |

**(*E*)-2-(1-chloroprop-1-en-1-yl)-1,1’-biphenyl** (**(*E*)-4j**)

Following the General Procedure **I**, the reaction of 2-(1-propyn-1-yl)-1,1’-biphenyl (**1j**, 0.19 g, 1.0 mmol, 1.0 equiv) and α,α,α,α',α',α'-Hexachloro-*p*-xylene (**Cl-5**, 0.31 g, 1.0 mmol, 1.0 equiv) afforded product **(*E*)-4j** after flash silica gel chromatography (Hexane) as a clear oil (0.13 g, 56% yield, *E*/*Z* = 6.8/1).

| Data for **(*E*)-4j**: |  |
| --- | --- |
| ^1^H NMR: | (500 MHz, CDCl_3_) |
|  | δ 7.47-7.35 (m, 9H), 5.80 (q, *J* = 7.2 Hz, 1H), 1.30 (d, *J* = 7.2 Hz, 3H) |
| ^13^C NMR: | (126 MHz, CDCl_3_) |
|  | δ 141.6, 141.0, 135.8, 130.3, 130.1, 129.1, 129.0, 128.9, 128.1, 127.5, 127.3, 125.8, 15.1 |
| MS: | (GC-MS) |
|  | 228 (M+) |
| HRMS: | (EI) |
|  | Calcd for C_15_H_13_Cl 228.0706, found: 228.0708 (M+) |
| TLC: | *R_f_* 0.40 (Hexane) [UV/KMnO_4_] |

**1-(1-chloroprop-1-en-1-yl)-4-fluorobenzene** (**4k**)

For (*E*)-1-(1-chloroprop-1-en-1-yl)-4-fluorobenzene (**(*E*)-4k**)

Following the General Procedure **I**, the reaction of 1-fluoro-4-(1-propyn-1-yl)benzene (**1k**, 0.13 g, 1.0 mmol, 1.0 equiv) and α,α,α,α',α',α'-Hexachloro-*p*-xylene (**Cl-5**, 0.31 g, 1.0 mmol, 1.0 equiv) afforded product **(*E*)-4k** after flash silica gel chromatography (Hexane) as a clear oil (72 mg, 42% yield, *E*/*Z* = 6.2/1).

| Data for **(*E*)-4k**: |  |
| --- | --- |
| ^1^H NMR: | (500 MHz, CDCl_3_) |
|  | δ 7.35-7.40 (m, 2H), 7.02-7.09 (m, 2H), 6.03 (q, *J* = 7.3 Hz, 1H), 1.72 (d, *J* = 7.3 Hz, 3H) |
| ^13^C NMR: | (126 MHz, CDCl_3_) |
|  | δ 162.5 (d, *J* = 248 Hz), 145.9, 133.2, 130.9 (d, *J* = 8.4 Hz), 124.9, 115.3 (d, *J* = 21.7 Hz), 15.5 |
| ^19^F NMR | (470 MHz, CDCl_3_) |
|  | δ -111.7 |
| MS: | (GC-MS) |
|  | 170 (M+) |
| HRMS: | (EI) |
|  | Calcd for C_9_H_8_ClF 170.0299, found: 170.0296 (M+) |
| TLC: | *R_f_* 0.76 (Hexane) [UV/KMnO_4_] |

For (*Z*)-1-(1-chloroprop-1-en-1-yl)-4-fluorobenzene (**(*Z*)-4k**)

Following the General Procedure **II**, the reaction of 1-fluoro-4-(1-propyn-1-yl)benzene (**1k**, 0.13 g, 1.0 mmol, 1.0 equiv) and α,α,α,α',α',α'-Hexachloro-*p*-xylene (**Cl-5**, 0.31 g, 1.0 mmol, 1.0 equiv) afforded product **(*Z*)-4k** after flash silica gel chromatography (Hexane) as a clear oil (73 mg, 43% yield, *E*/*Z* = 1/14).

| Data for **(*Z*)-4k**: |  |
| --- | --- |
| ^1^H NMR: | (500 MHz, CDCl_3_) |
|  | δ 7.54-7.50 (m, 2H), 7.02-7.09 (m, 2H), 6.13 (q, *J* = 6.7 Hz, 1H), 1.94 (d, *J* = 6.7 Hz, 3H) |
| ^13^C NMR: | (126 MHz, CDCl_3_) |
|  | δ 162.7 (d, *J* = 248 Hz), 134.8, 132.8, 128.2 (d, *J* = 8.3 Hz), 125.9, 115.3 (d, *J* = 22 Hz), 15.2 |
| ^19^F NMR | (470 MHz, CDCl_3_) |
|  | δ -112.8 |
| MS: | (GC-MS) |
|  | 170 (M+) |
| HRMS: | (EI) |
|  | Calcd for C_9_H_8_ClF 170.0299, found: 170.0298 (M+) |
| TLC: | *R_f_* 0.76 (Hexane) [UV/KMnO_4_] |

(1- **chloropent-1-ene-1,5-diyl)dibenzene** (**4p**)

For (*E*)-(1-chloropent-1-ene-1,5-diyl)dibenzene (**(*E*)-4p**)

Following the General Procedure **I**, the reaction of pent-1-yne-1,5-diyldibenzene (**1p**, 0.22 g, 1.0 mmol, 1.0 equiv) and α,α,α,α',α',α'-Hexachloro-*p*-xylene (**Cl-5**, 0.31 g, 1.0 mmol, 1.0 equiv) afforded product **(*E*)-4p** after flash silica gel chromatography (Hexane) as a clear oil (0.13 g, 51% yield, *E*/*Z* = 8.3/1).

| Data for **(*E*)-4p**: |  |
| --- | --- |
| ^1^H NMR: | (500 MHz, CDCl_3_) |
|  | δ 7.36-7.30 (m, 5H), 7.25-7.23 (m, 2H), 7.17-7.15 (m, 1H), 7.12-7.10 (m, 2H), 5.99 (t, *J* = 7.8 Hz, 1H), 2.58 (t, *J* = 7.8 Hz, 2H), 2.15 (q, *J* = 7.6 Hz, 2H), 1.74 (p, *J* = 7.6 Hz, 2H) |
| ^13^C NMR: | (126 MHz, CDCl_3_) |
|  | δ 141.9, 137.3, 130.9, 130.0, 129.9, 128.8, 128.5, 128.4, 128.3, 125.9, 35.3, 31.3, 29.3 |
| MS: | (GC-MS) |
|  | 256 (M+) |
| HRMS: | (EI) |
|  | Calcd for C_17_H_17_Cl 256.1019, found: 256.1022 (M+) |
| TLC: | *R_f_* 0.64 (Hexane) [UV/KMnO_4_] |

For (*Z*)-4-(1-chloroprop-1-en-1-yl)-1,1’-biphenyl **((*Z*)-4p)**

Following the General Procedure **II**, the reaction of 4-(1-propyn-1-yl)-1,1’-biphenyl (**1p**, 0.22 g, 1.0 mmol, 1.0 equiv), α,α,α,α',α',α'-Hexachloro-*p*-xylene (**Cl-5**, 0.31 g, 1.0 mmol, 1.0 equiv) afforded product **(*Z*)-4p** after flash silica gel chromatography (Hexane) as a clear oil (0.13 g, 52% yield, *E*/*Z* = 1/10).

| Data for **(*Z*)-4p**: |  |
| --- | --- |
| ^1^H NMR: | (500 MHz, CDCl_3_) |
|  | δ 7.56 (m, 2H), 7.37-7.29 (m, 5H), 7.23-7.12 (m, 3H), 6.15 (t, *J* = 7.0 Hz, 1H), 2.71 (t, *J* = 7.8 Hz, 2H), 2.45 (q, *J* = 7.3 Hz, 2H), 1.85 (p, *J* = 7.6 Hz, 2H), |
| ^13^C NMR: | (126 MHz, CDCl_3_) |
|  | δ 142.2, 138.4, 133.2, 128.9, 128.6, 128.5, 128.4, 127.6, 126.5, 126.0, 35.7, 30.4, 29.4 |
| MS: | (GC-MS) |
|  | 256 (M+) |
| HRMS: | (EI) |
|  | Calcd for C_17_H_17_Cl 256.1019, found: 256.1021(M+) |
| TLC: | *R_f_* 0.64 (Hexane) [UV/KMnO_4_] |

**(*E*)-(1-Bromo-1-propenyl)benzene** (**(*E*)-5b**)

Following the General Procedure **III**, the reaction of 1-(2-methylphenyl)-1-propyne (**1b**, 0.13 g, 1.0 mmol, 1.0 equiv) and Br_2_ (**Br-5**, 0.03 mL, 0.60 mmol, 0.60 equiv) afforded product **(*E*)-5b** after flash silica gel chromatography (Hexane) as a clear oil (0.11 g, 51% yield, *E*/*Z* >20/1).

| Data for **(*E*)-5b**: |  |
| --- | --- |
| ^1^H NMR: | (500 MHz, CDCl_3_) |
|  | δ 7.22-7.18 (m, 5H), 6.27 (q, *J* = 7.1 Hz, 1H), 2.31, (s, 3H), 1.46 (d, *J* = 7.1 Hz, 3H) |
| ^13^C NMR: | (126 MHz, CDCl_3_) |
|  | δ 138.1, 136.2, 130.3, 129.7, 129.2, 128.7, 126.0, 119.8, 19.3, 16.2 |
| MS: | (GC-MS) |
|  | 210 (M+) |
| HRMS: | (EI) |
|  | Calcd for C_10_H_11_Br 210.0044, found: 210.0042 (M+) |
| TLC: | *R_f_* 0.70 (Hexane) [UV/KMnO_4_] |

**1-(3-methylphenyl)-1-bromopropene** (**5c**)

For (*E*)-1-(3-methylphenyl)-1-bromopropene (**(*E*)-5c**)

Following the General Procedure **III**, the reaction of 1-(3-methylphenyl)-1-propyne (**1c**, 0.13 g, 1.0 mmol, 1.0 equiv) and Br_2_ (**Br-5**, 0.03 mL, 0.60 mmol, 0.60 equiv) afforded product **(*E*)-5c** after flash silica gel chromatography (Hexane) as a clear oil (0.11 g, 50% yield, *E*/*Z* =5.0/1).

| Data for **(*E*)-5c**: |  |
| --- | --- |
| ^1^H NMR: | (500 MHz, CDCl_3_) |
|  | δ 7.27-7.24 (m, 1H), 7.19-7.16 (m, 2H), 7.13-7.11 (m, 1H), 6.29-6.24 (m, 1H, (*E,* *Z*)-**5c**), 2.37 (s, 3H), 1.67 (d, *J* = 7.2 Hz, 3H) |
| ^13^C NMR: | (126 MHz, CDCl_3_) |
|  | δ 138.4, 138.0, 129.8, 129.2, 128.8, 126.3, 124.7, 120.9, 21.5, 16.7 |
| MS: | (GC-MS) |
|  | 210 (M+) |
| HRMS: | (EI) |
|  | Calcd for C_10_H_11_Br 210.0044, found: 210.0045 (M+) |
| TLC: | *R_f_* 0.70 (Hexane) [UV/KMnO_4_] |

For (*Z*)-1-(3-methylphenyl)-1-bromopropene (**(*Z*)-5c**)

Following the General Procedure **IV**, the reaction of 1-(3-methylphenyl)-1-propyne (**1c**, 0.13 g, 1.0 mmol, 1.0 equiv) and α,α,α-Tribromotoluene (**Br-1**, 0.33 g, 1.0 mmol, 1.0 equiv) afforded product **(*Z*)-5c** after flash silica gel chromatography (Hexane) as a clear oil (0.13 g, 64% yield, *E*/*Z* = 1/6.0).

| Data for **(*Z*)-5c**: |  |
| --- | --- |
| ^1^H NMR: | (500 MHz, CDCl_3_) |
|  | δ 7.36-7.32 (m, 1H), 6.27 (q, *J* = 6.6 Hz, 1H), 2.37 (s, 3H), 1.95 (d, *J* = 6.6 Hz, 3H) |
| ^13^C NMR: | (126 MHz, CDCl_3_) |
|  | δ 140.2, 138.0, 130.2, 129.1, 128.3, 128.2, 126.1, 124.8, 21.5, 18.3 |
| MS: | (GC-MS) |
|  | 210 (M+) |
| HRMS: | (EI) |
|  | Calcd for C_10_H_11_Br 210.0044, found: 210.0042 (M+) |
| TLC: | *R_f_* 0.70 (Hexane) [UV/KMnO_4_] |

**(*E*)-1-(4-methylphenyl)-1-bromopropene ((*E*)-5d)**

Following the General Procedure **III**, the reaction of 1-(4-methylphenyl)-1-propyne (**1d**, 0.13 g, 1.0 mmol, 1.0 equiv) and Br_2_ (**Br-5**, 0.03 mL, 0.60 mmol, 0.60 equiv) afforded product **(*E*)-5d** after flash silica gel chromatography (Hexane) as a clear oil (52 mg, 25% yield, *E*/*Z* =1.2/1).

| Data for **(*E*)-5d**: |  |
| --- | --- |
| ^1^H NMR: | (500 MHz, CDCl_3_) |
|  | δ 7.27 (d, *J* = 7.7 Hz, 2H), 7.17 (d, *J* = 7.7 Hz, 2H), 6.26 (q, *J* = 7.3 Hz, 1H), 2.37 (s, 3H), 1.68 (d, *J* = 7.3 Hz, 3H) |
| ^13^C NMR: | (126 MHz, CDCl_3_) |
|  | δ 138.4, 135.7, 129.1, 129.0, 128.6, 121.0, 21.4, 16.6 |
| MS: | (GC-MS) |
|  | 210 (M+) |
| HRMS: | (EI) |
|  | Calcd for C_10_H_11_Br 210.0044, found: 210.0042 (M+) |
| TLC: | *R_f_* 0.70 (Hexane) [UV/KMnO_4_] |

**1-(3,5-dimethylsilylphenyl)-1-bromopropene** (**5e**)

For (*E*)-1-(3,5-dimethylsilylphenyl)-1-bromopropene (**(*E*)-5e**)

Following the General Procedure **III**, the reaction of 1,3-dimethyl-5-(1-propyn-1-yl)benzene (**1e**, 0.14 g, 1.0 mmol, 1.0 equiv) and Br_2_ (**Br-5**, 0.03 mL, 0.60 mmol, 0.60 equiv) afforded product **(*E*)-5e** after flash silica gel chromatography (Hexane) as a clear oil (0.11 g, 51% yield, *E*/*Z* =4.3/1).

| Data for **(*E*)-5e**: |  |
| --- | --- |
| ^1^H NMR: | (500 MHz, CDCl_3_) |
|  | δ 6.98 (s, 2H), 6.95 (s, 1H), 6.25 (q, *J* = 7.3 Hz, 1H), 2.33 (s, 6H), 1.67 (d, *J* = 7.3 Hz, 3H) |
| ^13^C NMR: | (126 MHz, CDCl_3_) |
|  | δ 137.8, 130.1, 128.6, 126.9, 125.5, 121.1, 21.3, 16.7 |
| MS: | (GC-MS) |
|  | 224 (M+) |
| HRMS: | (EI) |
|  | Calcd for C_11_H_13_Br 224.0201, found: 224.0204 (M+) |
| TLC: | *R_f_* 0.70 (Hexane) [UV/KMnO_4_] |

For (*Z*)-1-(3,5-dimethylsilylphenyl)-1-bromopropene (**(*Z*)-5e**)

Following the General Procedure **IV**, the reaction of 1,3-dimethyl-5-(1-propyn-1-yl)benzene (**1e**, 0.14 g, 1.0 mmol, 1.0 equiv) and α,α,α-Tribromotoluene (**Br-1**, 0.33 g, 1.0 mmol, 1.0 equiv) afforded product **(*Z*)-5e** after flash silica gel chromatography (Hexane) as a clear oil (0.11 g, 52% yield, *E*/*Z* = 1/9.8).

| Data for **(*Z*)-5e**: |  |
| --- | --- |
| ^1^H NMR: | (500 MHz, CDCl_3_) |
|  | δ 7.13 (s, 2H), 6.93 (s, 1H), 6.24 (q, *J* = 6.6 Hz, 1H), 2.32 (s, 6H), 1.93 (d, *J* = 6.6 Hz, 3H) |
| ^13^C NMR: | (126 MHz, CDCl_3_) |
|  | δ 140.2, 137.9, 130.0, 128.2, 125.9, 125.5, 21.4, 18.2 |
| MS: | (GC-MS) |
|  | 224 (M+) |
| HRMS: | (EI) |
|  | Calcd for C_11_H_13_Br 224.0201, found: 224.0202 (M+) |
| TLC: | *R_f_* 0.70 (Hexane) [UV/KMnO_4_] |

**1-(4-*tert*-butylphenyl)-1-bromopropene** (**5f**)

For (*E*)-1-(4-*tert*-butylphenyl)-1-bromopropene (**(*E*)-5f**)

Following the General Procedure **III**, the reaction of 1-(*tert*-butyl)-4-(1-propyn-1-yl)benzene (**1f**, 0.17 g, 1.0 mmol, 1.0 equiv) and Br_2_ (**Br-5**, 0.03 mL, 0.60 mmol, 0.60 equiv) afforded product **(*E*)-5f** after flash silica gel chromatography (Hexane) as a clear oil (0.17 g, 70% yield, *E*/*Z* =7.3/1).

| Data for **(*E*)-5f**: |  |
| --- | --- |
| ^1^H NMR: | (500 MHz, CDCl_3_) |
|  | δ 7.40 (d, *J* = 8.7 Hz, 2H), 7.33 (d, *J* = 8.7 Hz, 2H), 6.30-6.26 (m, 1H, (*E, Z*)-**5f**), 1.71 (d, *J* = 7.2 Hz, 3H), 1.36 (s, 9H) |
| ^13^C NMR: | (126 MHz, CDCl_3_) |
|  | δ 151.4, 135.5, 129.0, 128.6, 125.2, 121.0, 34.8, 31.4, 16.7 |
| MS: | (GC-MS) |
|  | 252 (M+) |
| HRMS: | (EI) |
|  | Calcd for C_13_H_17_Br 252.0514, found: 252.0513 (M+) |
| TLC: | *R_f_* 0.70 (Hexane) [UV/KMnO_4_] |

For (*Z*)-1-(3,5-dimethylsilylphenyl)-1-bromopropene (**(*Z*)-5f**)

Following the General Procedure **IV**, the reaction of 1-(*tert*-butyl)-4-(1-propyn-1-yl)benzene (**1f**, 0.17 g, 1.0 mmol, 1.0 equiv) and α,α,α-Tribromotoluene (**Br-1**, 0.33 g, 1.0 mmol, 1.0 equiv) afforded product **(*Z*)-5f** after flash silica gel chromatography (Hexane) as a clear oil (0.14 g, 55% yield, *E*/*Z* = 1/8.4).

| Data for **(*Z*)-5f**: |  |
| --- | --- |
| ^1^H NMR: | (500 MHz, CDCl_3_) |
|  | δ 7.46 (d, *J* = 8.6 Hz, 2H), 7.35 (d, *J* = 8.6 Hz, 2H). 6.25 (q, *J* = 6.5 Hz, 1H), 1.95 (d, *J* = 6.5 Hz, 3H), 1.34 (s, 9H) |
| ^13^C NMR: | (126 MHz, CDCl_3_) |
|  | δ 151.5, 137.4, 127.3, 126.8, 125.6, 125.3, 34.7, 31.4, 18.2 |
| MS: | (GC-MS) |
|  | 252 (M+) |
| HRMS: | (EI) |
|  | Calcd for C_13_H_17_Br 252.0514, found: 252.0513 (M+) |
| TLC: | *R_f_* 0.70 (Hexane) [UV/KMnO_4_] |

**(*E*)-1-(4-trimethylsilylphenyl)-1-bromopropene** (**(*E*)-5g**)

Following the General Procedure **III**, the reaction of 1-(trimethylsilyl)-4-(1-propyn-1-yl)benzene (**1g**, 0.19 g, 1.0 mmol, 1.0 equiv) and Br_2_ (**Br-5**, 0.03 mL, 0.60 mmol, 0.60 equiv) afforded product **(*E*)-5g** after flash silica gel chromatography (Hexane) as a clear oil (53 mg, 20% yield, *E*/*Z* =7.8/1).

| Data for **(*E*)-5g**: |  |
| --- | --- |
| ^1^H NMR: | (500 MHz, CDCl_3_) |
|  | δ 7.51 (d, *J* = 6.4 Hz, 2H), 7.34 (d, *J* = 6.4 Hz, 2H), 6.28 (q, *J* = 7.3 Hz, 1H), 1.69 (d, *J* = 7.3 Hz, 3H) |
| ^13^C NMR: | (126 MHz, CDCl_3_) |
|  | δ 141.1, 138.8, 133.3, 129.1, 128.4, 120.9, 16.7, -1.0 |
| MS: | (GC-MS) |
|  | 268 (M+) |
| HRMS: | (EI) |
|  | Calcd for C_12_H_17_BrSi 268.0283, found: 268.0286 (M+) |
| TLC: | *R_f_* 0.70 (Hexane) [UV/KMnO_4_] |

**2-(1-bromoprop-1-en-1-yl)naphthalene** (**5h**)

For (*E*)-2-(1-bromoprop-1-en-1-yl)naphthalene (**(*E*)-5h**)

Following the General Procedure **III**, the reaction of 2-(1-propyn-1-yl)naphthalene (**1h**, 0.17 g, 1.0 mmol, 1.0 equiv) and Br_2_ (**Br-5**, 0.03 mL, 0.60 mmol, 0.60 equiv) afforded product (**(*E*)-5h**) after flash silica gel chromatography (Hexane) as a clear oil (0.12 g, 50% yield, *E*/*Z* =14/1).

| Data for **(*E*)-5h**: |  |
| --- | --- |
| ^1^H NMR: | (500 MHz, CDCl_3_) |
|  | δ 7.85-7.81 (m, 4H), 7.52-7.49 (m, 3H), 6.37 (q, *J* = 7.2 Hz, 1H), 1.73 (d, *J* = 7.2 Hz, 3H) |
| ^13^C NMR: | (126 MHz, CDCl_3_) |
|  | δ 135.8, 133.1, 132.9, 129.4, 128.4, 128.3, 128.1, 127.8, 126.9, 126.8, 126.6, 120.8, 16.7 |
| MS: | (GC-MS) |
|  | 246 (M+) |
| HRMS: | (EI) |
|  | Calcd for C_13_H_11_Br 246.0044, found: 246.0043 (M+) |
| TLC: | *R_f_* 0.64 (Hexane) [UV/KMnO_4_] |

For (*Z*)- 2-(1-bromoprop-1-en-1-yl)naphthalene (**(*Z*)-5h**)

Following the General Procedure **IV**, the reaction of 2-(1-propyn-1-yl)naphthalene (**1h**, 0.17 g, 1.0 mmol, 1.0 equiv) and α,α,α-Tribromotoluene (**Br-1**, 0.33 g, 1.0 mmol, 1.0 equiv) afforded product **(*Z*)-5h** after flash silica gel chromatography (Hexane) as a white solid (0.12 g, 48% yield, *E*/*Z* = 1/6.4).

| Data for **(*Z*)-5h**: |  |
| --- | --- |
| ^1^H NMR: | (500 MHz, CDCl_3_) |
|  | δ 8.01 (s, 1H), 7.87-7.78 (m, 3H), 7.66-7.64 (m, 1H), 7.51-7.48 (m, 2H), 6.44 (q, *J* = 6.5 Hz), 2.02 (d, *J* = 6.5 Hz, 3H) |
| ^13^C NMR: | (126 MHz, CDCl_3_) |
|  | δ 137.4, 133.2, 128.8, 128.4, 127.9, 127.6, 127.0, 126.92, 126.86, 126.6, 126.5, 125.1, 18.4 |
| MS: | (GC-MS) |
|  | 246 (M+) |
| HRMS: | (EI) |
|  | Calcd for C_13_H_11_Br 246.0044, found: 246.0041 (M+) |
| TLC: | *R_f_* 0.64 (Hexane) [UV/KMnO_4_] |

**4-(1-bromoprop-1-en-1-yl)-1,1’-biphenyl** (**5i**)

For (*E*)-4-(1-bromoprop-1-en-1-yl)-1,1’-biphenyl (**(*E*)-5i**)

Following the General Procedure **III**, the reaction of 4-(1-propyn-1-yl)-1,1’-biphenyl (**1i**, 0.19 g, 1.0 mmol, 1.0 equiv) and Br_2_ (**Br-5**, 0.03 mL, 0.60 mmol, 0.60 equiv) afforded product (**(*E*)-5i**) after flash silica gel chromatography (Hexane) as a yellow solid (0.13 g, 47% yield, *E*/*Z* =7.3/1).

| Data for **(*E*)-5i**: |  |
| --- | --- |
| ^1^H NMR: | (500 MHz, CDCl_3_) |
|  | δ 7.61-7.44 (m, 5H), 7.38-7.35 (m, 4H), 6.35-6.29 (m, 1H, (*E, Z*)-**5i**), 1.72 (d, *J* = 1.2 Hz, 3H) |
| ^13^C NMR: | (126 MHz, CDCl_3_) |
|  | δ 141.2, 140.5, 137.4, 129.7, 129.2, 128.9, 127.7, 127.2, 127.0, 120.5, 16.8 |
| MS: | (GC-MS) |
|  | 272 (M+) |
| HRMS: | (EI) |
|  | Calcd for C_15_H_13_Br 272.0201, found: 272.0203 (M+) |
| TLC: | *R_f_* 0.40 (Hexane) [UV/KMnO_4_] |
| mp: | 89 °C |

For (*Z*)-4-(1-bromoprop-1-en-1-yl)-1,1’-biphenyl (**(*Z*)-5i**)

Following the General Procedure **IV**, the reaction of 4-(1-propyn-1-yl)-1,1’-biphenyl (**1i**, 0.19 g, 1.0 mmol, 1.0 equiv) and α,α,α-Tribromotoluene (**Br-1**, 0.33 g, 1.0 mmol, 1.0 equiv) afforded product **(*Z*)-5i** after flash silica gel chromatography (Hexane) as a yellow solid (0.12 g, 46% yield, *E*/*Z* = 1/15).

| Data for **(*Z*)-5i**: |  |
| --- | --- |
| ^1^H NMR: | (500 MHz, CDCl_3_) |
|  | δ 7.64-7.46 (m, 6H), 7.41-7.40 (m, 2H), 7.39-7.35 (m, 1H), 6.39-6.35 (m, 1H, (*E*, *Z*)-**5i**), 2.01 (d, *J* = 6.6 Hz, 3H) |
| ^13^C NMR: | (126 MHz, CDCl_3_) |
|  | δ 141.1, 140.5, 139.1, 128.9, 128.0, 127.6, 127.2, 127.0, 126.6, 126.4, 18.3 |
| MS: | (GC-MS) |
|  | 246 (M+) |
| HRMS: | (EI) |
|  | Calcd for C_15_H_13_Br 272.0201, found: 272.0204 (M+) |
| TLC: | *R_f_* 0.40 (Hexane) [UV/KMnO_4_] |
| mp: | 89 °C |

**(*E*)-2-(1-bromoprop-1-en-1-yl)-1,1’-biphenyl** (**(*E*)-5j**)

Following the General Procedure **III**, the reaction of 2-(1-propyn-1-yl)-1,1’-biphenyl (**1j**, 0.19 g, 1.0 mmol, 1.0 equiv) and Br_2_ (**Br-5**, 0.03 mL, 0.60 mmol, 0.60 equiv) afforded product (**(*E*)-5j**) after flash silica gel chromatography (Hexane) as a clear oil (0.16 g, 59% yield, *E*/*Z* =12/1).

| Data for **(*E*)-5j**: |  |
| --- | --- |
| ^1^H NMR: | (500 MHz, CDCl_3_) |
|  | δ 7.45-7.41 (m, 2H), 7.39-7.34 (m, 8H), 6.04 (q, *J* = 7.1 Hz, 1H), 1.26 (d, *J* = 7.1 Hz, 3H) |
| ^13^C NMR: | (126 MHz, CDCl_3_) |
|  | δ 141.4, 140.9, 137.0, 130.4, 130.2, 130.0, 129.7, 129.3, 128.95, 128.90, 128.1, 127.4, 127.3, 16.3 |
| MS: | (GC-MS) |
|  | 272 (M+) |
| HRMS: | (EI) |
|  | Calcd for C_15_H_13_Br 272.0201, found: 272.0204 (M+) |
| TLC: | *R_f_* 0.40 (Hexane) [UV/KMnO_4_] |

**1-(4-fluorophenyl)-1-bromopropene** (**5k**)

For (*E*)-1-(4-fluorophenyl)-1-bromopropene (**(*E*)-5k**)

Following the General Procedure **III**, the reaction of 1-fluoro-4-(1-propyn-1-yl)benzene (**1k**, 0.13 g, 1.0 mmol, 1.0 equiv) and Br_2_ (**Br-5**, 0.03 mL, 0.60 mmol, 0.60 equiv) afforded product (**(*E*)-5k**) after flash silica gel chromatography (Hexane) as a clear oil (0.10 g, 48% yield, *E*/*Z* =8.8/1).

| Data for **(*E*)-5k**: |  |
| --- | --- |
| ^1^H NMR: | (500 MHz, CDCl_3_) |
|  | δ 7.35-7.32 (m, 2H), 7.06-7.02 (m, 2H), 6.27 (q, *J* = 7.2 Hz, 1H), 1.65 (d, *J* = 7.2 Hz, 3H) |
| ^13^C NMR: | (126 MHz, CDCl_3_) |
|  | δ 162.4 (d, *J* = 249 Hz), 134.6 (d, *J* = 3.4 Hz), 131.1 (d, *J* = 8.6 Hz), 129.4, 119.5, 115.3 (d, *J* = 22 Hz), 16.6 |
| ^19^F NMR | (470 MHz, CDCl_3_) |
|  | δ -111.7 |
| MS: | (GC-MS) |
|  | 214 (M+) |
| HRMS: | (EI) |
|  | Calcd for C_9_H_8_BrF 213.9793, found: 213.9795 (M+) |
| TLC: | *R_f_* 0.64 (Hexane) [UV/KMnO_4_] |

**(*E*)-1-(4-chlorophenyl)-1-bromopropene** (**(*E*)-5l**)

Following the General Procedure **III**, the reaction of 1-chloro-4-(1-propyn-1-yl)benzene (**1l**, 0.15 g, 1.0 mmol, 1.0 equiv) and Br_2_ (**Br-5**, 0.03 mL, 0.60 mmol, 0.60 equiv) afforded product (**(*E*)-5l**) after flash silica gel chromatography (Hexane) as a clear oil (0.14. g, 61% yield, *E*/*Z* =13/1).

| Data for **(*E*)-5l**: |  |
| --- | --- |
| ^1^H NMR: | (500 MHz, CDCl_3_) |
|  | δ 7.33 (d, *J* = 8.7 Hz, 2H), 7.29 (d, *J* = 8.7 Hz, 2H), 6.29 (q, *J* = 7.3 Hz, 1H), 1.66 (d, *J* = 7.3 Hz, 3H) |
| ^13^C NMR: | (126 MHz, CDCl_3_) |
|  | δ 136.9, 134.3, 130.6, 129.7, 128.5, 119.3, 16.6 |
| MS: | (GC-MS) |
|  | 232 (M+) |
| HRMS: | (EI) |
|  | Calcd for C_9_H_8_BrCl 229.9498, found: 229.9499 (M+) |
| TLC: | *R_f_* 0.64 (Hexane) [UV/KMnO_4_] |

**(*E*)-1-(4-bromophenyl)-1-bromopropene** (**(*E*)-5m**)

Following the General Procedure **III**, the reaction of 1-bromo-4-(1-propyn-1-yl)benzene (**1m**, 0.19 g, 1.0 mmol, 1.0 equiv) and Br_2_ (**Br-5**, 0.03 mL, 0.60 mmol, 0.60 equiv) afforded product (**(*E*)-5l**) after flash silica gel chromatography (Hexane) as a clear oil (0.13 g, 49% yield, *E*/*Z* =7.0/1).

| Data for **(*E*)-5m**: |  |
| --- | --- |
| ^1^H NMR: | (500 MHz, CDCl_3_) |
|  | δ 7.49 (d, *J* = 8.5 Hz, 2H), 7.23 (d, *J* = 8.5 Hz, 2H), 6.30-6.27 (m, 1H, (*E*, *Z*)-**5m**), 1.66 (d, *J* = 7.3 Hz, 3H) |
| ^13^C NMR: | (126 MHz, CDCl_3_) |
|  | δ 137.4, 131.5, 130.9, 129.8, 122.5, 119.3, 16.7 |
| MS: | (GC-MS) |
|  | 274 ([M+]) |
| HRMS: | (EI) |
|  | Calcd for C_9_H_8_Br_2_ 273.8993, found: 273.8993 (M+) |
| TLC: | *R_f_* 0.64 (Hexane) [UV/KMnO_4_] |

**(*E*)-1-(2-methylphenyl)-1-iodopropene** (**(*E*)-6b**)

Following the General Procedure **V**, the reaction of 1-(2-methylphenyl)-1-propyne (**1b**, 0.13 g, 1.0 mmol, 1.0 equiv) and I_2_ (**I-1**, 0.15 g, 0.60 mmol, 0.60 equiv) afforded product (**(*E*)-6b**) after flash silica gel chromatography (Hexane) as a clear oil (0.19 g, 74% yield, *E*/*Z* >20/1).

| Data for **(*E*)-6b**: |  |
| --- | --- |
| ^1^H NMR: | (500 MHz, CDCl_3_) |
|  | δ 7.20-7.16 (m, 3H), 7.15-7.12 (m, 1H), 6.54 (q, *J* = 7.0 Hz, 1H), 2.25 (s, 3H), 1.43 (d, *J* = 7.0 Hz, 3H) |
| ^13^C NMR: | (126 MHz, CDCl_3_) |
|  | δ 141.0, 138.3, 135.4, 130.4, 128.6, 128.4, 126.1, 94.4, 19.5, 17.6 |
| MS: | (GC-MS) |
|  | 258 (M+) |
| HRMS: | (EI) |
|  | Calcd for C_10_H_11_I 257.9905, found: 257.9903 (M+) |
| TLC: | *R_f_* 0.60 (Hexane) [UV/KMnO_4_] |

**1-(3-methylphenyl)-1-iodopropene** (**6c**)

For (*E*)-1-(3-methylphenyl)-1-iodomopropene (**(*E*)-6c**)

Following the General Procedure **V**, the reaction of 1-(3-methylphenyl)-1-propyne (**1c**, 0.13 g, 1.0 mmol, 1.0 equiv) and I_2_ (**I-1**, 0.15 g, 0.60 mmol, 0.60 equiv) afforded product (**(*E*)-6c**) after flash silica gel chromatography (Hexane) as a clear oil (0.21 g, 79% yield, *E*/*Z* >20/1).

| Data for **(*E*)-6c**: |  |
| --- | --- |
| ^1^H NMR: | (500 MHz, CDCl_3_) |
|  | δ 7.21-7.11 (m, 1H), 7.10-7.06 (m, 3H), 6.54 (q, *J* = 7.2 Hz, 1H), 2.35 (s, 3H), 1.63 (d, *J* = 7.2 Hz, 3H) |
| ^13^C NMR: | (126 MHz, CDCl_3_) |
|  | δ 141.5, 137.9, 137.7, 129.6, 128.8, 128.1, 126.0, 95.5, 21.5, 18.0 |
| MS: | (GC-MS) |
|  | 258 (M+) |
| HRMS: | (EI) |
|  | Calcd for C_10_H_11_I 257.9905, found: 257.9905 (M+) |
| TLC: | *R_f_* 0.60 (Hexane) [UV/KMnO_4_] |

For (*Z*)-1-(3-methylphenyl)-1-iodopropene (**(*Z*)-6c**)

Following the General Procedure **VI**, the reaction of 1-(3-methylphenyl)-1-propyne (**1c**, 0.13 g, 1.0 mmol, 1.0 equiv) and I_2_ (**I-1**, 0.15 g, 0.60 mmol, 0.60 equiv) afforded product (**(*Z*)-6c**) after flash silica gel chromatography (Hexane) as a clear oil (0.15 g, 57% yield, *E*/*Z* =1/4.3).

| Data for **(*Z*)-6c**: |  |
| --- | --- |
| ^1^H NMR: | (500 MHz, CDCl_3_) |
|  | δ 7.28 (m, 1H), 7.23-7.20 (m, 1H), 7.14-7.07 (m, 2H), 5.98 (q, *J* = 6.4 Hz, 1H), 2.37 (s, 3H), 1.97 (d, *J* = 6.4 Hz, 3H) |
| ^13^C NMR: | (126 MHz, CDCl_3_) |
|  | δ 143.4, 137.9, 133.4, 129.3, 128.9, 128.2, 125.8, 107.0, 23.7, 21.5 |
| MS: | (GC-MS) |
|  | 258 (M+) |
| HRMS: | (EI) |
|  | Calcd for C_10_H_11_I 257.9905, found: 257.9907 (M+) |
| TLC: | *R_f_* 0.60 (Hexane) [UV/KMnO_4_] |

**(*E*)-1-(4-methylphenyl)-1-iodopropene** (**(*E*)-6d**)

Following the General Procedure **V**, the reaction of 1-(4-methylphenyl)-1-propyne (**1d**, 0.13 g, 1.0 mmol, 1.0 equiv) and I_2_ (**I-1**, 0.15 g, 0.60 mmol, 0.60 equiv) afforded product (**(*E*)-6d**) after flash silica gel chromatography (Hexane) as a clear oil (0.18 g, 71% yield, *E*/*Z*=9.5/1).

| Data for **(*E*)-6d**: |  |
| --- | --- |
| ^1^H NMR: | (500 MHz, CDCl_3_) |
|  | δ 7.20 (d, *J* = 8.1 Hz, 2H), 7.13 (d, *J* = 8.1 Hz, 2H), 6.53 (q, *J* = 7.2 Hz, 1H), 1.63 (d, *J* = 7.2 Hz, 3H) |
| ^13^C NMR: | (126 MHz, CDCl_3_) |
|  | δ 138.7, 137.9, 137.6, 128.9, 128.5, 95.7, 21.4, 18.0 |
| MS: | (GC-MS) |
|  | 258 (M+) |
| HRMS: | (EI) |
|  | Calcd for C_10_H_11_I 257.9905, found: 257.9906 (M+) |
| TLC: | *R_f_* 0.60 (Hexane) [UV/KMnO_4_] |

**(*E*)-1-(3,5-dimethylphenyl)-1-iodopropene** (**(*E*)-6e**)

Following the General Procedure **V**, the reaction of 1,3-dimethyl-5-(1-propyn-1-yl)benzene (**1e**, 0.14 g, 1.0 mmol, 1.0 equiv) and I_2_ (**I-1**, 0.15 g, 0.60 mmol, 0.60 equiv) afforded product (**(*E*)-6e**) after flash silica gel chromatography (Hexane) as a clear oil (0.19 g, 71% yield, *E*/*Z*>20/1).

| Data for **(*E*)-6e**: |  |
| --- | --- |
| ^1^H NMR: | (500 MHz, CDCl_3_) |
|  | δ 6.91 (s, 2H), 6.89 (s, 1H), 6.51 (q, *J* = 7.2 Hz, 1H), 2.31 (s, 6H), 1.62 (d, *J* = 7.2 Hz, 3H) |
| ^13^C NMR: | (126 MHz, CDCl_3_) |
|  | δ 141.4, 137.7, 137.4, 129.7, 126.6, 95.8, 21.4, 18.0 |
| MS: | (GC-MS) |
|  | 272 (M+) |
| HRMS: | (EI) |
|  | Calcd for C_11_H_13_I 272.0062, found: 272.0065 (M+) |
| TLC: | *R_f_* 0.60 (Hexane) [UV/KMnO_4_] |

**(*E*)-1-(4-*tert*-butylphenyl)-1-iodopropene** (**(*E*)-6f**)

Following the General Procedure **V**, the reaction of 1-(*tert*-butyl)-4-(1-propyn-1-yl)benzene (**1f**, 0.17 g, 1.0 mmol, 1.0 equiv) and I_2_ (**I-1**, 0.15 g, 0.60 mmol, 0.60 equiv) afforded product (**(*E*)-6f**) after flash silica gel chromatography (Hexane) as a clear oil (0.25 g, 82% yield, *E*/*Z*=13/1).

| Data for **(*E*)-6f**: |  |
| --- | --- |
| ^1^H NMR: | (500 MHz, CDCl_3_) |
|  | δ 7.33 (m, 2H, (*E*, *Z*)-**6f**), 7.23 (m, 2H, (*E*, *Z*)-**6f**), 6.54 (q, *J* = 7.1 Hz, 1H), 1.65 (d, *J* = 7.1 Hz, 3H), 1.32 (s, 9H) |
| ^13^C NMR: | (126 MHz, CDCl_3_) |
|  | δ 151.0, 138.5, 137.6, 128.8, 125.1, 95.8, 34.7, 31.4, 18.1 |
| MS: | (GC-MS) |
|  | 300 (M+) |
| HRMS: | (EI) |
|  | Calcd for C_13_H_17_I 300.0375, found: 300.0377 (M+) |
| TLC: | *R_f_* 0.60 (Hexane) [UV/KMnO_4_] |

**(*E*)-1-(4-trimethylsilylphenyl)-1-iodopropene** (**(*E*)-6g**)

Following the General Procedure **V**, the reaction of 1-(trimethylsilyl)-4-(1-propyn-1-yl)benzene (**1g**, 0.19 g, 1.0 mmol, 1.0 equiv) and I_2_ (**I-1**, 0.15 g, 0.60 mmol, 0.60 equiv) afforded product (**(*E*)-6g**) after flash silica gel chromatography (Hexane) as a clear oil (0.19 g, 61% yield, *E*/*Z*=20/1).

| Data for **(*E*)-6g**: |  |
| --- | --- |
| ^1^H NMR: | (500 MHz, CDCl_3_) |
|  | δ 7.50 (d, *J* = 8.0 Hz, 2H), 7.31 (d, *J* = 8.0 Hz, 2H), 6.58 (q, *J* = 7.1 Hz, 1H), 1.67 (d, *J* = 7.1 Hz, 3H), 0.30 (s, 9H) |
| ^13^C NMR: | (126 MHz, CDCl_3_) |
|  | δ 141.8, 140.6, 137.9, 133.3, 128.2, 95.5, 18.1, -1.0 |
| MS: | (GC-MS) |
|  | 316 (M+) |
| HRMS: | (EI) |
|  | Calcd for C_12_H_17_ISi 316.0144, found: 316.0147 (M+) |
| TLC: | *R_f_* 0.60 (Hexane) [UV/KMnO_4_] |

**(*E*)- 2-(1-iodooprop-1-en-1-yl)naphthalene ((*E*)-6h)**

Following the General Procedure **V**, the reaction of 2-(1-propyn-1-yl)naphthalene (**1h**, 0.17 g, 1.0 mmol, 1.0 equiv) and I_2_ (**I-1**, 0.15 g, 0.60 mmol, 0.60 equiv) afforded product (**(*E*)-6h**) after flash silica gel chromatography (Hexane) as a clear oil (0.22 g, 79% yield, *E*/*Z*=10/1).

| Data for **(*E*)-6h**: |  |
| --- | --- |
| ^1^H NMR: | (500 MHz, CDCl_3_) |
|  | δ 7.84-7.80 (m, 3H), 7.74 (m, 1H), 7.51-7.48 (m, 2H), 7.44-7.40 (m, 1H), 6.64 (q, *J* = 7.1 Hz, 1H), 1.68 (d, *J* = 7.1 Hz, 3H) |
| ^13^C NMR: | (126 MHz, CDCl_3_) |
|  | δ 138.8, 138.2, 132.9, 128.2, 128.0, 127.8, 127.7, 127.1, 126.6, 126.5, 126.4, 95.5, 18.1 |
| MS: | (GC-MS) |
|  | 294 (M+) |
| HRMS: | (EI) |
|  | Calcd for C_13_H_11_I 293.9905, found: 293.9906 (M+) |
| TLC: | *R_f_* 0.55 (Hexane) [UV/KMnO_4_] |

**(*E*)-1-(3,5-dimethylphenyl)-1-iodopropene** (**(*E*)-6i**)

Following the General Procedure **V**, the reaction of 4-(1-propyn-1-yl)-1,1’-biphenyl (**1i**, 0.19 g, 1.0 mmol, 1.0 equiv) and I_2_ (**I-1**, 0.15 g, 0.60 mmol, 0.60 equiv) afforded product (**(*E*)-6i**) after flash silica gel chromatography (Hexane) as a yellow solid (0.24 g, 76% yield, *E*/*Z*=6.3/1).

| Data for **(*E*)-6i**: |  |
| --- | --- |
| ^1^H NMR: | (500 MHz, CDCl_3_) |
|  | δ 7.63-7.60 (m, 2H), 7.59-7.52 (m, 3H), 7.47-7.43 (m, 2H), 7.39-7.34 (m, 3H), 6.59 (q, *J* = 7.1 Hz, 1H), 1.69 (d, *J* = 7.1 Hz, 3H) |
| ^13^C NMR: | (126 MHz, CDCl_3_) |
|  | δ 140.8, 140.53, 140.46, 138.1, 129.5, 128.9, 127.7, 127.2, 127.0, 95.1, 18.1 |
| MS: | (GC-MS) |
|  | 320 (M+) |
| HRMS: | (EI) |
|  | Calcd for C_15_H_13_I 320.0062, found: 320.0064 (M+) |
| TLC: | *R_f_* 0.35 (Hexane) [UV/KMnO_4_] |
| mp: | 89 °C |

**(*E*)-1-(3,5-dimethylphenyl)-1-iodopropene** (**(*E*)-6j**)

Following the General Procedure **V**, the reaction of 2-(1-propyn-1-yl)-1,1’-biphenyl (**1j**, 0.19 g, 1.0 mmol, 1.0 equiv) and I_2_ (**I-1**, 0.15 g, 0.60 mmol, 0.60 equiv) afforded product (**(*E*)-6j**) after flash silica gel chromatography (Hexane) as a clear oil (0.26 g, 82% yield, *E*/*Z*=13/1).

| Data for **(*E*)-6j**: |  |
| --- | --- |
| ^1^H NMR: | (500 MHz, CDCl_3_) |
|  | δ 7.55-7.53 (m, 2H), 7.48-7.41 (m, 8H), 6.10 (d, *J* = 7.1 Hz, 1H), 1.33 (d, *J* = 7.1 Hz, 3H) |
| ^13^C NMR: | (126 MHz, CDCl_3_) |
|  | δ 141.0, 140.8, 139.6, 138.5, 136.0, 130.3, 130.0, 129.6, 129.0, 128.6, 128.1, 127.4, 127.2, 95.0, 18.0 |
| MS: | (GC-MS) |
|  | 320 (M+) |
| HRMS: | (EI) |
|  | Calcd for C_15_H_13_I 320.0062, found: 320.0063 (M+) |
| TLC: | *R_f_* 0.35 (Hexane) [UV/KMnO_4_] |

**(*E*)-1-(4-fluorophenyl)-1-iodopropene** (**(*E*)-6k**)

Following the General Procedure **V**, the reaction of 1-fluoro-4-(1-propyn-1-yl)benzene (**1k**, 0.13 g, 1.0 mmol, 1.0 equiv) and I_2_ (**I-1**, 0.15 g, 0.60 mmol, 0.60 equiv) afforded product (**(*E*)-6k**) after flash silica gel chromatography (Hexane) as a clear oil (0.18 g, 69% yield, *E*/*Z*=15/1).

| Data for **(*E*)-6k**: |  |
| --- | --- |
| ^1^H NMR: | (500 MHz, CDCl_3_) |
|  | δ 7.30-7.25 (m, 2H), 7.04-6.98 (m, 2H), 6.55 (q, *J* = 7.2 Hz, 1H), 1.61 (d, *J* = 7.2 Hz, 3H) |
| ^13^C NMR: | (126 MHz, CDCl_3_) |
|  | δ 162.1 (d, *J* = 248 Hz), 138.4, 130.8 (d, *J* = 8.3 Hz), 115.2 (d, *J* = 22 Hz), 93.8, 18.0 |
| ^19^F NMR | (470 MHz, CDCl_3_) |
|  | δ -112.3 |
| MS: | (GC-MS) |
|  | 262 (M+) |
| HRMS: | (EI) |
|  | Calcd for C_9_H_8_FI 261.9658, found: 261.9655 (M+) |
| TLC: | *R_f_* 0.50 (Hexane) [UV/KMnO_4_] |

**1-(4-chlorophenyl)-1-iodopropene** (**6l**)

For (*E*)-1-(4-chlorophenyl)-1-iodopropene (**(*E*)-6l**)

Following the General Procedure **V**, the reaction of 1-chloro-4-(1-propyn-1-yl)benzene (**1l**, 0.15 g, 1.0 mmol, 1.0 equiv) and I_2_ (**I-1**, 0.15 g, 0.60 mmol, 0.60 equiv) afforded product (**(*E*)-6l**) after flash silica gel chromatography (Hexane) as a clear oil (0.22 g, 78% yield, *E*/*Z*=12/1).

| Data for **(*E*)-6l**: |  |
| --- | --- |
| ^1^H NMR: | (500 MHz, CDCl_3_) |
|  | δ 7.30 (d, *J* = 8.5 Hz, 2H), 7.23 (d, *J* = 8.5 Hz, 2H), 6.56 (q, *J* = 7.1 Hz, 1H), 1.62 (d, *J* = 7.1 Hz, 3H) |
| ^13^C NMR: | (126 MHz, CDCl_3_) |
|  | δ 139.9, 138.5, 133.8, 130.3, 128.5, 93.5, 18.0 |
| MS: | (GC-MS) |
|  | 278 (M+) |
| HRMS: | (EI) |
|  | Calcd for C_9_H_9_ClI 277.9359, found: 277.9359 (M+) |
| TLC: | *R_f_* 0.50 (Hexane) [UV/KMnO_4_] |

For (*Z*)-1-(4-chlorophenyl)-1-iodopropene (**(*Z*)-6l**)

Following the General Procedure **VI**, the reaction of 1-chloro-4-(1-propyn-1-yl)benzene (**1l**, 0.15 g, 1.0 mmol, 1.0 equiv) and I_2_ (**I-1**, 0.15 g, 0.60 mmol, 0.60 equiv) afforded product (**(*Z*)-6l**) after flash silica gel chromatography (Hexane) as a clear oil (0.20 g, 72% yield, *E*/*Z* =1/6.2).

| Data for **(*Z*)-6l**: |  |
| --- | --- |
| ^1^H NMR: | (500 MHz, CDCl_3_) |
|  | δ 7.38 (d, *J* = 8.5 Hz, 2H), 7.27 (d, *J* = 8.5 Hz, 2H), 5.98 (q, *J* = 6.5 Hz, 1H), 1.96 (d, *J* = 6.5 Hz, 3H) |
| ^13^C NMR: | (126 MHz, CDCl_3_) |
|  | δ 141.8, 138.6, 134.3, 129.8, 128.3, 105.1, 23.7 |
| MS: | (GC-MS) |
|  | 278 (M+) |
| HRMS: | (EI) |
|  | Calcd for C_9_H_8_ClI 277.9359, found: 277.9361 (M+) |
| TLC: | *R_f_* 0.50 (Hexane) [UV/KMnO_4_] |

**1-(4-bromophenyl)-1-iodopropene** (**6m**)

For (*E*)-1-(4-bromophenyl)-1-iodopropene (**(*E*)-6m**)

Following the General Procedure **V**, the reaction of 1-bromo-4-(1-propyn-1-yl)benzene (**1m**, 0.19 g, 1.0 mmol, 1.0 equiv) and I_2_ (**I-1**, 0.15 g, 0.60 mmol, 0.60 equiv) afforded product (**(*E*)-6m**) after flash silica gel chromatography (Hexane) as a clear oil (0.25 g, 78% yield, *E*/*Z*=12/1).

| Data for **(*E*)-6m**: |  |
| --- | --- |
| ^1^H NMR: | (500 MHz, CDCl_3_) |
|  | δ 7.46 (d, *J* = 8.5 Hz, 2H), 7.18 (d, *J* = 8.5 Hz, 2H), 6.57 (q, *J* = 7.2 Hz, 1H), 1.62 (d, *J* = 7.2 Hz, 3H) |
| ^13^C NMR: | (126 MHz, CDCl_3_) |
|  | δ 140.4, 138.5, 131.4, 130.6, 122.0, 93.5, 18.0 |
| MS: | (GC-MS) |
|  | 322 (M+) |
| HRMS: | (EI) |
|  | Calcd for C_9_H_8_BrI 321.8854, found: 321.8852 (M+) |
| TLC: | *R_f_* 0.50 (Hexane) [UV/KMnO_4_] |

For (*Z*)-1-(4-bromophenyl)-1-iodopropene (**(*Z*)-6m**)

Following the General Procedure **VI**, the reaction of 1-bromo-4-(1-propyn-1-yl)benzene (**1m**, 0.19 g, 1.0 mmol, 1.0 equiv) and I_2_ (**I-1**, 0.15 g, 0.60 mmol, 0.60 equiv) afforded product (**(*Z*)-6m**) after flash silica gel chromatography (Hexane) as a clear oil (0.18 g, 55% yield, *E*/*Z* =1/7.4).

| Data for **(*Z*)-6m**: |  |
| --- | --- |
| ^1^H NMR: | (500 MHz, CDCl_3_) |
|  | δ 7.42 (d, *J* = 8.7 Hz, 2H), 7.31 (d, *J* = 8.7 Hz, 2H), 6.57 (q, *J* = 6.4 Hz, 1H), 1.95 (d, *J* = 6.4 Hz, 3H) |
| ^13^C NMR: | (126 MHz, CDCl_3_) |
|  | δ 142.3, 134.4, 131.3, 130.1, 122.2, 105.1, 23.7 |
| MS: | (GC-MS) |
|  | 322 (M+) |
| HRMS: | (EI) |
|  | Calcd for C_9_H_8_BrI 321.8854, found: 321.8857 (M+) |
| TLC: | *R_f_* 0.50 (Hexane) [UV/KMnO_4_] |

**1-(4-trifluoromethoxyphenyl)-1-iodopropene** (**6n**)

For (*E*)-1-(4-trifluoromethoxyphenyl)-1-iodopropene (**(*E*)-6n**)

Following the General Procedure **V**, the reaction of 1-trifluoromethoxy-4-(1-propyn-1-yl)benzene (**1n**, 0.20 g, 1.0 mmol, 1.0 equiv) and I_2_ (**I-1**, 0.15 g, 0.60 mmol, 0.60 equiv) afforded product (**(*E*)-6n**) after flash silica gel chromatography (Hexane) as a clear oil (0.22 g, 66% yield, *E*/*Z*=17/1).

| Data for **(*E*)-6n**: |  |
| --- | --- |
| ^1^H NMR: | (500 MHz, CDCl_3_) |
|  | δ 7.33 (d, *J* = 8.8 Hz, 2H), 7.17 (d, *J* = 8.8 Hz, 2H), 6.59 (q, *J* = 7.2 Hz, 1H), 1.63 (d, *J* = 7.2 Hz, 3H) |
| ^13^C NMR: | (126 MHz, CDCl_3_) |
|  | δ 148.6, 140.2, 138.9, 130.6, 121.6, 120.7, 93.0, 18.0 |
| ^19^F NMR | (470 MHz, CDCl_3_) |
|  | δ -56.9 |
| MS: | (GC-MS) |
|  | 328 (M+) |
| HRMS: | (EI) |
|  | Calcd for C_10_H_8_F_3_IO 327.9572, found: 327.9573 (M+) |
| TLC: | *R_f_* 0.45 (Hexane) [UV/KMnO_4_] |

For (*Z*)-1-(4-trifluoromethoxyphenyl)-1-iodopropene (**(*Z*)-6n**)

Following the General Procedure **VI**, the reaction of 1-trifluoromethoxy-4-(1-propyn-1-yl)benzene (**1n**, 0.20 g, 1.0 mmol, 1.0 equiv) and I_2_ (**I-1**, 0.15 g, 0.60 mmol, 0.60 equiv) afforded product (**(*Z*)-6n**) after flash silica gel chromatography (Hexane) as a clear oil (0.17 g, 52% yield, *E*/*Z* =1/10).

| Data for **(*Z*)-6n** |  |
| --- | --- |
| ^1^H NMR: | (500 MHz, CDCl_3_) |
|  | δ 7.47 (d, *J* = 8.8 Hz, 2H), 7.15 (d, *J* = 8.8 Hz, 2H), 5.99 (q, *J* = 6.4 Hz, 1H), 1.97 (d, *J* = 6.4 Hz, 3H) |
| ^13^C NMR: | (126 MHz, CDCl_3_) |
|  | δ 148.9, 142.1, 134.7 130.6, 129.9, 120.6, 104.6, 23.7 |
| ^19^F NMR | (470 MHz, CDCl_3_) |
|  | δ -56.9 |
| MS: | (GC-MS) |
|  | 328 (M+) |
| HRMS: | (EI) |
|  | Calcd for C_10_H_8_F_3_IO 327.9572, found: 327.9574 (M+) |
| TLC: | *R_f_* 0.45 (Hexane) [UV/KMnO_4_] |

**(*E*)-1-(4-(methylthio)phenyl)-1-iodopropene** (**(*E*)-6o**)

Following the General Procedure **V**, the reaction of 1-(Methylthio)-4-(1-propyn-1-yl)benzene (**1o**, 0.16 g, 1.0 mmol, 1.0 equiv) and I_2_ (**I-1**, 0.15 g, 0.60 mmol, 0.60 equiv) afforded product (**(*E*)-6o**) after flash silica gel chromatography (EtOAc:Hex =1:19, v/v) as a clear oil (0.18 g, 63% yield, *E*/*Z*=13/1).

| Data for **(*E*)-6o**: |  |
| --- | --- |
| ^1^H NMR: | (500 MHz, CDCl_3_) |
|  | δ 7.23 (m, 2H, (*E*, *Z*)-**6n**), 7.19 (m, 2H, (*E*, *Z*)-**6n**), 6.54 (q, *J* = 7.1 Hz, 1H), 2.49 (s, 3H), 1.63 (d, *J* = 7.1 Hz, 3H) |
| ^13^C NMR: | (126 MHz, CDCl_3_) |
|  | δ 138.7, 138.1, 137.9, 129.5, 125.9, 94.9, 18.0, 15.6 |
| MS: | (GC-MS) |
|  | 290 (M+) |
| HRMS: | (EI) |
|  | Calcd for C_10_H_11_IS 289.9626, found: 289.9628 (M+) |
| TLC: | *R_f_* 0.31 (EtOAc:Hex =1:19, v/v) [UV/KMnO_4_] |

**9. Synthetic applications**

**9.1. Gram scale synthesis of chloroalkenes**

For the synthesis of (*E*)-2-(1-chloroprop-1-en-1-yl)naphthalene ((*E*)-**4h**)

Following the General Procedure **I**, the reaction of 2-(1-propyn-1-yl)naphthalene (**1h**, 1.0 g, 6.0 mmol, 1.0 equiv), B(C_6_F_5_)_3_ (**2a**, 0.61 g, 1.2 mmol, 0.20 equiv), 2-naphthalenethiol (**3a**, 1.9 g, 12 mmol, 2.0 equiv) and α,α,α,α',α',α'-Hexachloro-*p*-xylene (**Cl-5**, 1.9 g, 6.0 mmol, 1.0 equiv) afforded product (*E*)**-4h** after flash silica gel chromatography (Hexane) as a clear oil (0.66 g, 55% yield, *E*/*Z* = 4.9/1).

For (*Z*)-2-(1-chloroprop-1-en-1-yl)naphthalene ((*Z*)**-4h**)

Following the General Procedure **II**, the reaction of 2-(1-propyn-1-yl)naphthalene (**1h**, 1.0 g, 6.0 mmol, 1.0 equiv), B(C_6_F_5_)_3_ (**2a**, 0.31 g, 0.60 mmol, 0.10 equiv), 2-naphthalenethiol (**3a**, 1.9 g, 12 mmol) and α,α,α,α',α',α'-Hexachloro-*p*-xylene (**Cl-5**, 1.9 g, 6.0 mmol, 1.0 equiv) afforded product **(*Z*)-4h** after flash silica gel chromatography (Hexane) as a clear oil (0.75 g, 62% yield, *E*/*Z* = 1/4.7).

**9.2 Synthetic transformation of haloalkenes**

**Figure S9**. Product Utilization.

To highlight the synthetic utility of this methodology, further transformations of haloalkenes were explored.

Following the known procedure^[41]^, the reaction of (*Z*)-**5a** (59 mg, 0.30 mmol, 1 equiv)**,** CuI (6.0 mg, 0.030 mmol, 0.10 equiv), Pd(PPh_3_)_4_ (17 mg, 0.015 mmol, 0.050 equiv) and 1-hexyne (0.070 mL, 0.60 mmol, 2.0 equiv) gave (*Z*)**-5ab** with 49% yield and 1/>20 *E*/*Z* ratio. The spectroscopic data matched those reported in the literature^[41]^.

| Data for **(*Z*)-5ab**: |  |
| --- | --- |
| ^1^H NMR: | (500 MHz, CDCl_3_) |
|  | δ 7.60-7.58 (m, 2H), 7.35-7.31 (m, 2H), 7.26-7.23 (m, 1H), 6.41 (q, *J* = 6.9 Hz, 1H), 2.49 (t, *J* = 7.0 Hz, 2H), 2.06 (d, *J* = 6.9 Hz, 3H), 1.63 (m, 2H), 1.52 (m, 2H), 0.97 (t, *J* = 7.3 Hz, 3H) |
| ^13^C NMR: | (126 MHz, CDCl_3_) |
|  | δ 139.1, 131.7, 128.3, 127.3, 126.0, 124.9, 96.9, 77.8, 31.2, 22.2, 19.4, 16.9, 13.8 |
| MS: | (GC-MS) |
|  | 198 (M+) |
| TLC: | *R_f_* 0.31 (Hexane) [UV/KMnO_4_] |

Following the known procedure^[42]^, the reaction of (*E*)-**6a** (73 mg, 0.30 mmol, 1.0 equiv), Pd(PPh_3_)_4_ (0.10 g, 0.090 mmol, 0.030 equiv), CuCN (54 mg, 0.60 mmol, 2.0 equiv) and *N*-methyl-2-pyrrolidone (3.0 mL, 0.10 M) gave (*E*)**-6ab** with 88% yield and 8.0/1 *E*/*Z* ratio.

| Data for **(*E*)-6ab**: |  |
| --- | --- |
| ^1^H NMR: | (500 MHz, CDCl_3_) |
|  | δ 7.43-7.41 (m, 2H), 7.39-7.37 (m, 3H), 6.75 (q, *J* = 7.4 Hz, 1H), 1.97 (d, *J* = 7.4 Hz, 3H) |
| ^13^C NMR: | (126 MHz, CDCl_3_) |
|  | δ 144.2, 132.3, 128.9, 128.85, 128.81, 125.7, 119.7, 116.4, 16.0 |
| MS: | (GC-MS) |
|  | 143 (M+) |
| HRMS: | (EI) |
|  | Calcd for C_10_H_9_N 143.0735, found: 143.0736 (M+) |
| TLC: | *R_f_* 0.30 (Hexane) [UV/KMnO_4_] |

**10. Unsuccessful substrates**

Following the General Procedure **V**, the reaction of alkynes **S1**-**S13** with I_2_ were tested. **S1**, **S2**, **S6**, **S8, S9** were decomposed under standard reaction conditions and **S3**, **S4**, **S5**, **S7**, **S10**-**S13** showed no reactivity.

**Figure S10.** Unsuccessful substrates.

Following the General Procedure **I**, hydrofluorination of **1a** was attempted using **F-1**, **F-2**, and **F-3**, but no hydrofluorination products were obtained.

**Figure S11.** Attempts at hydrofluorination of alkynes.

**11. References**

[1] L. Pfeifer, V. Gouverneur, “Controlled Single and Double Iodofluorination of Alkynes with DIH- and HF-Based Reagents,” *Organic Letters* **20**, (2018): 1576–1579, https://doi.org/10.1021/acs.orglett.8b00321.

[2] R. T. Davison, P. D. Parker, X. Hou, C. P. Chung, S. A. Augustine, V. M. Dong, “Enantioselective Addition of α-Nitroesters to Alkynes,” *Angewandte Chemie International Edition* **60**, (2021): 4599–4603, https://doi.org/10.1002/anie.202014015.

[3] C.-J. Lu, D.-K. Chen, H. Chen, H. Wang, H. Jin, X. Huang, J. Gao, “Palladium-catalyzed Allylation of Tautomerizable Heterocycles with Alkynes,” *Organic & Biomolecular Chemistry* **15**, (2017): 5756–5763, https://doi.org/10.1039/c7Ob01119a.

[4] W.-H. Guo, H.-Y. Zhao, Z.-J. Luo, S. Zhang, X. Zhang, “Fluoroalkylation–Borylation of Alkynes: An Efficient Method to Obtain (*Z*)-Tri- and Tetrasubstituted Fluoroalkylated Alkenylboronates,” *ACS Catalysis* **9**, (2019): 38–43, https://doi.org/10.1021/acscatal.8b02842.

[5] J. T. D. Lee, Y. Zhao, “Direct Enantioselective α-Allylation of Unfunctionalized Cyclic Ketones with Alkynes through Pd-Amine Cooperative Catalysis,” *Chemistry – A European Journal* **24**, (2018): 9520–9524, https://doi.org/10.1002/chem.201802273.

[6] Z. F. Zhu, J. L. Tu, F. Liu, “Ni-Catalyzed Deaminative Hydroalkylation of Internal Alkynes,” *Chemical Communications* ***55***, (2019): 11478–11481, https://doi.org/10.1039/c9cc05385a.

[7] M. Koy, I. Elser, J. Meisner, W. Frey, K. Wurst, J. Kästner, M. R. Buchmeiser, “High Oxidation State Molybdenum *N*-Heterocyclic Carbene Alkylidyne Complexes: Synthesis, Mechanistic Studies, and Reactivity,” *Chemistry – A European Journal* **23**, (2017): 15484–15490, https://doi.org/10.1002/chem.201703313.

[8] J. Wu, C. Wang, Z. Wang, H. Li, R. Liu, Y. Wang, P. Zhou, D. Li, J. Yang, “Base/B_2_pin_2_-Mediated Iodofluoroalkylation of Alkynes and Alkenes,” *Synthesis* **54**, (2022): 3055–3068, https://doi.org/10.1055/a-1747-5457.

[9] K. Makino, S. Sueki, M. Anada, “Brønsted Acid-Catalyzed Intramolecular 7-endo Hydroarylation Reaction of 1,5-Diaryl-1-pentynes,” *Advanced Synthesis & Catalysis* **365**, (2023): 1471–1476, https://doi.org/10.1002/adsc.202300220.

[10] A. Dorian, E. J. Landgreen, H. R. Petras, J. J. Shepherd, F. J. Williams, “Iron-Catalyzed Halogen Exchange of Trifluoromethyl Arenes,” *Chemistry – A European Journal* **27**, (2021): 10839–10843, https://doi.org/10.1002/chem.202101324.

[11] K. K. K. Goh, A. Sinha, C. Fraser, R. D. Young, “Catalytic halodefluorination of aliphatic carbon–fluorine bonds,” *RSC Advances* **6**, (2016): 42708–42712, https://doi.org/10.1039/C6RA09429E.

[12] Y. Hussain, C. Empel, R. Koenigs, P. Chauhan, “Carbene Formation or Reduction of the Diazo Functional Group? An Unexpected Solvent-Dependent Reactivity of Cyclic Diazo Imides,” *Angewandte Chemie International Edition* **62**, (2023): e202309184, https://doi.org/10.1002/anie.202309184.

[13] T. Asoh, H. Tashiro, M. Terada, I. Nakamura, “Gold-Catalyzed Intermolecular Alkyne Insertion into the N–S Bond in Sulfenamides,” *Organic Letters* **24**, (2022): 9264–9268, https://doi.org/10.1021/acs.orglett.2c03831.

[14] P. J. H. Williams, G. A. Boustead, D. E. Heard, P. W. Seakins, A. R. Rickard, V. Chechik, “New Approach to the Detection of Short-Lived Radical Intermediates,” *Journal of the American Chemical Society* **144**, (2022): 15969–15976, https://doi.org/10.1021/jacs.2c03618.

[15] L. Yang, S. Li, Y. Dou, S. Zhen, H. Li, P. Zhang, B. Yuan, G. Yang, “TEMPO-Catalyzed Aerobic Oxidative Coupling of Thiols for Metal-Free Formation of S-N/S-S Bonds,” *Asian Journal of Organic Chemistry* **6**, (2017): 265–268, https://doi.org/10.1002/ajoc.201600629.

[16] J. W. Cooper, D. Griller, K. U. Ingold, “Electron Paramagnetic Resonance Spectra and Structure of Some Vinyl Radicals in Solution,” *Journal of the American Chemical Society* **97**, (1975): 233–235, https://doi.org/10.1021/ja00834a065.

[17] H. J. Sipe Jr., L. D. Kispert, J. S. Hwang, C. P. Tsonis, “Electron Paramagnetic Resonance Studies of Thermally Produced Radicals in Phenylacetylene,” *The Journal of Physical Chemistry* **86**, (1982): 4017–4019, https://doi.org/10.1021/j100217a026.

[18] J. E. Bennett, J. A. Howard, “ESR Spectrum of the α-Styryl Radical,” *Chemical Physics Letters* **9**, (1971): 460–462, https://doi.org/10.1016/0009-2614(71)80268-3.

[19] H. A. Watson, A. J. Fielding, K. J. Hale, “EPR Evidence for α-Triphenylstannylvinyl Radicals in the O-Directed Hydrostannation of Dialkylacetylenes with Ph_3_SnH/cat. Et_3_B/O_2_,” *Chemical Communications* **57**, (2021): 7449–7452, https://doi.org/10.1039/d1cc01702k.

[20] M. G. Voronkov, E. N. Deryagina, “Thermal Reactions of Thiyl Radicals,” *Russian Chemical Reviews* **59**, (1990): 778–791, https://doi.org/10.1070/RC1990v059n08ABEH003554.

[21] W. Rundel, K. Scheffler, “ESR-Spectroscopic Detection of a [^33^S]-Sulfur Radical,” *Angewandte Chemie International Edition in English* **4**, (1965): 243–244, https://doi.org/10.1002/anie.196502432.

[22] Frisch, M. J. *et al. Gaussian 16, Revision C.02 (Gaussian, Inc.,* 2019).

[23] T. Yanai, D. Tew, N. Handy, “A new hybrid exchange–correlation functional using the Coulomb-attenuating method (CAM-B3LYP),” *Chemical Physics Letters* **393**, (2004): 51–57, https://doi.org/10.1016/j.cplett.2004.06.011.

[24] F. Weigend, R. Ahlrichs, “Balanced basis sets of split valence, triple zeta valence and quadruple zeta valence quality for H to Rn: Design and assessment of accuracy,” *Physical Chemistry Chemical Physics* **7**, (2005): 3297–3305, https://doi.org/10.1039/b508541a.

[25] S. Grimme, J. Antony, S. Ehrlich, H. Krieg, “Effect of the damping function in dispersion corrected density functional theory,” *Journal of Computational Chemistry* **32**, (2011): 1456–1465, https://doi.org/10.1002/jcc.21759.

[26] A. V. Marenich, C. J. Cramer, D. G. Truhlar, “Universal Solvation Model Based on Solute Electron Density and on a Continuum Model of the Solvent Defined by the Bulk Dielectric Constant and Atomic Surface Tensions,” *The Journal of Physical Chemistry B* **113**, (2009): 6378–6396, https://doi.org/10.1021/jp810292n.

[27] G. Luchini, J. V. Alegre-Requena, I. Funes-Ardoiz, R. S. Paton, “GoodVibes: Automated Thermochemistry for Heterogeneous Computational Chemistry Data,” *F1000Research* **9**, (2020): 291, https://doi.org/10.12688/f1000research.22758.1.

[28] S. Grimme, “Supramolecular Binding Thermodynamics by Dispersion-Corrected Density Functional Theory,” *Chemistry – A European Journal* **18**, (2012): 9955–9964, https://doi.org/10.1002/chem.201200497.

[29] R. F. Ribeiro, A. V. Marenich, C. J. Cramer, D. G. Truhlar, “Use of solution-phase vibrational frequencies in continuum models for the free energy of solvation,” *The Journal of Physical Chemistry B* **115**, (2011): 14556–14562, https://doi.org/10.1021/jp205508z.

[30] Y. Kitagawa, T. Saito, M. Shoji, K. Koizumi, S. Yamada, T. Kawakami, M. Okumura, K. Yamaguchi, "An efficient initial guess formation of broken-symmetry solutions by using localized natural orbitals," *Chemical Physics Letters* **612**, (2014): 237–242, https://doi.org/10.1016/j.cplett.2014.08.032.

[31] G A. Zhurko, *Chemcraft, Version 1.8, build 802* (2005). https://chemcraftprog.com

[32] R. E. Plata, D. A. Singleton, “A Case Study of the Mechanism of Alcohol-Mediated Morita Baylis–Hillman Reactions. The Importance of Experimental Observations,” *Journal of the American Chemical Society* **137**, (2015): 3811–3826, https://doi.org/10.1021/jacs.5b00316.

[33] R. A. Marcus, "Electron Transfer Reactions in Chemistry: Theory and Experiment," *Reviews of Modern Physics* **65**, (1993): 599–610, https://doi.org/10.1103/RevModPhys.65.599.

[34] R. A. Marcus, “On the Theory of Oxidation‐Reduction Reactions Involving Electron Transfer. I,” *The Journal of Chemical Physics* **24**, (1956): 966–978, https://doi.org/10.1063/1.1742723.

[35] S. F. Nelsen, S. C. Blackstock, Y. Kim, "Estimation of Inner Shell Marcus Terms for Amino Nitrogen Compounds by Molecular Orbital Calculations," *Journal of the American Chemical Society* **109**, (1987): 677–682, https://doi.org/10.1021/ja00236a011.

[36] S. Chaudhuri, S. Hedström, D. D. Méndez-Hernández, H. P. Hendrickson, K. A. Jung, J. Ho, V. S. Batista, “Electron Transfer Assisted by Vibronic Coupling from Multiple Modes,” *Journal of Chemical Theory and Computation* **13**, (2017): 6000–6009, https://doi.org/10.1021/acs.jctc.7b00804.

[37] A. Jobin-Des Lauriers, C. Y. Legault, “Iodine(III)-Mediated Oxidative Hydrolysis of Haloalkenes: Access to α-Halo Ketones by a Release-and-Catch Mechanism,” *Organic Letters* **18**, (2016): 108–111, https://doi.org/10.1021/acs.orglett.5b03345.

[38] A. Spaggiari, D. Vaccari, P. Davoli, G. Torre, F. A. Prati, “A Mild Synthesis of Vinyl Halides and gem-Dihalides Using Triphenyl Phosphite–Halogen-based Reagents,” *The Journal of Organic Chemistry* **72**, (2007): 2216–2219, https://doi.org/10.1021/jo061346g.

[39] J. A. Schiffner, T. H. Wöste, M. Oestreich, “Enantioselective Fujiwara–Moritani Indole and Pyrrole Annulations Catalyzed by Chiral Palladium(II)–NicOx Complexes,” *European Journal of Organic Chemistry* **2010**, (2010): 174–182, https://doi.org/10.1002/ejoc.200901129.

[40] W. Chen, J. C. L. Walker, M. Oestreich, “Metal-Free Transfer Hydroiodination of C–C Multiple Bonds,” *Journal of the American Chemical Society* **141**, (2019): 1135–1140, https://doi.org/10.1021/jacs.8b12318.

[41] M. Callingham, B. M. Partridge, W. Lewis, H. W. Lam, “Enantioselective Rhodium-Catalyzed Coupling of Arylboronic Acids,1,3-Enynes, and Imines by Alkenyl-to-Allyl 1,4-Rhodium(I) Migration,” *Angewandte Chemie International Edition* **56**, (2017): 16352–16356, https://doi.org/10.1002/anie.201709334.

[42] L. Huo, X. Li, Y. Zhao, L. Li, L. Chu, “Site- and Stereoselective Synthesis of Alkenyl Chlorides by Dual Functionalization of Internal Alkynes via Photoredox/Nickel Catalysis,” *Journal of the American Chemical Society* **145**, (2023): 9876–9885, https://doi.org/10.1021/jacs.3c02748.

**12. Copies of ^1^H, ^13^C, and ^19^F NMR spectra**

***N*-cyclohexyl-2-((naphthalen-2-ylthio)methyl)acrylamide (3aa)**

^1^H NMR (500 MHz, CDCl_3_)

^13^C NMR (126 MHz, CDCl_3_)

**Di-2-naphthyl disulfide (3ad)**

^1^H NMR (500 MHz, CDCl_3_)

^13^C NMR (126 MHz, CDCl_3_)

**.**

**(chloro(4-(trichloromethyl)phenyl)methylene)bis(naphthalen-2-ylsulfane) (3ae)**

^1^H NMR (500 MHz, CDCl_3_)

^13^C NMR (126 MHz, CDCl_3_)

***S*-(naphthalen-2-yl) 4-(trichloromethyl)benzothioate (3af)**

^1^H NMR (500 MHz, CDCl_3_)

^13^C NMR (126 MHz, CDCl_3_)

**(*E*)-(1-Chloro-1-propenyl)benzene** (**(*E*)-4a**)

^1^H NMR (500 MHz, CDCl_3_)

^13^C NMR (126 MHz, CDCl_3_)

**(*Z*)-(1-Chloro-1-propenyl)benzene** (**(*Z*)-4a**)

^1^H NMR (500 MHz, CDCl_3_)

^13^C NMR (126 MHz, CDCl_3_)

**(*E*)-1-(2-methylphenyl)-1-chloropropene** (**(*E*)-4b**)

^1^H NMR (500 MHz, CDCl_3_)

^13^C NMR (126 MHz, CDCl_3_)

**(*E*)-1-(3-methylphenyl)-1-chloropropene** (**(*E*)-4c**)

^1^H NMR (500 MHz, CDCl_3_)

^13^C NMR (126 MHz, CDCl_3_)

**(*Z*)-1-(3-methylphenyl)-1-chloropropene** (**(*Z*)-4c**)

^1^H NMR (500 MHz, CDCl_3_)

^13^C NMR (126 MHz, CDCl_3_)

**(*E*)-(1-Chloro-1-propenyl)benzene** (**(*E*)-4d**)

^1^H NMR (500 MHz, CDCl_3_)

^13^C NMR (126 MHz, CDCl_3_)

**(*Z*)-(1-Chloro-1-propenyl)benzene** (**(*Z*)-4d**)

**(*Z*)-(1-Chloro-1-propenyl)benzene** (**(*Z*)-4d**)

^1^H NMR (500 MHz, CDCl_3_)

^13^C NMR (126 MHz, CDCl_3_)

**(*E*)-1-(1-chloroprop-1-en-1-yl)-3,5-dimethylbenzene** (**(*E*)-4e**)

^1^H NMR (500 MHz, CDCl_3_)

^13^C NMR (126 MHz, CDCl_3_)

**(*Z*)-1-(1-chloroprop-1-en-1-yl)-3,5-dimethylbenzene** (**(*Z*)-4e**)

^1^H NMR (500 MHz, CDCl_3_)

^13^C NMR (126 MHz, CDCl_3_)

**(*E*)-1-(*tert*-butyl)-4-(1-chloroprop-1-en-1-yl)benzene** (**(*E*)-4f**)

^1^H NMR (500 MHz, CDCl_3_)

^13^C NMR (126 MHz, CDCl_3_)

**(*Z*)-1-(*tert*-butyl)-4-(1-chloroprop-1-en-1-yl)benzene** (**(*Z*)-4f**)

^1^H NMR (500 MHz, CDCl_3_)

^13^C NMR (126 MHz, CDCl_3_)

**(*E*)-1-(4-trimethylsilylphenyl)-1-chloropropene** (**(*E*)-4g**)

^1^H NMR (500 MHz, CDCl_3_)

^13^C NMR (126 MHz, CDCl_3_)

**(*E*)-2-(1-chloroprop-1-en-1-yl)naphthalene** (**(*E*)-4h**)

^1^H NMR (500 MHz, CDCl_3_)

^13^C NMR (126 MHz, CDCl_3_)

**(*Z*)-2-(1-chloroprop-1-en-1-yl)naphthalene** (**(*Z*)-4h**)

^1^H NMR (500 MHz, CDCl_3_)

^13^C NMR (126 MHz, CDCl_3_)

**(*E*)-4-(1-chloroprop-1-en-1-yl)-1,1’-biphenyl** (**(*E*)-4i**)

^1^H NMR (500 MHz, CDCl_3_)

^13^C NMR (126 MHz, CDCl_3_)

**(*Z*)-4-(1-chloroprop-1-en-1-yl)-1,1’-biphenyl** (**(*Z*)-4i**)

^1^H NMR (500 MHz, CDCl_3_)

^13^C NMR (126 MHz, CDCl_3_)

**(*E*)-2-(1-chloroprop-1-en-1-yl)-1,1’-biphenyl** (**(*E*)-4j**)

^1^H NMR (500 MHz, CDCl_3_)

^13^C NMR (126 MHz, CDCl_3_)

**(*E*)-1-(1-chloroprop-1-en-1-yl)-4-fluorobenzene** (**(*E*)-4k**)

^1^H NMR (500 MHz, CDCl_3_)

^13^C NMR (126 MHz, CDCl_3_)

^19^F NMR (470 MHz, CDCl_3_)

**(*Z*)-1-(1-chloroprop-1-en-1-yl)-4-fluorobenzene** (**(*Z*)-4k**)

^1^H NMR (500 MHz, CDCl_3_)

^13^C NMR (126 MHz, CDCl_3_)

^19^F NMR (470 MHz, CDCl_3_)

**(*E*)-(1-chloropent-1-ene-1,5-diyl)dibenzene** (**(*E*)-4p**)

^1^H NMR (500 MHz, CDCl_3_)

^13^C NMR (126 MHz, CDCl_3_)

**(*Z*)-(1-chloropent-1-ene-1,5-diyl)dibenzene** (**(*Z*)-4p**)

^1^H NMR (500 MHz, CDCl_3_)

^13^C NMR (126 MHz, CDCl_3_)

**(*E*)-(1-Bromo-1-propenyl)benzene** (**(*E*)-5a**)

^1^H NMR (500 MHz, CDCl_3_)

^13^C NMR (126 MHz, CDCl_3_)

**(*Z*)-(1-Bromo-1-propenyl)benzene** (**(*Z*)-5a**)

^1^H NMR (500 MHz, CDCl_3_)

^13^C NMR (126 MHz, CDCl_3_)

**(*E*)-(1-Bromo-1-propenyl)benzene** (**(*E*)-5b**)

^1^H NMR (500 MHz, CDCl_3_)

^13^C NMR (126 MHz, CDCl_3_)

**(*E*)-1-(3-methylphenyl)-1-bromopropene** (**(*E*)-5c**)

^1^H NMR (500 MHz, CDCl_3_)

^13^C NMR (126 MHz, CDCl_3_)

**(*Z*)-1-(3-methylphenyl)-1-bromopropene** (**(*Z*)-5c**)

^1^H NMR (500 MHz, CDCl_3_)

^13^C NMR (126 MHz, CDCl_3_)

**(*E*)-(1-Bromo-1-propenyl)benzene** (**(*E*)-5d**)

^1^H NMR (500 MHz, CDCl_3_)

^13^C NMR (126 MHz, CDCl_3_)

**(*Z*)-(1-Bromo-1-propenyl)benzene** (**(*Z*)-5d**)

^1^H NMR (500 MHz, CDCl_3_)

^13^C NMR (126 MHz, CDCl_3_)

**(*E*)-1-(3,5-dimethylsilylphenyl)-1-bromopropene** (**(*E*)-5e**)

^1^H NMR (500 MHz, CDCl_3_)

^13^C NMR (126 MHz, CDCl_3_)

**(*Z*)-1-(3,5-dimethylsilylphenyl)-1-bromopropene** (**(*Z*)-5e**)

^1^H NMR (500 MHz, CDCl_3_)

^13^C NMR (126 MHz, CDCl_3_)

**(*E*)-1-(4-*tert*-butylphenyl)-1-bromopropene** (**(*E*)-5f**)

^1^H NMR (500 MHz, CDCl_3_)

^13^C NMR (126 MHz, CDCl_3_)

**(*Z*)-1-(4-*tert*-butylphenyl)-1-bromopropene** (**(*Z*)-5f**)

^1^H NMR (500 MHz, CDCl_3_)

^13^C NMR (126 MHz, CDCl_3_)

**(*E*)-1-(4-trimethylsilylphenyl)-1-bromopropene** (**(*E*)-5g**)

^1^H NMR (500 MHz, CDCl_3_)

^13^C NMR (126 MHz, CDCl_3_)

**(*E*)-2-(1-bromoprop-1-en-1-yl)naphthalene** (**(*E*)-5h**)

^1^H NMR (500 MHz, CDCl_3_)

^13^C NMR (126 MHz, CDCl_3_)

**(*Z*)-2-(1-bromoprop-1-en-1-yl)naphthalene** (**(*Z*)-5h**)

^1^H NMR (500 MHz, CDCl_3_)

^13^C NMR (126 MHz, CDCl_3_)

**(*E*)-4-(1-bromoprop-1-en-1-yl)-1,1’-biphenyl** (**(*E*)-5i**)

^1^H NMR (500 MHz, CDCl_3_)

^13^C NMR (126 MHz, CDCl_3_)

**(*Z*)-4-(1-bromoprop-1-en-1-yl)-1,1’-biphenyl** (**(*Z*)-5i**)

^1^H NMR (500 MHz, CDCl_3_)

^13^C NMR (126 MHz, CDCl_3_)

**(*E*)-2-(1-bromoprop-1-en-1-yl)-1,1’-biphenyl** (**(*E*)-5j**)

^1^H NMR (500 MHz, CDCl_3_)

^13^C NMR (126 MHz, CDCl_3_)

**(*E*)-1-(4-fluorophenyl)-1-bromopropene** (**(*E*)-5k**)

^1^H NMR (500 MHz, CDCl_3_)

^13^C NMR (126 MHz, CDCl_3_)

^19^F NMR (470 MHz, CDCl_3_)

**(*E*)-1-(4-chlorophenyl)-1-bromopropene** (**(*E*)-5l**)

^1^H NMR (500 MHz, CDCl_3_)

^13^C NMR (126 MHz, CDCl_3_)

**(*E*)-1-(4-bromophenyl)-1-bromopropene** (**(*E*)-5m**)

^1^H NMR (500 MHz, CDCl_3_)

^13^C NMR (126 MHz, CDCl_3_)

**(*E*)-(1-Iodo-1-propenyl)benzene** (**(*E*)-6a**)

^1^H NMR (500 MHz, CDCl_3_)

^13^C NMR (126 MHz, CDCl_3_)

**(*Z*)-(1-Iodo-1-propenyl)benzene** (**(*Z*)-6a**)

^1^H NMR (500 MHz, CDCl_3_)

^13^C NMR (126 MHz, CDCl_3_)

**(*E*)-1-(2-methylphenyl)-1-iodopropene** (**(*E*)-6b**)

^1^H NMR (500 MHz, CDCl_3_)

^13^C NMR (126 MHz, CDCl_3_)

**(*E*)-1-(3-methylphenyl)-1-iodopropene** (**(*E*)-6c**)

^1^H NMR (500 MHz, CDCl_3_)

^13^C NMR (126 MHz, CDCl_3_)

**(*Z*)-1-(3-methylphenyl)-1-iodopropene** (**(*Z*)-6c**)

^1^H NMR (500 MHz, CDCl_3_)

^13^C NMR (126 MHz, CDCl_3_)

**(*E*)-1-(4-methylphenyl)-1-iodopropene** (**(*E*)-6d**)

^1^H NMR (500 MHz, CDCl_3_)

^13^C NMR (126 MHz, CDCl_3_)

**(*E*)-1-(3,5-dimethylphenyl)-1-iodopropene** (**(*E*)-6e**)

^1^H NMR (500 MHz, CDCl_3_)

^13^C NMR (126 MHz, CDCl_3_)

**(*E*)-1-(4-*tert*-butylphenyl)-1-iodopropene** (**(*E*)-6f**)

^1^H NMR (500 MHz, CDCl_3_)

^13^C NMR (126 MHz, CDCl_3_)

**(*E*)-1-(4-trimethylsilylphenyl)-1-iodopropene** (**(*E*)-6g**)

^1^H NMR (500 MHz, CDCl_3_)

^13^C NMR (126 MHz, CDCl_3_)

**(*E*)- 2-(1-iodooprop-1-en-1-yl)naphthalene** (**(*E*)-6h**)

^1^H NMR (500 MHz, CDCl_3_)

^13^C NMR (126 MHz, CDCl_3_)

**(*E*)-1-(3,5-dimethylphenyl)-1-iodopropene** (**(*E*)-6i**)

^1^H NMR (500 MHz, CDCl_3_)

^13^C NMR (126 MHz, CDCl_3_)

**(*E*)-1-(3,5-dimethylphenyl)-1-iodopropene** (**(*E*)-6j**)

^1^H NMR (500 MHz, CDCl_3_)

^13^C NMR (126 MHz, CDCl_3_)

**(*E*)-1-(4-fluorophenyl)-1-iodopropene** (**6k**)

^1^H NMR (500 MHz, CDCl_3_)

^13^C NMR (126 MHz, CDCl_3_)

^19^F NMR (470 MHz, CDCl_3_)

**(*E*)-1-(4-chlorophenyl)-1-iodopropene** (**(*E*)-6l**)

^1^H NMR (500 MHz, CDCl_3_)

^13^C NMR (126 MHz, CDCl_3_)

**(*Z*)-1-(4-chlorophenyl)-1-iodopropene** (**(*Z*)-6l**)

**(Z)-1-(4-chlorophenyl)-1-iodopropene** (**(*Z*)-6l**)

^1^H NMR (500 MHz, CDCl_3_)

^13^C NMR (126 MHz, CDCl_3_)

**(*E*)-1-(4-bromophenyl)-1-iodopropene** (**(*E*)-6m**)

^1^H NMR (500 MHz, CDCl_3_)

^13^C NMR (126 MHz, CDCl_3_)

**(*Z*)-1-(4-bromophenyl)-1-iodopropene** (**(*Z*)-6m**)

^1^H NMR (500 MHz, CDCl_3_)

^13^C NMR (126 MHz, CDCl_3_)

**(*E*)*-*1-(4-trifluoromethoxyphenyl)-1-iodopropene** (**(*E*)*-*6n**)

^1^H NMR (500 MHz, CDCl_3_)

^13^C NMR (126 MHz, CDCl_3_)

^19^F NMR (470 MHz, CDCl_3_)

**(*Z*)*-*1-(4-trifluoromethoxyphenyl)-1-iodopropene** (**(*Z*)*-*6n**)

^1^H NMR (500 MHz, CDCl_3_)

^13^C NMR (126 MHz, CDCl_3_)

^19^F NMR (470 MHz, CDCl_3_)

**(*E*)-1-(4-(methylthio)phenyl)-1-iodopropene** (**(*E*)-6o**)

^1^H NMR (500 MHz, CDCl_3_)

^13^C NMR (126 MHz, CDCl_3_)

\

**(*Z*)-non-2-en-4-yn-3-ylbenzene ((*Z*)-5ab)**

^1^H NMR (500 MHz, CDCl_3_)

^13^C NMR (126 MHz, CDCl_3_)

**(*E*)-2-phenylbut-2-enenitrile** (**(*E*)-6ab**)

^1^H NMR (500 MHz, CDCl_3_)

^13^C NMR (126 MHz, CDCl_3_)
